# Multifunctional Bio‐Based Packaging for Perishable Foods: Structural Design, Scalable Fabrication, and Versatile Applications

**DOI:** 10.1002/adma.202521880

**Published:** 2026-05-05

**Authors:** Lei Zhang, Jiaqi Zhang, Mei Cui, Wei Qi, Renliang Huang, Rongxin Su, Kai Zhang

**Affiliations:** ^1^ State Key Laboratory of Chemical Engineering and Low‐Carbon Technology Tianjin Key Laboratory of Membrane Science and Desalination Technology School of Chemical Engineering and Technology Tianjin University Tianjin P. R. China; ^2^ State Key Laboratory of Synthetic Biology School of Synthetic Biology and Biomanufacturing Tianjin University Tianjin P. R. China; ^3^ Tianjin Key Laboratory For Marine Environmental Research and Service School of Marine Science and Technology Tianjin University Tianjin P. R. China; ^4^ Zhejiang Institute of Tianjin University Ningbo Zhejiang P. R. China; ^5^ Sustainable Materials and Chemistry Department of Wood Technology and Wood‐based Nanocomposites University of Göttingen Göttingen Germany

**Keywords:** active packaging, bio‐based packaging, food safety and security, intelligent sensing, modified atmosphere packaging, radiative cooling, superhydrophobic surfaces

## Abstract

Food loss and food safety remain pressing global challenges, with roughly one‐third of food lost or wasted annually and approximately 420 000 deaths attributed to foodborne diseases. Conventional preservation and detection methods—such as refrigeration and centralized laboratory testing—are effective but often are energy‐ and infrastructure‐intensive, and provide delayed feedback. As a systems‐level alternative, multifunctional bio‐based packaging integrates two or more functions within and/or across preservation and quality monitoring, evolving from passive barriers into structure‐informed platforms that couple material chemistry, hierarchical architecture, and transport regulation. This review summarizes advances from 2020–2025 across radiative cooling systems, modified atmosphere packaging, active platforms, intelligent sensing labels, and superhydrophobic surfaces. We synthesize design principles through a structure–transport–function lens and emphasize commodity‐specific operating windows linking physiology to permeability/selectivity, release kinetics, and sensing reliability. To enable cross‐study comparison, we define integration paradigms (superposition, coupling, and quantitatively validated synergy) and adopt standardized benchmarking based on the shelf life multiplier. Translation readiness is assessed via scalable manufacturing, migration and biosafety, ISO‐aligned LCA, TEA, and consumer acceptance. Finally, we propose a food‐matrix‐informed framework for next‐generation bio‐based packaging that unites dynamic preservation and sensing with standardized validation and scalable green manufacturing, guiding rational development toward safer, more sustainable, and waste‐minimized food systems.

## Graphical abstract

An overview of design principles and scalable fabrication strategies for multifunctional bio‐based packaging. Radiative cooling films, modified‐atmosphere films/membranes, active antimicrobial/antioxidant platforms, intelligent optical/electrochemical labels, and superhydrophobic surfaces are co‐engineered from material chemistry to mesoscale structure to extend shelf life and enable real‐time quality monitoring.

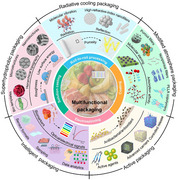

**Abbreviations**
3DAE‐Skin: 3‐dimensionally architected electronic skin
7‐HDCP: 7‐hydroxycoumarin quaternary phosphonium
ACQ: Aggregation‐caused quenching
AI: Artificial intelligence
AIEgens: Aggregation‐induced emission luminogen
AR: Acrylic resin
ASQ: 4‐(dimethylamino)styryl)quinoxalin‐2(1H)‐one
BAs: Biogenic amines
BBA: Blueberry anthocyanin
BC: Bacterial cellulose
BSA: Bovine serum albumin
CA: Cellulose acetate
Cad: Cadaverine
CAPEX and OPEX: Capital and operating expenditures
CATA: Check‐all‐that‐apply
CDs: Carbon dots
ChNFs: Chitin nanofibers
CNF: Cellulose nanofibril
CNNs: Convolutional neural networks
CNTs: Carbon nanotubes
COFs: Covalent organic framework
COPs: Covalent organic polymers
CSAs: Colorimetric sensor arrays
CSPMs: Chitosan porous microspheres
Cur‐PS: Curcumin‐loaded porous starch
DCNCs: Dialdehyde cellulose nanocrystals
DMA: Dimethylamine
EOs: Essential oils
ESG: Environmental, Social, and Governance
FITC: Fluorescein isothiocyanate
FRET: Fluorescence resonance energy transfer
FRJS: Focused rotary jet spinning
FTN: Food Technology Neophobia
GC/LC‐MS: Gas/liquid chromatography coupled with mass spectrometry
HCA: Hierarchical clustering analysis
HCOPs: Hydrogen‐bonded COPs
His: Histamine
HNTs: Halloysite nanotubes
HOFs: Hydrogen‐bonded organic frameworks
ICT: Intramolecular charge transfer
IoT: Internet of Things
LCA: Life cycle assessment
LDA: Linear discriminant analysis
LDPE: Low‐density polyethylene
MAP: Modified atmosphere packaging
MCC: Microcrystalline cellulose
MP: Mung‐bean protein
MSP: Minimum selling price
NADES: Natural deep eutectic solvent
nanoMOFs: Nanoscale metal–organic frameworks
NFC: Near‐field communication
NIAS: Non‐intentionally added substances
NIR: Near‐infrared
NN: Neural network
NPs: Nanoparticles
PBAT: Poly(butylene adipate‐co‐terephthalate)
PCA: Principal component analysis
PCR: Polymerase chain reaction
PDMS: Polydimethylsiloxane
PDT: Photodynamic therapy
PEDOT:PSS: Poly(3,4‐ethylenedioxythiophene):poly(styrenesulfonate)
PEI: Polyethyleneimine
PEO: Polyethylene oxide
PET: Photoinduced electron transfer
PET: Polyethylene terephthalate
PLA: Polylactic acid
PLLA: Poly(L‐lactic acid)
PpIX: Protoporphyrin IX
PPMs: Poly‐L‐lactic porous microspheres
PRC: Passive radiative cooling
psaFeN: Porous single‐atom iron nanozyme
PSs: Photosensitizers
PT: Potassium titanate
PTFE: Poly(tetrafluoroethylene)
PTL: Phase‐transitioned lysozyme
Put: Putrescine
PVA: Polyvinyl alcohol
R2R: Roll‐to‐roll
ROS: Reactive oxygen specie
SPI: Soybean protein isolate
SPS: Soybean polysaccharide
TA: Tannic acid
TEA: Techno‐economic analysis
TMA: Trimethylamine
TMB: 3,3′,5,5′‐tetramethylbenzidin
TMBox: TMB' oxidized form
TOC: Total antioxidant content
TVB‐N: Total volatile basic nitrogen
UCNPs: Upconversion nanoparticles
VOCs: Volatile organic compounds
γ‐CD MOFs: Nano γ‐cyclodextrin MOFs

## 1 Introduction

Global food systems are under mounting pressure to ensure food security and safety. In 2024, an estimated 8.2% of the global population suffered from hunger [1]. while nearly 10% experienced foodborne illness, causing ∼420 000 deaths annually [2]. Each year, ∼1.3 billion tons of food—roughly one‐third of global production—are wasted [3], driving over US$1 trillion in economic losses, 8%–10% of total greenhouse gas emissions, and the occupation of nearly 30% of agricultural land. Mitigating this waste could provide ∼1.3 meals per day to every undernourished person worldwide [4]. Foodborne diseases further intensify this crisis, encompassing over 200 illness types and 33 million healthy life years lost each year; children under five account for ∼40% of the burden and 125 000 deaths annually [5]. These realities elevate packaging from a logistical component to a strategic platform for public health, sustainability, and economic resilience.

Perishable products contribute disproportionately to these losses: ∼45% of fruits and vegetables, 35% of fish and seafood, and ∼20% of dairy and meat are discarded [6]. Spoilage originates from interconnected pathways—endogenous enzymatic reactions, microbial contamination, oxidative degradation, and temperature‐ or humidity‐driven mass transfer [7, 8]—which deteriorate sensory attributes (texture, color, and flavor) and deplete key nutrients (polyphenols, proteins, vitamins, and unsaturated lipids) [9, 10, 11, 12]. In low‐ and middle‐income regions, contaminated or expired food imposes ≈US$110 billion in annual losses from healthcare and productivity deficits [5, 13]. These challenges underscore the need for packaging that not only extends shelf life but also supports timely, in situ assessment of quality and safety.

Conventional preservation countermeasures—refrigeration, controlled atmospheres, irradiation, chemical preservatives, and waxing—can prolong shelf life [14, 15, 16, 17, 18], yet often demand high energy input, specialized infrastructure, or introduce residual‐chemical concerns [19, 20]. For instance, the cold chain alone consumes approximately 11% of global electricity and contributes to ∼2.5% of greenhouse‐gas emissions, while preserving ∼40% of the global food supply [21, 22]. Such reliance on energy‐ and infrastructure‐intensive preservation also creates vulnerability to logistics interruptions (e.g., loading/unloading and retail/household storage), where temperature excursions can rapidly erode quality. In parallel, traditional safety verification and quality assessment remain poorly suited to continuous, non‐destructive monitoring. Culture‐based microbiological methods—such as most probable number and viable counts (e.g., Petrifilm)—typically require 24–72 h to yield results and rely on destructive sampling [23]. Analytical techniques such as polymerase chain reaction (PCR) [24], mass spectrometry [25, 26], Raman spectroscopy [27], chromatography [28], ion mobility spectrometry [29], and dielectric analysis [30] can provide higher accuracy and sometimes faster assay times; however, they generally require specialized instrumentation and trained personnel and can be costly (commonly tens to hundreds of US dollars per sample), making them unsuitable for routine, on‐package, real‐time surveillance across distributed supply chains.

**FIGURE 1:**
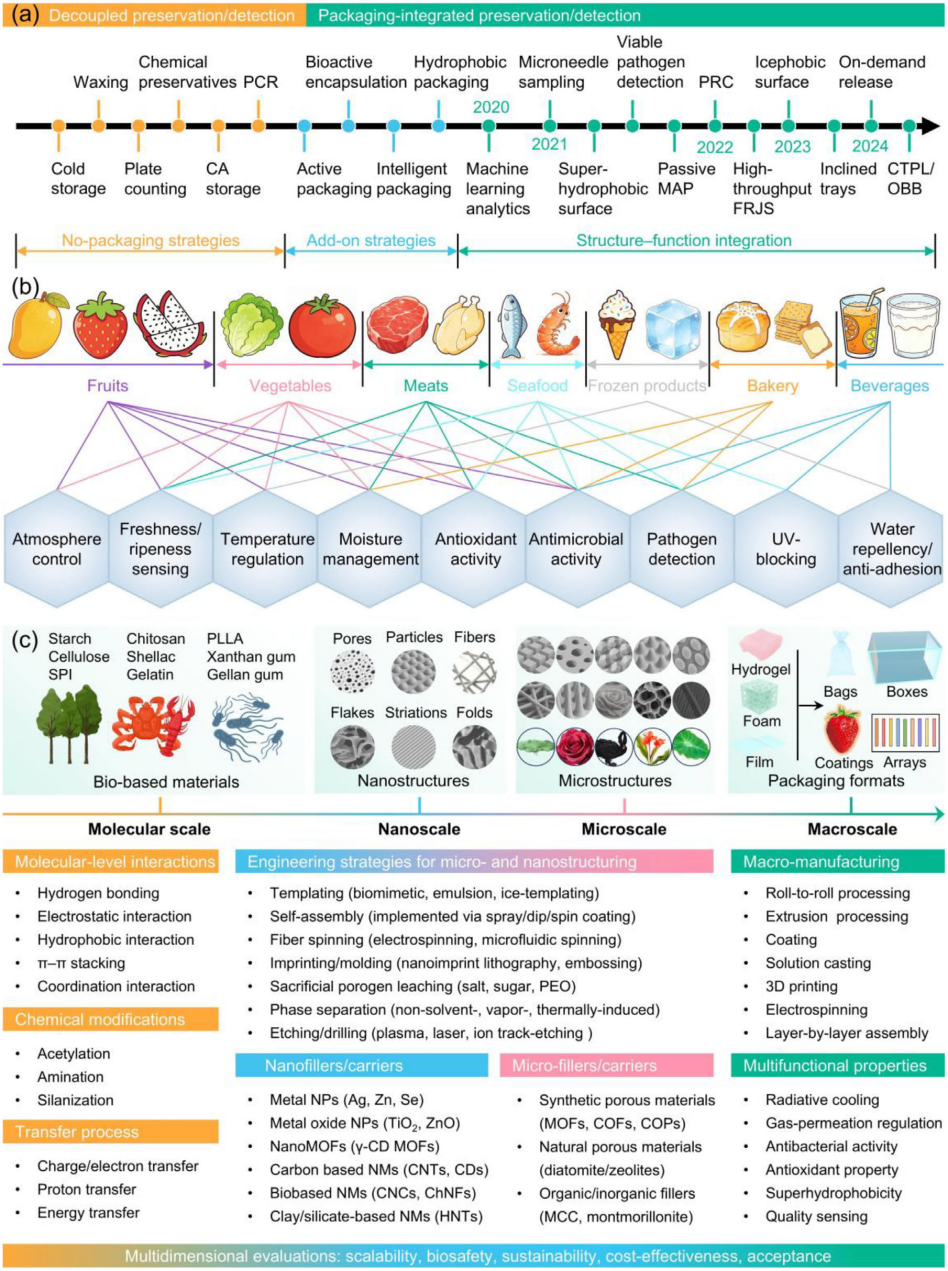
(a) Timeline of bio‐based packaging evolution for perishable foods (controlled atmosphere storage—CA storage, polymerase chain reaction—PCR, modified atmosphere packaging—MAP, passive radiative cooling—PRC, focused rotary jet spinning—FRJS, coating thickness power law—CTPL, oxygen‐barrier band—OBB). (b) Food‐matrix–dependent functional requirements: representative food categories are mapped to their dominant packaging needs, providing a practical starting point for designing and manufacturing multifunctional bio‐based packaging. (c) Framework linking design, fabrication, application, and evaluation of multifunctional systems (soybean protein isolate—SPI, poly (L‐lactic acid) —PLLA, polyethylene oxide—PEO, γ‐cyclodextrin MOFs—γ‐CD MOFs, carbon nanotubes—CNTs, carbon dots—CDs, cellulose nanocrystals—CNCs, chitin nanofibers—ChNFs, halloysite nanotubes—HNTs, metal‐organic frameworks—MOFs, covalent organic frameworks—COFs, covalent organic polymers—COPs).

These combined preservation and monitoring limitations motivated the 2010–2020 transition toward packaging that couples preservation with detection (Figure 1a). During this phase, packaging‐embedded preservation extended shelf life through distributed, low‐energy mechanisms—enhanced barriers, mechanical reinforcement, and antibacterial/antioxidant activity [31, 32]. Simultaneously, bio‐based polymers (e.g., cellulose, chitosan, proteins) emerged as renewable substitutes for petroleum plastics owing to their biodegradability and environmental benefits [33, 34, 35]. However, major hurdles persisted: insufficient mechanical robustness and moisture tolerance, short‐term efficacy of “add‐on” actives, oversimplified designs for multifactorial spoilage, economic and scalability constraints, weak structure–function integration, and inadequate validation of safety, migration, and end‐of‐life behavior [36, 37]. Early intelligent packaging incorporated indicators for temperature, leakage, freshness, or pH [38, 39, 40, 41], offering visual cues but often lacking quantitative precision, long‐term stability, versatile applicability, or sustainability [42]. As a result, research in this period remained fragmented and insufficiently aligned with industrial translation.

Since 2020, rationally designed, multifunctional, sustainability‐oriented bio‐based packaging has emerged through deeper structure‐informed integration (Figure 1a). In this context, “multifunctional” denotes the intentional integration of two or more functions—either within the preservation domain itself (e.g., antimicrobial/antioxidant protection combined with modified atmosphere regulation or radiative cooling) or across domains (e.g., preservation together with sensing and/or repellency)—within a single packaging system enabled by hierarchical design and multiscale structural engineering. Renewable feedstocks and upcycled agro‐wastes are increasingly adopted [43, 44], while bio‐based fillers such as nanocellulose and eggshell nanopowders [45, 46, 47] enhance mechanical robustness, barrier performance, and long‐term stability, advancing material circularity [48, 49]. Importantly, design has shifted from “add‐on” modification to coordinated tuning of architecture (e.g., porosity and multilayers), surface chemistry/morphology, and transport/optical behavior, enabling selective gas exchange [50], passive radiative cooling [21], superhydrophobicity [51], and controlled release [52]. Device‐level innovations further extend capability: microneedles and tray geometries enable in‐package sampling [53, 54], and moisture‐rich or porous matrices accelerate mass transfer for rapid sensing [55, 56]. As a result, multifunctional architectures increasingly combine preservation (barrier regulation; antimicrobial/antioxidant protection), sensing (freshness, contamination, anti‐counterfeiting), and repellency within gradient or Janus designs, thereby providing food‐matrix‐tailored protection across diverse commodities (e.g., high‐respiration produce, lipid‐rich meats/seafood, moisture‐sensitive bakery products, and liquid/viscous foods) (Figure 1b) [57, 58, 59, 60, 61]. Intelligent packaging has progressed from qualitative colorimetric labels to quantitative, multimodal platforms for biogenic amines (BAs) and volatile organic compounds (VOCs), with readouts spanning ratiometric fluorescence and electrical transduction to wireless modules (RFID/NFC) and smartphone‐based digitization [62, 63, 64]. Artificial intelligence (AI)‐enabled analytics can further improve calibration, drift correction, and predictive modeling of spoilage dynamics [23, 65, 66, 67, 68, 69]. Meanwhile, advances in scalable fabrication [45, 49], migration and biosafety evaluation [48, 70], life cycle assessment (LCA) [71], techno‐economic analysis (TEA) [48, 72], and consumer acceptance research [73, 74] have accelerated translation. Despite these achievements, the field still lacks a unified synthesis that connects food‐matrix needs, structure/transport design, scalable manufacturing, and standardized validation, hindering consistent scale‐up and regulatory alignment.

This review provides a comprehensive overview of multifunctional bio‐based packaging for perishable foods, emphasizing radiative cooling systems, modified atmosphere packaging, active platforms, intelligent sensing labels, and superhydrophobic surfaces. We distill structure‐informed design principles that couple chemistry with hierarchical architectures, and we discuss fabrication and translation strategies addressing stability, controllability, scalability, safety, and sustainability. We further synthesize application outcomes in shelf life extension and real‐time monitoring, including digital readouts and AI‐assisted analysis where appropriate. We discuss multifunctional integration in terms of function superposition and mechanistic coupling, and note that claims of synergy require quantitative validation under explicit baselines. Guided by a food‐matrix‐informed roadmap (Figure 1b) and an integrative framework (Figure 1c), we link commodity‐dependent functional needs with multiscale structural design, material functionality, and translation requirements to achieve system‐level performance. Finally, we outline priorities for deeper structure–transport–function understanding, standardized evaluation, regulatory readiness, and scalable green manufacturing. By uniting these directions, this review aims to support rational development and commercialization toward safer, more sustainable, and waste‐minimized food systems.

## 2 Multifunctional Bio‐Based Packaging

Multifunctional bio‐based packaging moves beyond passive containment by integrating tailored structures, advanced materials, engineering strategies, and digital technologies to enhance both preservation and detection. These innovations open new routes to mitigate food waste, reduce energy demand, and strengthen food security. The following subsections examine design principles, fabrication strategies, and practical applications across five major categories: radiative cooling, modified atmosphere, active, intelligent, and superhydrophobic systems.

### 2.1 Radiative Cooling Packaging

Refrigerated supply chains and cold storage are widely employed to suppress respiration and metabolic activity, thereby maintaining food quality, marketability, and extending shelf life [75]. Despite their effectiveness, cold chains are energy‐intensive and environmentally burdensome (see the Introduction for global electricity and emissions statistics). In many low‐ and middle‐income regions, inadequate refrigeration further amplifies losses during transport, retail, and storage [76]. Fresh products also require a narrow thermal window: excessively low temperatures induce chilling injury in cold‐sensitive commodities (surface pitting, browning, off‐flavors, abnormal ripening) [77, 78], whereas high temperatures and strong sunlight drive dehydration, deterioration, and sunburn [76]. Breaks in logistics (e.g., loading/unloading, household storage) trigger temperature excursions that undermine acceptability. These realities motivate energy‐efficient, sustainable, and easily deployable complements or alternatives to conventional refrigeration.

**FIGURE 2:**
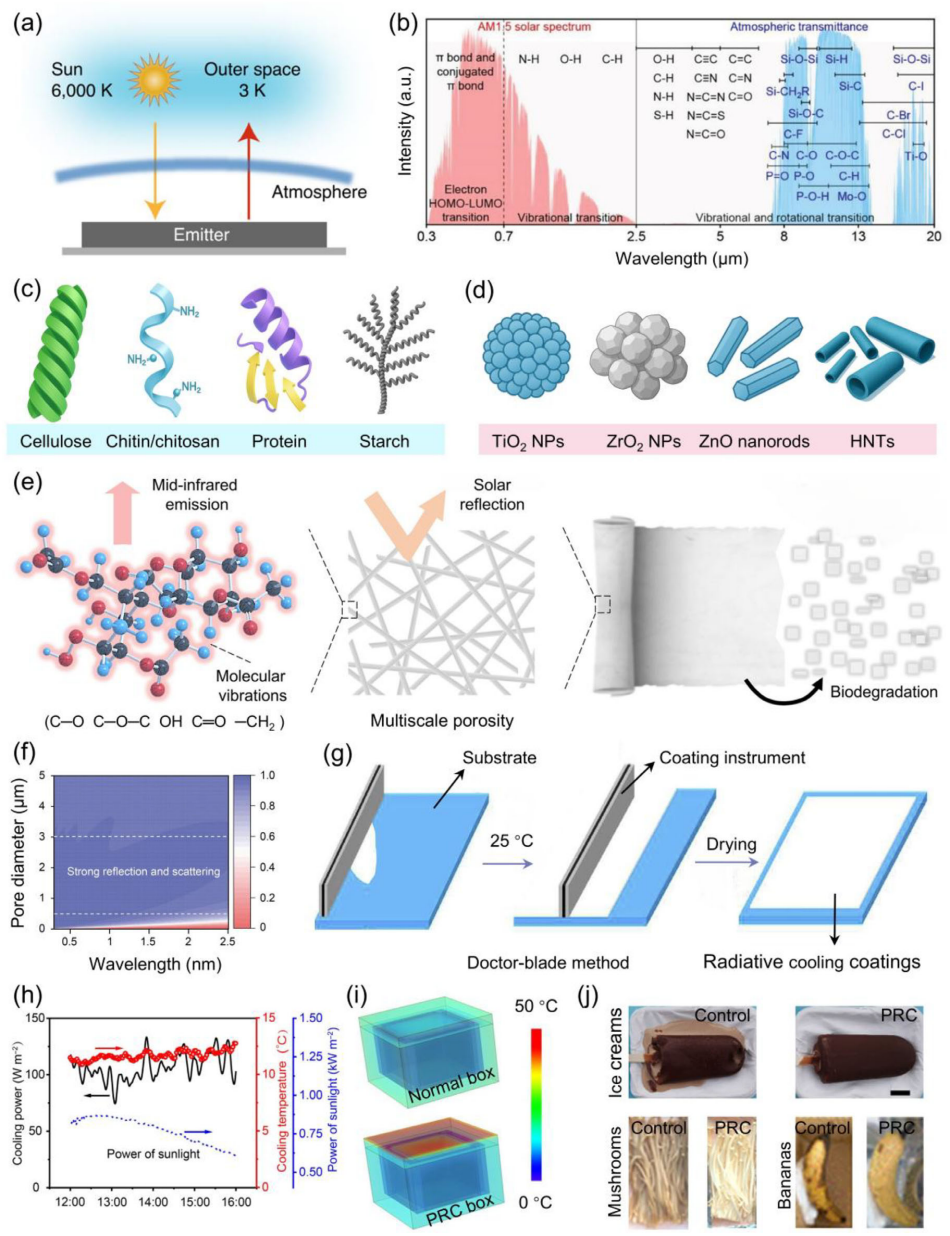
Design, fabrication, and applications of radiative cooling packaging. (a) Radiative heat‐exchange mechanism of radiative cooling. Reproduced with permission [93]. Copyright 2022, Springer Nature. (b) Spectra of chemical bonds or functional groups in the range of 0.3–20 µm. Reproduced with permission [94]. Copyright 2025, John Wiley and Sons. (c) Common bio‐based matrices for passive radiative cooling packaging. (d) Common high‐refractive‐index nanofillers to enhance solar reflectivity (halloysite nanotubes—HNTs). (e) Hierarchical design and life cycle of porous cellulose acetate passive radiative cooling film. (f) Theoretical analysis of the pore‐induced scattering; color bar indicates reflectivity. (g) Doctor‐blade deposition of PRC coatings on PET. Reproduced with permission [95]. Copyright 2024, Elsevier. (h) Cooling performance under sunlight referring to the ambient of the cellulose acetate passive radiative cooling film. (i) Surface‐temperature maps of a conventional vs passive radiative cooling‐insulated box after 36 h for ice protection. (j) Preservation outcomes for ice creams, vegetables, and fruits. Reproduced with permission [21]. Copyright 2022, The American Association for the Advancement of Science. Reproduced with permission [76]. Copyright 2023, American Chemical Society. Reproduced with permission [96]. Copyright 2024, American Chemical Society.

Unlike active cooling, passive radiative cooling (PRC) dissipates heat to outer space through the atmospheric transparency window (8–13 µm) while reflecting incident solar radiation, enabling sub‐ambient temperatures with zero energy input (Figure 2a,b) [79, 80]. PRC has been explored in vehicles [81, 82], wearable electronics [83, 84, 85], buildings [79, 86], and textiles [87]. With resource constraints tightening and carbon neutrality prioritized, biomass‐derived materials—gelatin, DNA, wood, and cellulose derivatives—have attracted attention for high‐performance PRC [21, 76, 79, 80, 88], making them promising for food preservation (Figure 2c and Table 1).

**TABLE 1:** Radiative cooling packaging for preserving perishable foods.

Packaging system	Food	R_solar_	ε_m id‐IR_	CP (W m^−2^)	∆T_max_ (°C)	Preservation effect	Refs.
CA	Ice; ice cream	0.974	0.92	110	12	Delayed melting; maintained appearance/integrity	[21]
CA/DMF/TiO_2_@PT	Lemon slice	0.976	0.95	/	6.5	Reduced water loss and structural shrinkage	[89]
CA/ZnO	Strawberry; enoki mushroom	0.97	0.94	/	13.8	Reduced shrinkage/rotting; strawberry shelf life up to 7 days	[76]
ZnO‐NRs/CM‐SM@ BBA	Cherry tomato; grape	0.944	0.988	/	6.5	Better color/taste; reduced bacteria growth; shelf life extended to 5–7 days	[90]
BC/TiO_2_	Lemon slice	0.891	0.946	98.7	7.15	Reduced water loss and shrinkage; improved freshness and appearance	[91]
Chitosan/PVA/TiO_2_ NPs	Strawberry; shimeji mushroom	/	/	/	6.4	Lower decay; high color saturation, water content, and aroma; strawberry shelf life extended to168 h	[92]
RC emitter/Al_2_O_3_	Banana; peach	0.92	0.84	/	9	Peach shelf life extended to 5 days; reduced respiration browning	[96]
HNTs/PVA/AR	Orange; tomato; kiwi	0.97	/	/	15	Reduced color change, decay and wrinkles; better freshness	[95]
CNF/TA/CA@e‐HNTs	Strawberry; *Agaricus bisporus*	0.905	0.94	/	6.2	Strawberry shelf life extended to 9 days; better surface/shape; optimal freshness	[88]
ZrO_2_ NPs/NADES@PAAm/PVA	Pear; Fuji apple	0.89	0.90	87	15.3	Reduced discoloration; better cell membrane integrity	[97]
ZrO_2_ NPs/NADES@PAAm/PVA	/	0.91	0.90	/	11.7	/	[98]

R_solar_, solar reflectivity. 𝜀_mid‐IR_, mid‐infrared emissivity. CP, cooling power. ∆T_max_, maximum cooling temperature.

#### 2.1.1 Radiative Cooling Films

Bio‐based PRC films typically combine (i) intrinsic mid‐IR emissivity from polymer vibrational modes with (ii) solar back‐scattering enabled by hierarchical porosity and/or high‐refractive‐index fillers. Roll‐to‐roll electrospun cellulose acetate (CA) films with hierarchical porosity (500 nm–3 µm) enable passive radiative cooling for ice‐food protection under sunlight (Figure 2e) [21]. CA molecular vibrational modes afforded broadband, high mid‐infrared emissivity (0.92), while multiscale porosity enhanced solar back‐scattering (solar reflectivity 0.974) (Figure 2f), yielding cooling power up to ∼110 W m^−2^ and a ∼12°C temperature drop under direct sunlight (Figure 2h). Wrapped iced‐foods remained below 0°C for 5.5 h; ice cream retained ∼98% integrity after 80 min sunlight exposure, demonstrating an effective and sustainable route to ice‐food preservation (Figure 2j) [21]. Durability, however, was limited by porosity‐induced mechanical weakness and the intrinsic UV susceptibility of CA matrix. To address these issues while preserving porosity, high‐refractive‐index nanofillers (Figure 2d) were introduced via solvent‐induced phase separation [76, 89]. TiO_2_@potassium titanate (PT) nanofiller improved mechanical performance, photostability, solar reflectivity (0.976), and mid‐infrared emissivity (0.95), achieving daytime cooling of 6.5°C and reducing weight loss and shrinkage of lemon slices under direct sunlight [89]. ZnO nanoparticles (NPs) likewise increased solar reflectivity and mid‐infrared emissivity to 0.97 and 0.94, respectively, delivering up to 13.8°C daytime cooling; ZnO also imparted antibacterial activity and, together with the micro/nanostructure, enhanced mechanical properties and weatherability, maintaining the freshness of fruits and vegetables (Figure 2j) [76]. ZnO nanorods hydrothermally grown on cellulose membranes further enabled tunable porous architectures that strengthen solar scattering; the resulting composites exhibited high solar reflectivity (0.944) and mid‐infrared emissivity (0.988), realizing 6.5°C cooling and prolonging the shelf life of fresh products, while incorporation of blueberry anthocyanins (BBA) added pH sensitivity for real‐time monitoring of grape freshness [90]. Leveraging the high purity and 3D network of bacterial cellulose (BC), TiO_2_ NPs were incorporated via casting followed by polydimethylsyiloxane (PDMS) spraying to form porous radiative cooling films (mid‐infrared emissivity 0.946; solar reflectivity 0.891) with a maximum temperature drop of 7.15°C and improved lemon freshness under intense sunlight [91]. Chitosan was also employed to fabricate PRC films with inherent antibacterial activity. To obtain on‐demand microporosity, a particle leaching method removed NaCl and polyethylene from cross‐linked chitosan/polyvinyl alcohol (PVA) matrices, generating 0.1–350 µm pores at 98.14% porosity [92]. Incorporation of TiO_2_ NPs further enhanced antibacterial performance, UV shielding, and radiative cooling efficacy, achieving ∼6.4°C cooling and extending strawberry shelf life to 168 h.

#### 2.1.2 Radiative Cooling Coatings

Compared with freestanding films, PRC coatings provide higher process compatibility and mechanical durability for practical cold‐chain deployment. A representative approach uses blade‐coated formulations on metal sheets, where reflective substrates and scattering fillers maintain high solar reflectivity, while emissive components enhance mid‐IR radiation; abrasion‐resistant phases help retain optical performance after wear (solar reflectivity 0.92; mid‐infrared emissivity 0.84), enabling packaging boxes that buffer temperature excursions and extend the shelf life of bananas and peaches (Figure 2i,j) [96]. PRC coatings can also be applied onto commodity plastics through interfacial hydrogen‐bonding and hydrophobic interactions (Figure 2g) [88, 95]. A water‐based halloysite nanotubes (HNTs)/PVA/acrylic resin (AR) micro‐coating (∼6.2 µm) on polyethylene terephthalate (PET) achieves high whiteness (∼95%), solar reflectivity of 0.97, and up to 15°C cooling under simulated sunlight through multiple scattering and micro‐roughness/porosity, while remaining compatible with screen printing and stable across pH 1–13 and 60–250°C [95]. In addition, spray‐coated HNT/chitin nanofiber (ChNFs)/tannic acid (TA) systems on poly(butylene adipate‐co‐terephthalate) (PBAT) strengthen adhesion via combined hydrogen‐bonding/hydrophobic interactions; chitin vibrational modes and HNT phonon‐polariton resonances yield mid‐infrared emissivity of 0.94, while porous/rough morphology raises solar reflectivity to 0.905, achieving 6.2°C cooling and extending strawberry shelf life to 9 days [88].

#### 2.1.3 Multimodal Cooling Systems

Single‐mode radiative cooling may not always meet preservation requirements, motivating radiative–evaporative hybrid designs that leverage water uptake/evaporation to raise the cooling ceiling. A representative hydrogel system based on a one‐pot polyacrylamide/PVA network (initially formulated with a natural deep eutectic solvent, NADES) integrates scattering/emitting components: ZrO_2_ is incorporated to enhance solar reflectance, while poly(tetrafluoroethylene) (PTFE) increases thermal emittance, achieving a solar reflectivity of 0.89 and a mid‐infrared emissivity of 0.90 [97]. After hydration, the coupled radiative–evaporative mechanism enables a temperature reduction of ∼15.3°C under sunlight, alleviating sunburn risk and maintaining pear quality comparable to fresh controls after 2 h exposure. A subsequent, bio‐storage‐oriented formulation eliminated NADES while retaining the hybrid concept, delivering a solar reflectivity of 0.91 and mid‐infrared emissivity of 0.90, and mitigating polyphenol loss and enzyme degradation during storage [98].

Cooling performance of radiative cooling materials is governed by chemical composition and multiscale architecture. Matrix polymers with favorable vibrational modes—such as CA and chitosan—provide strong mid‐infrared emissivity, whereas porous/rough structures and high‐refractive‐index nanofillers (e.g., TiO_2_, Al_2_O_3_, ZnO) enhance solar back‐scattering and thus solar reflectivity. Guided by structure–property–function relationships, these strategies frequently yield daytime temperature reductions exceeding 5°C under solar irradiation, and can be further amplified by hybrid radiative–evaporative concepts to strengthen cooling. For food packaging, material choice requires balancing optical performance against durability, processability, and safety. Among various biopolymers, cellulose and its derivatives are attractive matrices because C–OH and C–O–C vibrations support high emissivity, and their hierarchical micro/nanostructures promote efficient solar scattering; however, intrinsic hydrophilicity can lead to moisture absorption that degrades optical, mechanical, and cooling performance. Chitin and chitosan offer intrinsic antimicrobial activity—particularly valuable for food packaging—and their nanofibrous architecture can generate strong Mie scattering, providing a pathway to high solar reflectance when suitable micro/nanotextures are engineered; practical deployment, however, is constrained by high processing costs (demineralization, deproteinization, and deacetylation) and limited solubility/processable windows. Protein matrices such as silk fibroin exhibit broad mid‐IR vibrational bands (amide I at ∼6 µm to amide V at ∼12–20 µm), but absorption of downward atmospheric radiation outside 8–13 µm window can limit sub‐ambient cooling, and the labor‐intensive sericulture process makes natural silk costly and difficult to scale [99].

Beyond the matrix choice, scattering fillers introduce an additional performance–practicality trade‐off. High‐refractive‐index particles such as TiO_2_ (*n* ≈2.74) and ZnO (*n* ≈2.0) provide strong backscattering at relatively low loadings but may absorb UV light due to their smaller bandgaps (<4.13 eV), which can partially offset cooling unless mitigated by protective design. By contrast, wide‐bandgap alternatives including Al_2_O_3_ (≈8.7 eV), SiO_2_ (≈8.4 eV), and BaSO_4_ (≈6 eV) exhibit negligible UV absorption and can improve durability and safety; but their lower refractive indices (Al_2_O_3_, *n* ≈1.77; SiO_2_, *n* ≈1.45; BaSO_4_, *n* ≈1.64) typically require higher loadings and/or microstructure optimization (e.g., porosity control or tailored particle‐size distributions) to reach comparable scattering efficiency [94]. Finally, both the green scale‐up challenges associated with solvent‐intensive porous‐polymer routes and the non‐negotiable food‐contact biosafety requirements are further discussed in Section 3.

Industrial translation also requires evaluating cooling performance under real‐world logistics constraints. Cooling effectiveness depends on sky exposure; when packages are stacked, wrapped, or enclosed, the effect is primarily observed on exposed outer surfaces and overall efficiency is reduced. Nevertheless, even modest temperature reductions (e.g., 1–2°C) can slow respiration, microbial growth, and oxidation, helping preserve quality and extend shelf life. Accordingly, radiative cooling is better positioned as a complementary strategy—rather than a replacement for active refrigeration—and can be combined with other preservation functions (e.g., barrier/MAP/active packaging) to broaden robustness across variable scenarios. Environmental fouling—such as dust accumulation, surface contamination, or biofilm formation during logistics—can further reduce solar reflectance (and sometimes mid‐IR performance), suggesting value in multifunction integration—for example, self‐cleaning or superhydrophobic surfaces (Section 2.5)—which remains underexplored for food‐packaging‐oriented radiative cooling systems. Moreover, practical deployment also requires sufficient mechanical robustness to withstand handling and transport, UV and abrasion stability to endure outdoor exposure, and reliable adhesion to common substrates (e.g., paperboard, plastics, or bio‐based films), while remaining compatible with scalable fabrication (blade/spray coating, roll‐to‐roll processing). Standardized testing with in situ temperature/humidity logging under realistic stacking and airflow conditions is also essential for validation and translation. Economic feasibility is equally important; however, no techno‐economic analysis specifically for radiative cooling packaging has been reported. We therefore draw on TEA results of PRC materials for building applications [100, 101] and established TEA workflows of other packaging materials [48], and highlight key needs for future analysis, including manufacturing cost (e.g., US$ m^−2^ and throughput) and scenario‐based comparison with conventional cold‐chain preservation. These considerations are further discussed in Section 3.

### 2.2 Modified Atmosphere Packaging

An atmosphere with reduced oxygen and elevated carbon dioxide suppresses physiological metabolism and inhibits microbial proliferation, thereby extending the shelf life of perishables. Under ambient air (≈20.95% O_2_, ≈0.03% CO_2_), postharvest respiration remains intensive, driving nutrient consumption, water loss, flavor/color changes, and spoilage in fruits and vegetables [102, 103]. Conversely, excessively low O_2_ and high CO_2_ can trigger anaerobic respiration and stress responses, accelerating senescence and causing off‐flavors and physiological disorders [45, 104, 105]. To maintain a suitable gas microenvironment, controlled atmosphere storage injects gas continuously into large enclosures, but the requirement for fixed infrastructure and continuous gas supply limits practicality in many regions [18, 106]. Demands also differ between non‐climacteric and climacteric produce, and respiration‐driven fluctuations can undermine stability. By contrast, modified atmosphere packaging (MAP) tailors headspace composition at the package scale and is typically more cost‐effective [107]. Passive MAP relies on the interplay between commodity respiration (O_2_ consumption/CO_2_ generation) and film permeability; a dynamic steady state forms when O_2_ ingress and CO_2_ egress balance metabolic rates, yielding an atmosphere that slows deterioration. For many fruits and vegetables, 2%–6% O_2_ and 2%–15% CO_2_ are suitable ranges [108, 109]. Achieving and stabilizing this window requires films with appropriate permeability and elevated CO_2_/O_2_ selectivity (Table 2). Traditional perforation offers exchange channels but typically yields CO_2_/O_2_ selectivity ≈1, motivating structure–material strategies that decouple permeability and selectivity.

**TABLE 2:** Modified atmosphere packaging for preserving perishable foods.

Packaging system	Food	CO_2_/O_2_ selectivity	O_2_ permeability (mol m^−1^ s^−1^ Pa^−1^)	CO_2_ permeability (mol m^−1^ s^−1^ Pa^−1^)	Preservation effect	Refs.
TA‐CSPM/shellac	Orange; mango; waxberry; strawberry; cherry	5.8–8.6	1.39 × 10^−16^–2.65 × 10^−16^	7.87 × 10^−16^–21.92 × 10^−16^	Reduced respiration rate and weight loss; shelf‐life extended to 60 and 72 h (strawberry and cherry); better quality and edibility	[110]
PPM/shellac		6.14–7.52	1.63 × 10^−16^–3.32 × 10^−16^	9.55×10^−16^–23.89×10^−16^		
TA‐CPM/shellac	Litchi	/	/	/	Reduced browning and rotting; better appearance and quality; shelf‐life extended to 8 days	[107]
Cur‐PS/chitosan	Cherry; fresh‐cut apple slice	2.60–5.51	6.25 × 10^−14^–79.3 × 10^−14^	2.50 × 10^−13^–3.35 × 10^−13^	Reduced respiration and metabolism; shelf life extended to 5 and 2 days	[111]
Nano MOFs/CMC/Zein	Mango	3.55–8.65	8.44 × 10^−13^–2.87 × 10^−12^	4.69 × 10^−12^–5.68 × 10^−12^	Reduced molds, browning, and weight loss; shelf life extended to 7 days	[115]
LT‐HCOPs/PAN	Strawberry; waxberry; cherry; cherry tomato; mango	18.46	/	/	Better freshness and edibility; reduced molds, browning and weight loss; shelf life extended to 10–12 days	[112]
DE‐g‐PEI/CNF	Litchi; green plum	0.95–6.32	/	/	Reduced browning, respiration, metabolism and flesh softening	[103]
Chitosan /DCNC/CDs	Winter jujube	12.8–14.1	5.62 × 10^−14^–7.16 × 10^−14^	5.73 × 10^−13^–6.66 × 10^−13^	Reduced discoloration and flesh browning; delayed ripening and aging	[113]
SPI nanofibers/chitosan	Cherry; strawberry	>100	1.98 × 10^−18^–2.60 × 10^−18^	2.44 × 10^−16^–2.71 × 10^−16^	Reduced chemical and bacterial deterioration; Edible rate ≈75% after 7 and 5 days	[114]
MP fibrils/chitosan	Fresh tea leaves; strawberry	130	0.78 × 10^−16^–2.33 × 10^−16^	2.55 × 10^−15^–3.22 × 10^−15^	Edible rate ≈85% after 9 days; reduced molds, decay, water loss, polyphenol and amino acid loss	[50]
PLLA‐PEG‐PLLA	/	3.48–17.3	7.02 × 10^−17^–75.64 × 10^−17^	2.46 × 10^−16^–45.74 × 10^−16^	/	[117]
PLLA‐PCL‐PLLA	Strawberry	4.80–7.45	6.20 × 10^−17^–10.7 × 10^−17^	2.98 × 10^−16^–7.95 × 10^−16^	Better sensory quality; reduced water loss, molds and decay; shelf life extended to 24 days	[118]
PLDC	Okra	5.7	1.25 × 10^−17^	7.07 × 10^−16^	Reduced water loss and tissue cell damage; better overall quality	[116]
PLGC		7.1	1.63 × 10^−16^	1.17 × 10^−15^		
P (LA‐NI)	Button mushroom	3.5–4.6	/	/	Reduced cell shrinkage and damage; better flavor, firm texture, and consumer acceptability	[120]
PL‐D‐LA PL‐E‐LA PL (D25/E75) LA	Chinese bayberry	3–4 10–12 9.1	/ / /	/ / /	Reduced weight loss and respiration; higher firmness; better quality and appearance	[121]

#### 2.2.1 Plant Leaf‐Inspired Porous Membranes

**FIGURE 3:**
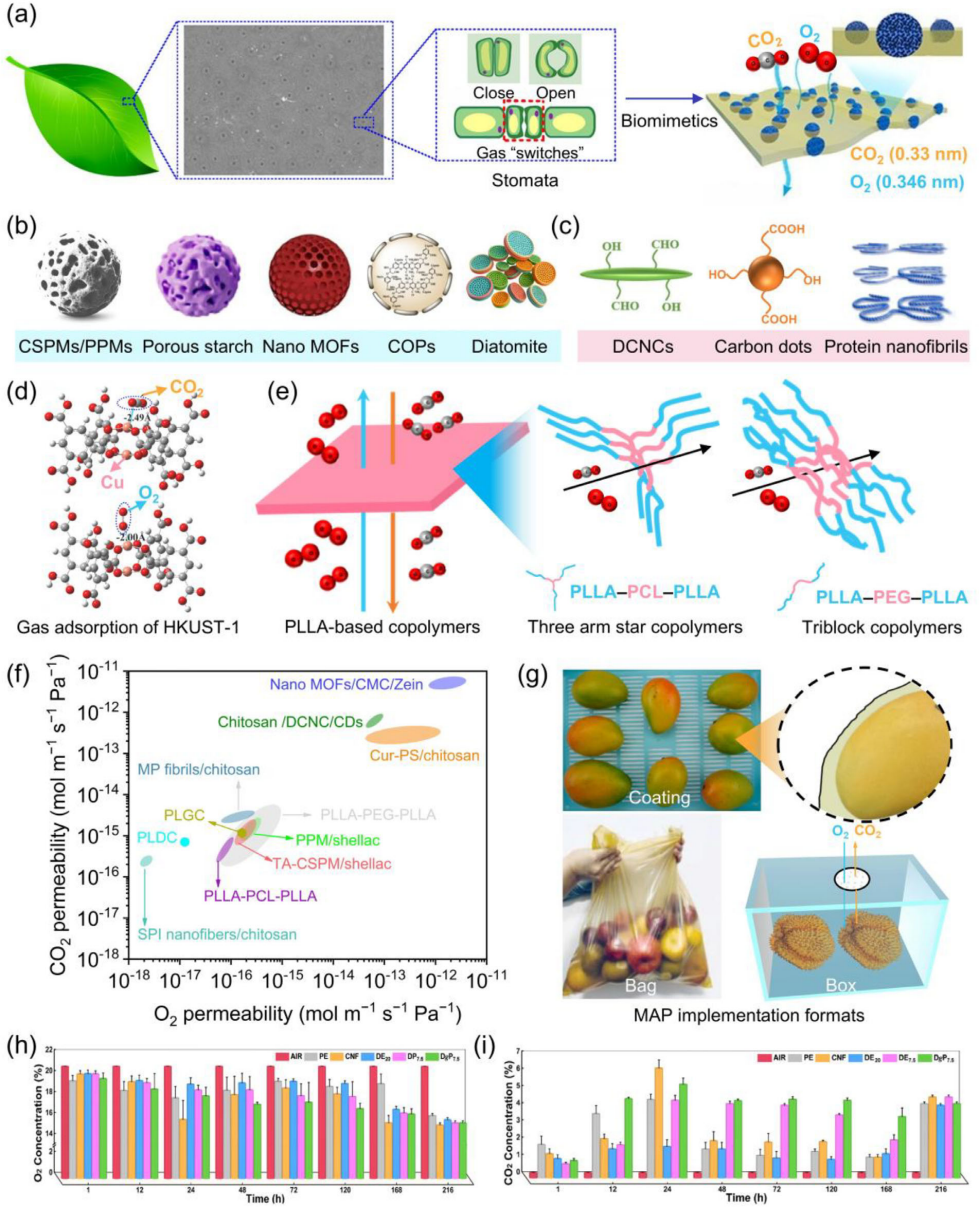
Design, fabrication, and applications of passive modified atmosphere packaging. (a) Leaf‐stomata‐inspired concept. (b) Synthetic and natural porous “switches” regulating gas permeation (covalent organic polymers—COPs). Reproduced with permission [111]. Copyright 2024, Elsevier. Reproduced with permission [112]. Copyright 2022, American Chemical Society. (c) Nonporous nanofillers introducing dense domains and tortuosity to modulate permeability/selectivity (dialdehyde cellulose nanocrystals—DCNCs). Reproduced with permission [113]. Copyright 2025, Elsevier. Reproduced with permission [114]. Copyright 2023, Royal Society of Chemistry. (d) CO_2_ and O_2_ adsorption models of HKUST‐1. Reproduced with permission [115]. Copyright 2024, Elsevier. (e) Principle of PLLA‐based copolymers for MAP films. Reproduced with permission [116]. Copyright 2022, Elsevier. Reproduced with permission [117]. Copyright 2019, Royal Society of Chemistry. (f) Comparison of CO_2_/O_2_ permeability of common modified atmosphere packaging [50, 107, 110, 111, 113, 114, 115, 116, 117, 118]. (g) Versatile packaging formats enabling controlled CO_2_/O_2_ exchange with tunable permeability/selectivity. Reproduced with permission [110]. Copyright 2021, American Chemical Society. Reproduced with permission [50]. Copyright 2024, John Wiley and Sons. (h) In‐package O_2_ concentration evolution during litchi storage. (i) In‐package CO_2_ concentration evolution during litchi storage. Reproduced with permission [103]. Copyright 2023, Elsevier.

In leaves, stomata act as dynamic gas‐exchange valves: guard‐cell turgor reversibly modulates pore aperture in response to light, internal CO_2_, humidity, and stress, balancing CO_2_ uptake with O_2_/H_2_O exchange while minimizing water loss (Figure 3a). Inspired by this switchable, selective transport, chitosan porous microspheres (CSPMs) and poly‐L‐lactic porous microspheres (PPMs) were dispersed as gas “switches” within shellac matrices to regulate gas transport in MAP films/coatings (Figure 3a,b) [110]. CSPMs prepared by phase separation (median particle ≈38 µm; median pore ≈6 nm) increased CO_2_/O_2_ selectivity from 4.7 to 8.6 (O_2_ permeability of 2.65 × 10^−16^ mol m^−1^ s^−1^ Pa^−1^; CO_2_ permeability of 21.92 × 10^−16^ mol m^−1^ s^−1^ Pa^−1^), with similar trends for PPMs. TA deposition further tuned pore size/porosity and added antioxidant/antimicrobial function, raising selectivity to 10.1. As a result, these microsphere‐loaded coatings/films prolonged shelf life in both non‐climacteric and climacteric fruits, with reduced weight loss and improved appearance (Figure 3g) [110]. During litchi storage, in‐package gases stabilized near ∼1.7% O_2_ and ∼37.9% CO_2_ after 4 days, delaying browning/rot and preserving internal quality [107]. To approach stomatal stimuli‐responsiveness, curcumin‐loaded porous starch (Cur‐PS) was incorporated into chitosan, where pH/temperature‐triggered curcumin release tuned permeability and adjusted CO_2_/O_2_ selectivity from 2.60 to 5.51, while providing antioxidant/antibacterial benefits for anti‐browning and shelf life extension [111].

To overcome pore heterogeneity and weak CO_2_ sensitivity of natural polymer‐based microspheres, nanoscale metal–organic frameworks (nanoMOFs) were integrated as molecular‐level gates (Figure 3b) [115]. Three representative nanoMOFs—ZIF‐8 (intrinsic pore aperture ≈1.076 nm), UiO‐66‐NH_2_ (≈1.410 nm), and HKUST‐1 (≈0.600 nm)—provide differentiated molecular sieving. The resulting nanoMOF/carboxymethylcellulose/zein films exhibited effective pore sizes of 0.96, 1.096, and 0.694 nm and CO_2_/O_2_ selectivities of 3.34, 5.03, and 8.65, respectively. Although O_2_ and CO_2_ are much smaller than these apertures, preferential adsorption and framework–gas interactions can retard O_2_ transport more than CO_2_ (as with HKUST‐1) (Figure 3d), effectively raising CO_2_‐over‐O_2_ selectivity and enabling low‐O_2_/high‐CO_2_ microenvironments that extended mango shelf life. Because MOF pore size and CO_2_ affinity are synthetically programmable—via metal nodes, linkers, defect engineering, or post‐synthetic modification—permeability and CO_2_/O_2_ selectivity can be tailored to specific commodities and supply‐chain scenarios. In this sense, the study provides an efficient design route for MAP films with targeted gas‐transport properties, moving beyond perforation toward composition‐tunable, function‐targeted membranes [115]. Similarly, covalent organic polymers (COPs) offer high reproducibility, solution processability, and strong interactions with quadrupolar CO_2_, and have been widely explored for CO_2_ separation [119]. Hydrogen‐bonded COPs (HCOPs) synthesized from lignin exhibited permanent porosity and high thermal stability; when combined with polyacrylonitrile membranes, the CO_2_/O_2_ selectivity increased from 6.95 to 18.46, largely due to higher CO_2_ condensability/solubility in the membrane matrix, improving preservation performance [112].

In addition to synthetic porous materials, natural diatomite (median particle diameter ≈23 µm) was incorporated into cellulose nanofibril (CNF) films to create gas‐transport channels and tune permeability/selectivity [103]. Owing to its hygroscopicity, diatomite reduced the water content of CNF matrix and hindered CO_2_ adsorption, diffusion, and dissolution, decreasing CO_2_/O_2_ selectivity from 6.32 to 0.58. To introduce CO_2_ affinity, diatomite was modified with polyethyleneimine (PEI) to provide amine‐rich sites. After PEI modification, low loadings of diatomite still decreased CO_2_ permeation (moisture‐reduction dominated), whereas higher loadings increased CO_2_ transport via amine–CO_2_ interactions; overall, CO_2_ transmission could be elevated with CO_2_/O_2_ selectivity tunable from 0.95–6.32. Notably, PEI‐modified diatomite plus polyethylene oxide (PEO) deposited onto silver interdigitated electrodes formed an in‐package CO_2_ sensor, enabling real‐time monitoring. In practice, atmosphere inside package reached equilibrium within 24 h: for green plums, O_2_ stabilized at ∼12.12% and CO_2_ at ∼8.7%; for litchis, O_2_ and CO_2_ stabilized at ∼17.5% and ∼4.5%, respectively—conditions under which freshness was effectively preserved (Figure 3h,i) [103].

#### 2.2.2 Nanoparticle‐ and Fibril‐Based Hybrid Films

Incorporating nonporous nanofillers into polymer matrices introduces dense domains and increases diffusion tortuosity, thereby modulating gas permeability and CO_2_/O_2_ selectivity. As a representative example, dialdehyde cellulose nanocrystals (DCNCs) and carbon dots (CDs) were added to chitosan films (Figure 3c) [113]. The fillers (i) promoted a denser microstructure, lowering O_2_ and CO_2_ permeability, and (ii) supplied surface hydroxyl, carboxyl, and aldehyde groups that interact preferentially with CO_2_, enhancing its solubility (thus effective transport) relative to O_2_. Chitosan films containing 3 wt.% DCNCs and 3 wt.% CDs exhibited reduced CO_2_ and O_2_ permeabilities; but O_2_ permeability declined more, increasing CO_2_/O_2_ selectivity from 8.3 to 11.2—adequate for in‐package atmosphere control for perishables. Nevertheless, the selectivity gain at such low nanofiller loadings remains modest for stringent MAP targets.

To achieve higher selectivity at practical thicknesses, high‐fraction protein nanofibrils were introduced as continuous dense domains in chitosan via thermal acidic treatment. Soybean protein isolate (SPI) unfolded, partially hydrolyzed, and reassembled into worm‐like fibrils with ultrahigh aspect ratio, dispersing homogeneously at ∼50% fibril content and yielding CO_2_/O_2_ selectivity ≈100 [114]. Here, O_2_ permeability decreased whereas CO_2_ permeability increased, attributable to the smaller kinetic diameter of CO_2_ and stronger segmental drag on O_2_ within the fibril‐rich network, together with fibril‐induced pathway differentiation that more strongly penalizes O_2_ diffusion. Using the same in situ fibrillation strategy, mung‐bean protein (MP) formed noncontinuous nanofilament domains in chitosan, achieving CO_2_/O_2_ selectivity ≈130 without compromising membrane integrity [50]. The MP–chitosan hybrids exhibited protein domains of ∼3.7 nm and a reduction of chitosan crystallite size from ∼32 nm to ∼6 nm, producing a heterogeneous microstructure with smaller effective pores. This heterogeneity—arising from polydisperse MP fibrils and their complexes with chitosan—enhanced interdomain interactions and tightened the network, lowering O_2_ diffusivity more than CO_2_ and improving mechanical robustness. Consequently, these hybrids were highly effective for modified atmosphere preservation of perishable foods (Figure 3c,g) [50].

#### 2.2.3 Polymer‐Architecture–Engineered Films

CO_2_/O_2_ selectivity can also be engineered without fillers by tailoring the molecular architecture of the film‐forming polymer. This strategy has been systematically explored for poly(L‐lactic acid) (PLLA), which is attractive for packaging owing to its biodegradability, processability, biocompatibility, and mechanical strength, yet exhibits suboptimal gas transport and CO_2_/O_2_ selectivity [121]. To address this, Yun and co‐workers synthesized PLLA‐based block/graft copolymers via ring‐opening polymerization for MAP—namely PLLA–PEG–PLLA, PLLA–PCL–PLLA, PLDC, PLGC, P(LA‐NI), and PL‐E‐LA and PL‐D‐LA [116, 117, 118, 120, 121]. By adjusting monomer type, molecular weight, feed ratio, and polymerization protocol, they programmably tuned polymer polarity, nanostructured phase separation, crystallinity, and gas affinity, thereby modulating dissolution–diffusion behavior and, in turn, permeability and CO_2_/O_2_ selectivity (Figure 3e). Relative to neat PLLA (≈3.0), the copolymers achieved higher selectivity: 17.3 for PLLA–PEG–PLLA [117]; 6–9 for PLLA–PCL–PLLA [118]; 5.7 for PLDC and 7.1 for PLGC [116]; 4.6 for P(LA‐NI) [120]; 9.1 for PL‐E‐LA and PL‐D‐LA blends [121], demonstrating composition‐level control of in‐package atmospheres. Note that polylactic acid degrades efficiently only under industrial‐composting conditions; in many natural environments degradation is slow and materials may fragment into microplastics, posing environmental concerns [122].

In MAP, CO_2_/O_2_ selectivity is governed by solubility–diffusivity trade‐offs, specific gas–matrix interactions, and the pore‐size/geometry landscape (i.e., intrinsic pore apertures and effective pore‐size distributions/tortuosity). Size‐sieving effects become significant when effective pore sizes approach gas kinetic diameters (≈3.3 Å for CO_2_ and ≈3.46 Å for O_2_), whereas differential adsorption and transport dominate when pores are appreciably larger. Guided by structure–function principles, three complementary strategies have emerged: (i) biomimetic pore design (porous microspheres, MOFs, diatomite) leveraging pore size/geometry and CO_2_‐preferring adsorption; (ii) nanoparticle/nanofibril hybrids that introduce dense domains and higher tortuosity, asymmetrically suppressing O_2_ diffusion while maintaining sufficient CO_2_ flux; and (iii) polymer‐molecular engineering (no fillers) that tunes polarity, crystallinity, and phase morphology to enhance selectivity at the chain‐architecture level (Figure 3f). Despite substantial progress, translation will depend on MAP systems that pair high selectivity with food‐contact safety, mechanical robustness, UV/abrasion/moisture stability, and scalable green fabrication, validated by standardized testing and in‐package monitoring under realistic supply‐chain conditions.

### 2.3 Active Packaging

Beyond respiration and senescence, microbial contamination and nutrient oxidation are major drivers of spoilage from postharvest handling through retail and consumption [10, 123]. Owing to high water and nutrient contents, fresh produce is susceptible to microbial infection, leading to rot and sensory deterioration [9]. Exposure to air further promotes oxidation of polyphenols, proteins, amino acids, vitamins, unsaturated fatty acids, and lipids, accelerating discoloration, nutrient loss, and off‐flavors in fruits, vegetables, and meat [10, 11, 12]. Conventional countermeasures—chemical preservatives, physical treatments, and biological control—can extend shelf life [124], but their adoption is constrained by concerns over sensory quality, human health, and environmental impacts. For instance, synthetic fungicides may cause ion accumulation, membrane disruption, and inhibition of essential metabolites in vivo [19]. Physical methods (heating, irradiation, hypobaric treatment) are generally safe yet transient and can alter appearance and internal nutritional quality [125, 126]. Moreover, antibiotic overuse is associated with ∼700 000 deaths annually and could cause up to 10 million deaths by 2050 [127].

**FIGURE 4:**
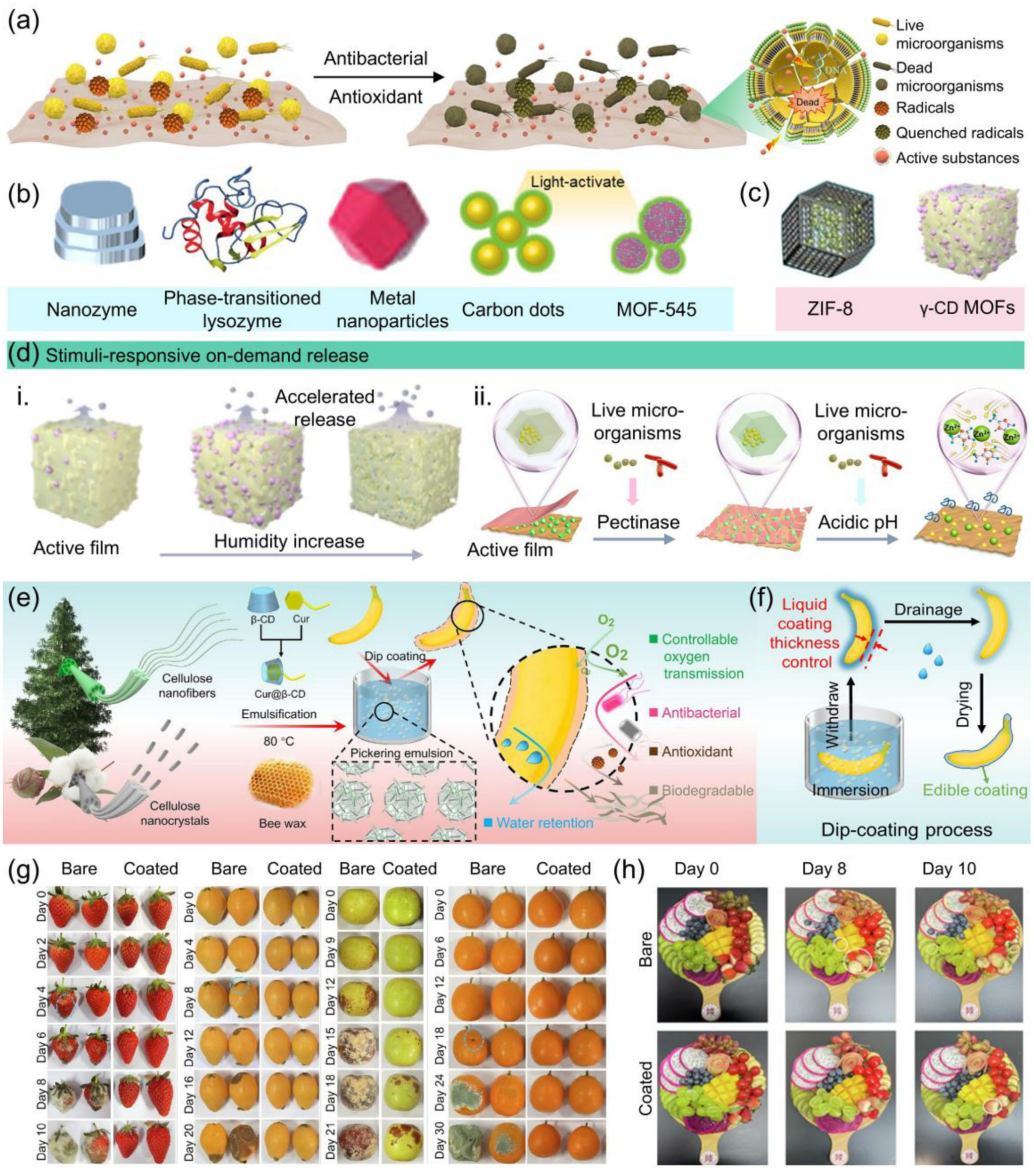
Design, fabrication, and applications of active packaging. (a) Antibacterial and antioxidant mechanisms of bioactive‐loaded films. Reproduced with permission [149]. Copyright 2024, Elsevier. (b) Emerging bioactives and technologies for active packaging. Reproduced with permission [146]. Copyright 2024, Elsevier. Reproduced with permission [171]. Copyright 2023, Springer Nature. Reproduced with permission [148]. Copyright 2025, Elsevier. Reproduced with permission [163]. Copyright 2023, Elsevier. Reproduced with permission [172]. Copyright 2024, Elsevier. (c) Advanced carriers for stimuli‐triggered release. Reproduced with permission [170]. Copyright 2024, Elsevier. (d) Triggered‐release mechanisms in active packaging systems. Reproduced with permission [173]. Copyright 2025, Elsevier. Reproduced with permission [174]. Copyright 2024, Elsevier. (e) Fabrication of multi‐functional bio‐based edible coatings. (f) Schematic depicting the regulation of coating thickness during the dip coating process. Reproduced with permission [45]. Copyright 2025, Elsevier. (g) Photographs of bare fruits and coated fruits with amyloid‐like protein coatings after different storage times. (h) The influence of the amyloid‐like protein treatment on the visual quality of fresh‐cut fruit over a storage duration ranging from 2 to 10 days. Reproduced with permission [48]. Copyright 2025, Springer Nature.

Active packaging—embedding antibacterial and/or antioxidant bioactives into films/coatings—can extend shelf life with minimal compromise to quality or safety (Figure 4a,b and Table 3) [128]. Direct incorporation of essential oils (EOs) (e.g., lemon [129], thyme [130]), and polyphenols [45, 131, 132, 133] via emulsification has yielded promising outcomes [134, 135]. However, many agents are lipophilic, volatile, and light/thermally unstable, leading to poor aqueous solubility, uneven distribution, burst release, and low bioavailability. To address this, diverse encapsulation and release‐control strategies—particle encapsulation (e.g., liposomes, cyclodextrin, natural‐polymer microcapsules and NPs) [129, 136, 137], electrospun carriers [138, 139], nanoemulsions [140], multilayer packaging [52], and Pickering emulsions [141, 142]—have been developed. As these have been extensively reviewed elsewhere [143, 144, 145], here we emphasize emerging actives/technologies (Figure 4b), advanced carriers (Figure 4c), and stimuli‐responsive release (Figure 4d) not covered previously.

**TABLE 3:** Active packaging for preserving perishable foods.

Active substance	Carrier	Matrices	Encapsulation method	Food	Antibacterial spectrum	Free radical scavenging	Release duration (conditions)	Preservation effect	Refs.
Cu‐BSA nanozymes	Chitosan NPs	Carrageenan	Chemical adsorption	Fresh‐cut apples; fig	*E. coli*; *S. aureus*	>80% (·O_2_^−^)	/	Reduced weight loss, microbial contamination, oxidative browning; higher firmness	[146]
BSA‐mediated crystalline Mn_3_O_4_ (nanozyme)	CNTs	Chitosan	Bifurcated methodology	Kumquat	*E. coli*; *S. aureus*; *Penicillium italicum*; *Penicillium expansum*	/	/	Reduced weight loss, bacteria and mold; shelf life extended to 24 days; higher firmness	[147]
Se NPs	/	Starch/CMC	/	Litchi	*E. coli*; *S. aureus*	10%–90% (DPPH)	/	Reduced dehydration, nutrient loss, microbial growth; better quality; prolonged storage	[149]
Theanine/malic acid carbon dots	/	/	/	Salmon	*Pseudomonas fragi*	/	/	Reduced bacterial growth; blocked siderophore Fe^3+^ uptake; shelf life extended by 3–4 days	[150]
Lysozyme	/	Sodium alginate/CNCs	/	Winter jujube; strawberry; kumquat; loquat; nectarine; mango; kiwi; *Ficus carica*; wolfberry; cherry tomato; banana	*E. coli*; *S. aureus*; *Mucor*; *Fusarium*; *Meyerozyma*; *Gluconobacter*; *Pantoea*	71% (DPPH); 100% (ABTS)	/	Reduced browning, decay, microbial damage; better nutrition, flavor, firmness; shelf life extended by 2 – 5‐fold	[48]
Turmeric CDs	/	Chitosan	/	Pork	*E. coli*; *S. aureus*	/	/	Reduced water loss, microbial growth, spoilage; better appearance and quality	[164]
Geraniol	MOF‐545	PVA/CMC	Liquid‐phase adsorption	Cherry tomato	*E. coli*; *S. aureus*	/	/	Reduced shrinkage and water loss; better appearance; higher firmness; shelf life extended to 12 days	[131]
Carvacrol	PCN‐224	PCL/chitosan	Physical adsorption	Fresh‐cut apple	*E. coli*; *S. aureus*	/	>150 h (PBS)	Reduced reddish‐brown spots and browning; higher firmness	[172]
Quercetin	Chitosan NPs	Chitosan	Chemical adsorption	/	*E. coli*; *L. monocytogenes*	47.27% (ABTS); 74.36% (DPPH)	1 h (food‐simulant)	/	[166]
Curcumin	β‐cyclodextrin	CNFs/CNCs	Host–guest interaction	Banana; strawberry; grape; mango; avocado	*E. coli*; *S. aureus*	84.3% (DPPH)	192 h (PBS)	Lower deterioration and senescence; higher firmness; reduced respiration, dehydration, water loss; better quality and freshness	[45]
Lemon essential oil	Porous vermiculite	Konjac glucomannan‐grafted‐PLA/PVA	Physical loading and electrospinning	Chilled pork	*E. coli*	/	76 h (25°C ethanol)	Reduced microorganisms and reproduction; shelf life extended by 3 days	[129]
Limonene	γ‐CD MOFs	PCL nanofibers	Impregnation	Fresh‐cut apple	*E. coli*; *S. aureus*	/	576 h (20°C; 40% RH)	Reduced bacterial growth; better quality	[138]
Curcumin	ZIF‐8	Chitosan	Solvent evaporation	Citrus	*E. coli*; *S. aureus*	79.5% (ABTS) 62.2% (DPPH)	144 h (PBS)	Reduced mold; improved appearance and quality; shelf life extended to >34 days	[170]
Thymol	γ‐CD MOFs	Zein/pectin/sodium alginate	Gas phase diffusion	Strawberry; *Agaricus bisporus*	*E. coli*; *S. aureus*; *B. cinerea*	/	>336 h (23%/58%/85% RH)	Reduced weight loss, shrinkage, microbial growth, and reproduction	[173]
Cinnamaldehyde	Chitosan	/	Imidization	Broccoli; strawberry	*E. coli*; *S. aureus*	11% (ABTS); 41% (DPPH)	120 h (5%–20% CO_2_ humid headspace)	Reduced rotting, respiration, whitening, and weight loss; shelf life extended by 6 days	[175]
Carvacrol	ZIF‐8	CNFs/pectin	One‐pot synthesis	Raspberry; mango	*E. coli*; *S. aureus*; *A. niger*	/	168 h (PBS)	Reduced fungal disease and mycelium growth; better quality	[174]
Thymol	ZIF‐8	κ‐carrangeenan/Zein	Solvent impregnation	Blueberry	*E. coli*; *S. aureus*	61.49% (ABTS) 75.60% (DPPH)	36 h (PBS)	Reduced weight loss, color changes, and wrinkling; higher firmness	[130]
Oregano EOs	Gelatin/carrageenan microcapsules	/	Complex coacervation	Cherry tomato	*B. cinerea*	/	>80 h (5 mL 60 % PBS and 40 % ethanol)	Better firmness, quality, glossiness; reduced respiration and post‐ripening	[137]
Curcumin	Protein‐polysaccharide hybrid NPs	BC	Homogenizing; pickering emulsion	Basa fish	*E. coli*; *S. aureus*	74% (ABTS) 27% (DPPH)	3 h (50 % ethanol)	Reduced odor, stickiness, and weight loss	[141]
Thymol; carvacrol	UiO‐66‐NH_2_	Chitosan	Physical adsorption	/	*E. coli*; *S. aureus*; *P. citrinum*	/	200 h (controlled humidity, desiccators)	/	[169]
Piceid; resveratrol	/	/	/	Cherry; peach	*E.coil*; *S. aureus*; *B.subtilis*; yeast	75.73% (ABTS) 38% (DPPH)	/	Reduced corruption by 36.81%; reduced microbial reproduction, browning, water and nutrient loss	[176]
Chiral boron dipyrromethene nano‐formulations	/	Chitosan	/	Cherry; strawberry	*S. aureus*	/	/	Reduced mildew, water and nutrient loss; better sensory and nutritional quality	[159]

#### 2.3.1 Nanoactive Antibacterial/Antioxidant Systems

Beyond natural EOs and polyphenols, synthetic or engineered actives with higher durability—such as nanozymes [146, 147], metal NPs [148, 149], carbon dots [150], and phase‐transitioned lysozyme (PTL) [48]—have been employed in active packaging (Figure 4b). Compared with natural enzymes, nanozymes are easier to synthesize, cost‐effective, readily sourced, and offer tunable oxidase‐, peroxidase‐, and superoxide‐dismutase–like activities. For example, Mn^2+^ was immobilized on carbon nanotubes (CNTs) via electrostatic and coordination interactions; subsequent bovine serum albumin (BSA)–mediated wet‐chemical nucleation yielded crystalline Mn_3_O_4_ with strong oxidase‐like activity and photothermal responsiveness [147]. Complexation with mildly acidic chitosan autonomously activated the nanozyme, delivering potent antibacterial/antifungal performance; near‐infrared heating further damaged bacterial cell walls, yielding synergistic killing and significantly prolonging the shelf life of kumquats. Similarly, BSA‐chelated Cu^2+^ mimicking superoxide dismutase exhibited enzymatic activity, antibacterial function, and biocompatibility; carrageenan films containing this nanozyme suppressed enzymatic browning of fresh‐cut apples and protected figs from microbial contamination [146].

Silver and zinc NPs are widely incorporated into biopolymers to inhibit microbial growth [148, 151]. Selenium NPs have gained attention for strong antioxidant and antibacterial activity; effective use hinges on preventing aggregation to preserve bioactivity and bioavailability. Polyphenols and polysaccharides stabilize selenium NPs, improving dispersion and function, thereby suppressing microbial growth and preserving freshness [149]. Compared with inorganic metal–based agents, organic nanomaterials such as CDs are generally less toxic and more environmentally friendly. CDs exhibit antibacterial effects via cell‐surface damage, reactive oxygen species (ROS) generation, and biosynthesis inhibition. For instance, theanine/malic‐acid–derived CDs competitively chelated Fe^3+^ (via Fe–O bonding) with bacterial siderophores, causing iron deprivation and bacterial death; a simple soaking treatment extended salmon shelf life by 3–4 days [150]. PTL also shows strong antibacterial activity: beyond peptidoglycan hydrolysis, increased positive charge density and exposed hydrophobic residues after phase transition enhance membrane interactions. A spray/immersion treatment formed a robust lysozyme‐based adhesive on the epidermal wax layer of various fruits, extending the shelf life of 17 families of perishables by 2–5‐fold (Figure 4g,h) [48].

#### 2.3.2 Light‐Driven Antimicrobial Strategies and Chemo–Optical Coupling

Light‐driven antimicrobial packaging converts light into antimicrobial action via photodynamic inactivation, photocatalysis, and photothermal treatment. Photodynamic inactivation uses photosensitizers to generate ROS (notably singlet oxygen) for microbial killing [152, 153], whereas photocatalysis typically relies on photoexcited charge carriers in semiconductors to drive interfacial redox reactions and produce ROS such as ∙OH and ∙O_2_^−^ [154]. In contrast, photothermal systems dissipate light into heat, directly stressing microbes and, importantly for packaging, accelerating transport to enable triggered dosing [155, 156]. These light‐driven approaches offer the advantage of non‐contact operation and can be engineered with food‐grade materials. However, their antimicrobial efficacy is highly dependent on light dose and the localized levels of ROS or temperature, which must be carefully controlled to avoid sensory degradation of food products [157, 158, 159]. Notably, these optical mechanisms can also act synergistically; for instance, photothermal heating has been shown to amplify both photodynamic [160] and photocatalytic [161] ROS‐mediated inactivation.

A more versatile strategy involves chemo–optical coupling, where chemical antimicrobials are integrated with light‐driven modules. In such systems, the chemical agents provide continuous antimicrobial stress, while the optical component offers on‐demand amplification via localized ROS or heat. A classic example is chemical–photodynamic integration, where natural compounds act as photosensitizers—curcumin being a canonical case [162]. For instance, hydrothermally synthesized carbon quantum dots derived from turmeric generated ROS under blue light (Figure 4b), leading to membrane disruption and macromolecule leakage; when incorporated into chitosan films for pork preservation, this system effectively slowed microbial spoilage and maintained meat quality [163, 164]. Porphyrin‐based MOFs (e.g., MOF‐545) further exemplify this synergy by combining high loading capacities with intrinsic photodynamic activity (Figure 4c). When loaded with geraniol, MOF‐545 delivered combined photodynamic and chemical antibacterial effects against *E. coli* and *S. aureus*, extending the shelf life of cherry tomatoes beyond 12 days [131]. Photothermal coupling is particularly attractive for active packaging, as localized heating both stresses microbes and modulates the release of co‐incorporated actives. For example, CNT‐supported Mn_3_O_4_–CS films combined nanozyme‐like oxidative activity with photothermal stress, achieving rapid broad‐spectrum inactivation and a 2.3‐fold extension in shelf life [147]. Multichannel designs can further integrate chemical and interfacial effects: in ZIF‐8–TA/black phosphorus quantum dot (BPQD) systems, photothermal heating was proposed to increase membrane permeability, while BPQDs generated ROS and polyphenol‐derived surface charges cooperatively disrupted cells, enabling effective food preservation [165]. Beyond direct inactivation, photothermal effects can also gate the transport of antimicrobials. In a chitosan@citral/zein/polydopamine system, NIR activation increased citral release and headspace enrichment, consistent with a photothermal‐controlled delivery mechanism [156].

#### 2.3.3 Advanced Carriers and Post‐Synthetic Tailoring for Sustained/On‐Demand Release

Although natural phytochemicals are prone to volatilization and degradation, their synergistic antimicrobial and antioxidant functions remain attractive. Recent work therefore emphasizes encapsulation and sustained/triggered release to enhance stability and bioavailability. Representative systems include flavonoids [166], curcumin [45], and lemon essential oil [129], encapsulated in chitosan NPs, β‐cyclodextrin, and nanoclay, respectively, yielding films/coatings with strong antibacterial performance and radical‐scavenging activity (e.g., DPPH, ABTS) (Figure 4e,f). To ensure uniform dispersion of inclusion complexes, electrospinning [129, 167, 168] and Pickering emulsions [45, 141] are commonly employed. MOFs can also encapsulate EOs to improve stability and enable sustained release [169]. To further raise loading and tailor release, functional MOFs/MOF derivatives have been created via composition and structure design (Figure 4c). For example, nano γ‐cyclodextrin MOFs (γ‐CD MOFs) prepared ultrasonically combined the advantages of γ‐cyclodextrin and MOFs, increasing limonene loading to ∼170 mg g^−1^ [138]. Post‐synthetic modification is also effective: mesoporous ZnO‐doped hollow carbon nanocages, obtained by TA etching and high‐temperature annealing of ZIF‐8, sustained curcumin release via hierarchical tortuosity and steric hindrance, producing composite films with enhanced bactericidal and antioxidant performance and extending citrus shelf life to >34 days [170].

Building on these active agents and carriers, the next step is stimuli‐responsive systems that deliver on‐demand, spatiotemporally controlled release. Triggers arise from physical or biotic cues in the headspace or at the food–package interface, including humidity, pH, temperature, light, enzymes, and produce respiration (CO_2_/H_2_O) (Figure 4d). Humidity‐driven release can be realized with thymol‐loaded γ‐CD MOFs: water vapor penetrates the pore network and competes for host–guest interactions, weakening hydrogen bonding/coordination within the framework and displacing thymol from binding sites; at sufficiently high humidity, partial framework disassembly further accelerates payload liberation. Thus, ambient humidity functions as a reversible switch that modulates thymol delivery into the package headspace [173]. To exploit the acidifying microenvironment from commodity respiration, Liu et al. fabricated a Schiff‐base emitter by imidizing chitosan with cinnamaldehyde (CS–Cin). The imine (C═N) linkage is acid‐labile; CO_2_ and moisture from respiration form a mildly acidic condensate, cleaving the imine and releasing cinnamaldehyde. As a container‐fixed, non‐contact pad, the emitter suppressed cytomembrane peroxidation and microbial growth, extending the shelf life of broccoli and strawberries by ∼300% [175]. For greater specificity, a dual‐trigger (pH/enzymatic) gate can be employed. Carvacrol is encapsulated in ZIF‐8 nanocarriers grown in situ on CNF films, and a pectin overlayer—electrostatically bound—acts as a gatekeeper (Figure 4c). Acidic conditions associated with spoilage protonate imidazolate linkers and dissolve ZIF‐8 (Zn–imidazolate) nodes, while fungal pectinase depolymerizes the pectin shell; either cue (especially both) opens diffusion pathways, converting environmental signals into on‐demand carvacrol release. This dual‐gated design improves selectivity and timing of dosing, achieving effective preservation in fungus‐challenged fruit pads [174].

The antibacterial/antioxidant efficacy of active packaging derives from the chemistry and stability of loaded actives and the trigger–release architecture. This is particularly critical for perishables, which are prone to microbial spoilage and oxidative deterioration, and for bio‐based films that, if unprotected, undergo oxidation and can inadvertently serve as nutrient media. Engineered actives (nanozymes, metal NPs, CDs) provide stimulus‐resilient functionality, while porous carriers (e.g., γ‐CD MOFs, MOF‐545, ZIF‐8) afford high loading and mechanism‐tailored release. Smart, cue‐responsive designs improve dose timing and localization, maximizing efficacy at lower additive loadings. However, dispersed phases and high filler fractions can compromise mechanical integrity, sealability, and optical clarity. Practical translation therefore requires compatibilization strategies, filler‐efficient designs, and multilayer/Janus architectures that preserve package function, together with scalable, cost‐effective manufacturing and food‐contact safety substantiated by standardized migration and toxicology protocols.

This emphasis on safety follows from a central trade‐off: the same actives and carriers that deliver preservation benefits can also introduce exposure risks, and these risks are not fixed material constants but depend on dose, release kinetics, food matrix, and use conditions. Plant essential oils, while widely used for flavoring, can exhibit pro‐oxidant or genotoxic effects at elevated concentrations, and migration is generally enhanced at higher temperature and in lipophilic (fatty) matrices [177, 178]. Metal‐based nanoparticles may pose hazards through ion release and oxidative stress, with risk often amplified under acidic conditions, elevated temperature, and UV exposure; their small size can also facilitate cellular uptake and, in some cases, translocation across biological barriers [179]. Carbon dots show size‐ and surface‐chemistry‐dependent cytotoxicity; ultrasmall (<6 nm) fractions may cross the blood‐brain barrier, and photodegradation under light can generate potentially harmful byproducts [180, 181]. MOFs add stability‐driven uncertainty, as pH and humidity can trigger partial framework degradation and accelerate the release of metal nodes or organic linkers, which may challenge specific migration limits in acidic simulants [182]. Nanozymes introduce a dual concern—catalytic reactive‐oxygen generation and potential ion release—yet their migration and long‐term oral safety remain insufficiently characterized under standardized food‐contact conditions [183]. Collectively, these observations underscore that dose control is decisive: each class has an operating window that reconciles efficacy with safety, whereas over‐formulation or uncontrolled migration can shift systems into adverse regimes. Establishing this window requires harmonized protocols that quantify migration (including chemical and, where relevant, particulate/ionic forms) and evaluate tiered toxicology across realistic time–temperature and food‐simulant conditions. Regulatory compliance and consumer trust further hinge on demonstrating adherence to migration limits and communicating safety margins transparently. A detailed safety framework is provided in Section 3.2.

### 2.4 Intelligent Packaging

**FIGURE 5:**
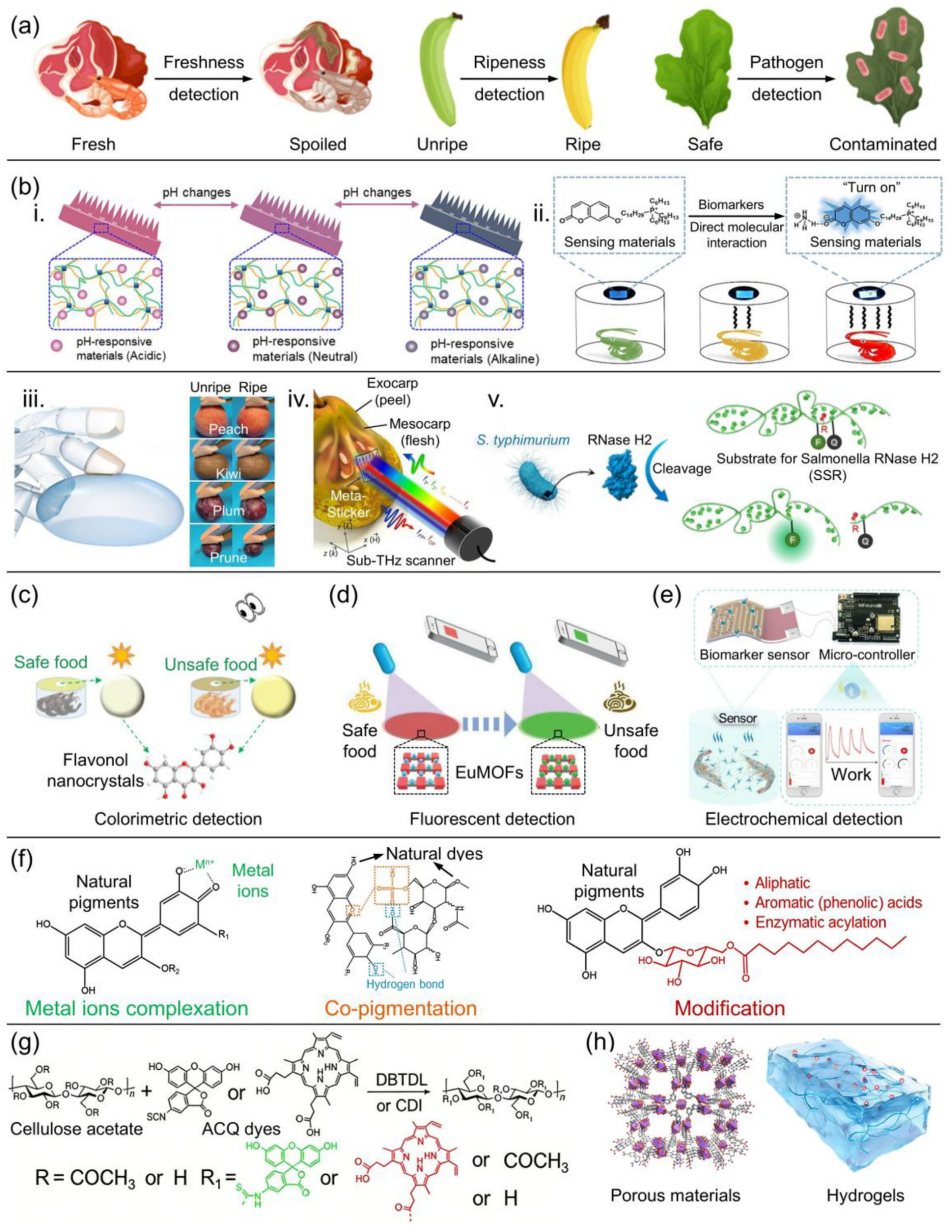
Design principles and engineering strategies of highly sensitive intelligent packaging platforms with long‐term stability and rapid response. (a) Intelligent packaging for food‐quality monitoring. (b) Signal‐generation mechanisms: (i) pH detection via protonation/deprotonation processes for freshness monitoring. Reproduced with permission [248]. Copyright 2024, Elsevier. (ii) Direct interactions between biomarkers and sensing platforms. Reproduced with permission [220]. Copyright 2024, Elsevier. (iii) Elastic modulus and surface shape sensing by 3DAE‐Skin for ripeness assessment. Reproduced with permission [234]. Copyright 2024, The American Association for the Advancement of Science. (iv) Sub‐THz metamaterial sticker for refractive‐index‐based ripeness evaluation. Reproduced with permission [235]. Copyright 2025, Springer Nature. (v) Synthetic nucleic‐acid probes with fluorophore/quencher flanking for pathogen detection. Reproduced with permission [245]. Copyright 2023, John Wiley and Sons. (c–e) Multimodal strategies for food quality monitoring: colorimetric, fluorescent, and electrochemical sensing. Reproduced with permission [216]. Copyright 2023, Elsevier. Reproduced with permission [64]. Copyright 2021, John Wiley and Sons. Reproduced with permission [224]. Copyright 2024, Elsevier. (f) Chemical and physical stabilization strategies for natural dyes to enhance stability and sensitivity. (g) Covalent immobilization of ACQ dyes onto polymer frameworks to suppresses self‐quenching. Reproduced with permission [213]. Copyright 2019, Springer Nature. (h) Porous materials and hydrogels for kinetics acceleration and sensitivity enhancement. Reproduced with permission [219]. Copyright 2022, Elsevier. Reproduced with permission [55]. Copyright 2024, John Wiley and Sons.

Food is not food unless it is safe [184, 185, 186]. Building on the motivation outlined in the Introduction, we shift from centralized, sample‐destructive laboratory testing to in‐package sensing that enables non‐destructive, on‐site, and real‐time monitoring across the supply chain [187, 188, 189]. Intelligent packaging embeds sensing, logging, communication, and data analytics directly into the package, supporting freshness (TVB‐N, BAs, H_2_S/CO_2_, VOCs), ripeness (ethylene/VOCs, firmness, refractive‐index proxies) [190], pathogen surveillance [7], anti‐counterfeiting [191, 192, 193], environmental monitoring [194, 195], antibiotic‐residue screening [196, 197, 198], and endogenous toxic‐ion detection [199, 200]. While prior reviews summarize broad progress [40, 201, 202, 203], actionable design guidance that connects biomarker selection, transduction mechanisms, materials/fabrication choices, and readout pipelines (e.g., smartphone/RFID/NFC with AI‐assisted calibration) remains limited. Therefore, focusing on freshness, ripeness, and pathogen monitoring (Figure 5a and Table 4), this section synthesizes practical design rules to guide the development of stable, sensitive, and accurate intelligent packaging systems.

**TABLE 4:** Intelligent packaging for perishable foods.

Quality	Biomarker	Sensing material	Matrices	Format	Readout	Food	Response time	Detection range/LOD	Accuracy	Refs.
Freshness	TVB‐N; BAs	Synthetic/natural pigments	CA/PDMS	Array	Colorimetric	Chicken; fish; Beef	/	0.05–100 ppm (NH_3_ and BAs); LOD < 0.05 ppm	98.5%	[62]
	TVB‐N	Cu‐MOF	Starch/PVA	Film	Colorimetric	Shrimp	/	LOD 0.8 mM (NH_3_)	/	[218]
	TVB‐N; BAs	Metal‐polyphenol	PTFE	Array	Colorimetric	Beef; chicken; fish; shrimp	A few seconds	2–1000 ppm; LOD 1 ppm (NH_3_), 3 ppm (DMA), 2 ppm (TMA), 0.3 ppm (Put), 0.7 (Cad)	99.83%	[65]
	CO_2_	Bromothymol blue	CMC‐Na/carrageenan	Film	Colorimetric	Fresh‐cut papaya	/	0%–10%	/	[294]
	VOCs	Synthetic pigments	Xanthan/tragacanth gum	Hydrogel	Colorimetric	Bananas; apples; pears	/	0.117–3.92 ppm (acetaldehyde), 0.153–5.202 ppm (propionaldehyde), 0.088–5.032 ppm (acetone); LOD 0.11 ppm (acetaldehyde), 0.14 ppm (propionaldehyde), 0.07 ppm (acetone)	/	[295]
	BAs	Methyl red@Eu MOFs	CMC‐Na agarose	Hydrogel	Fluorescent	/	25 min	0.1–250 µM (His); LOD: 0.1µM	/	[280]
	BAs	Diphenyl acridine materials	Diphenyl acridine‐based materials/polymethyl methacrylate	Film	Fluorescent	Shrimp	15 s	1.3–1330 ppm (Cad); LOD 1.3 ppm (Cad)	/	[206]
	BAs	UCNPs/curcumin	PVA	Hydrogel	Fluorescent	Fish; shrimp; pork	45 s	0–1000 µm (BAs); LOD 2.73 µM (BAs)	/	[281]
	VOCs	Natural pigments	Filter paper	Array	Fluorescent	Yardlong beans; spinach; sweet corn	/	/	96.21%	[209]
	BAs	Eu@HOF‐12	Agarose	Hydrogel	Colorimetric; fluorescent	Shrimp	0.6 s (Put)	1–2000 µM (Put), 1–1000 µM (Cad), 1–1000 µM (His); LOD 5.3 µM(Put), 3.4 µM (Cad), 2.3 µM (His)	>99.9%	[67]
	BAs	Naphthalene‐based fluorophore	Filter paper	Filter paper	Colorimetric; fluorescent	Pork; chicken; shrimp	30 s	0–200 ppm; LOD 2.69 ppm (Put), 6.11 ppm (Cad)	/	[221]
	TVB‐N; H_2_S	Transistor	Si substrates	Array	Electrochemical	Chicken tenderloin	/	0.01–5 ppm (NH_3_/H_2_S); LOD 0.01 ppm	/	[287]
	TVB‐N	PEDOT:PSS/FeCl_3_	PET	Film	Electrochemical	Shrimp	/	1–400 ppm (NH_3_); LOD 0.23 ppm (NH_3_)	/	[224]
	TVB‐N	Ga‐Cu Dual single‐atom nanozymes	2D layered double hydroxide	Chip	Electrochemical	Pork; beef; lamb; chicken	4 s	0.05–0.4 mM (NH_3_); LOD 5.9 µM (NH_3_)	/	[225]
Ripeness	Ethylene	Pd NP‐functionalized Ti_3_C_2_T_x_	Polyimide/Cu	Flexible electronics	Electrochemical	Kiwifruit	31.8 s	2–100 ppm (ethylene)	97.5%	[233]
	VOCs	Synthetic dyes	CelluMOFs	Array	Colorimetric	Mango; peach; banana	/	8–1500 ppm (*trans*‐2‐hexenal); LOD 15–150 ppm for various VOCs	99.09%	[68]
	Refractive index	Metallic meta‐atoms	Glossy paper	Sticker	Refractive index	Persimmon; pear; mango	*/*	/	/	[235]
	TOC	psaFeN	Sodium alginate	Hydrogel	Colorimetric	Strawberry; strawberry tomato; kiwifruit; passion fruit	/	3–27 µmol L^−1^ (ferulic acid); LOD 0.31 µmol L^−1^ (ferulic acid)	/	[237]
Pathogen	*Y. enterocolitica*	Colloidal gold‐labeled antibody	Nitrocellulose membranes	Strip	Colorimetric	Milk	Within 10 min	10^2^–10^7^ CFU mL^−1^ (CICC 21669), 10–10^7^ CFU mL^−1^ (CICC 21681), 10–10^7^ CFU mL^−1^ (CICC 21567); LOD 1.3 × 10^3^ CFU mL^−1^ (CICC 21669), 3 × 10^2^ CFU mL^−1^ (CICC 21681), 8 × 10^2^ CFU mL^−1^ (CICC 21567) LOD 10^3^ CFU mL^−1^ (E. coli)	/	[249]
	Protein	DNAzyme probe	Cyclo‐olefin polymer film	Assay	Fluorescent	Beef; sliced apples	*/*	/	/	[252]
	RNAzymes	Nucleic acid probe	Polyethylene	Packaging tray	Fluorescent	Whole chicken	/	10^4^–10^8^ CFU mL^−1^ (*S*. Typhimurium); LOD 10^3^ CFU mL^−1^ (*S. typhimurium*)	/	[54]
	VOCs	Dyes	Filter paper	Array	Colorimetric	Fresh‐cut lettuce	/	LOD 10^3^–10^4^ CFU mL^−1^ (*E. coli*)	91%–95%	[23]

#### 2.4.1 Freshness Sensing: Biomarkers, Mechanisms, and Readouts

Spoilage generates characteristic headspace fingerprints—total volatile basic nitrogen (TVB‐N), BAs, H_2_S, CO_2_, and broader VOCs. TVB‐N—such as NH_3_, dimethylamine (DMA), and trimethylamine (TMA)—primarily arises from enzymatic/microbial protein degradation and drives sharp off‐odors and mucosal irritation, compromising food quality and acceptability [204]. BAs such as histamine (His), putrescine (Put), and cadaverine (Cad) form via decarboxylation of amino acids or transamination/amination of aldehydes and ketones [205], causing physiological symptoms including headaches, hypertension, and vomiting [206]. H_2_S, generated by sulfur‐containing amino acid catabolism or microbial sulfate reduction, is strongly malodorous and cytotoxic at elevated levels [207]. CO_2_ and VOCs originate from endogenous and microbial metabolism; their accumulation signals spoilage and degrades sensory quality. These species shift headspace pH: TVB‐N/BAs generally alkalize [40], whereas CO_2_/H_2_S acidify [208]; VOC effects depend on food matrix (tending alkaline in protein‐rich produce but acidic in sugar‐rich produce) [209]. Accordingly, pH‐responsive optical indicators—natural/synthetic pigments [62, 208, 209, 210, 211, 212], fluorescein derivatives [64, 213, 214], covalent organic frameworks (COFs) [56, 69], coumarins [215], flavonol nanocrystals [216], and nanozymes [217]—translate protonation/deprotonation into visible color or fluorescence changes for rapid freshness readouts (Figure 5b).

Beyond indirect pH sensing, materials can interact directly with TVB‐N/BAs to generate signals via photoinduced electron transfer (PET), intramolecular charge transfer (ICT), fluorescence resonance energy transfer (FRET)/antenna effects, hydrogen bonding, metal coordination, or covalent capture, producing color/fluorescence shifts or electrical responses (Figure 5b). Representative optical platforms include MOFs [218], fluorescein probes [219], ionic liquids [220], metal–polyphenol networks [65], hydrogen‐bonded organic frameworks (HOFs) [67], and naphthalene/diphenylacridine fluorophores [206, 221], electrochemical implementations use nanostructured polyaniline [222], metalloporphyrins [223], conjugated polymers [224], poly(styrene‐co‐maleic anhydride) [63], and dual single‐atom nanozymes [225] to transduce adsorption/reaction events into resistance/capacitance changes. For H_2_S, optical probes (coumarin/naphthalimide reagents, Ag NP systems) rely on redox, nucleophilic addition, or metal‐displacement chemistry [226, 227, 228], while metal‐oxide semiconductors enable electrochemical H_2_S readout via surface redox/charge‐transfer modulation [229]. VOCs can also be resolved using colorimetric sensor arrays (CSAs); COF‐on‐MOF heterostructures functionalized with metalloporphyrins exploit π‐conjugation and metal–ligand coordination to produce analyte‐specific chromatic shifts for trace aldehydes and esters relevant to freshness [230].

#### 2.4.2 Ripeness Assessment: Biomarkers, Mechanisms, and Readouts

Ripeness control minimizes transport damage and loss and ensures food safety, as many fruits are harvested before full physiological maturity [68, 231]. Key ripeness biomarkers include ethylene/VOCs, firmness, and refractive index. Climacteric fruits show characteristic peaks in respiration and ethylene biosynthesis accompanied by evolving VOC profiles (alcohols, esters, aldehydes, ketones) [68, 232]. Although external shape changes little during ripening, firmness declines as cell‐wall architectures disassemble; in parallel, starch‐to‐sugar conversion increases soluble solids and thus tissue refractive index (sugars have a higher refractive index than starch). Ethylene labels typically use electrochemical transduction on conductive substrates functionalized with adsorption/recognition sites. Upon ethylene adsorption, local electronic structure changes, producing measurable resistance/conductivity variations. Demonstrated platforms include Cu(I) complex/CNT composites [190] and Pd NP‐functionalized Ti_3_C_2_T_x_ [233], in which ethylene perturbs Cu(I)–CNT interactions or Pd catalyzes conversion to methylenylidene species, altering charge transport. VOCs associated with ripening can also be tracked colorimetrically using pH‐sensitive dyes (see Section 2.4.1 for the underlying pH‐transduction principles) [68].

**FIGURE 6:**
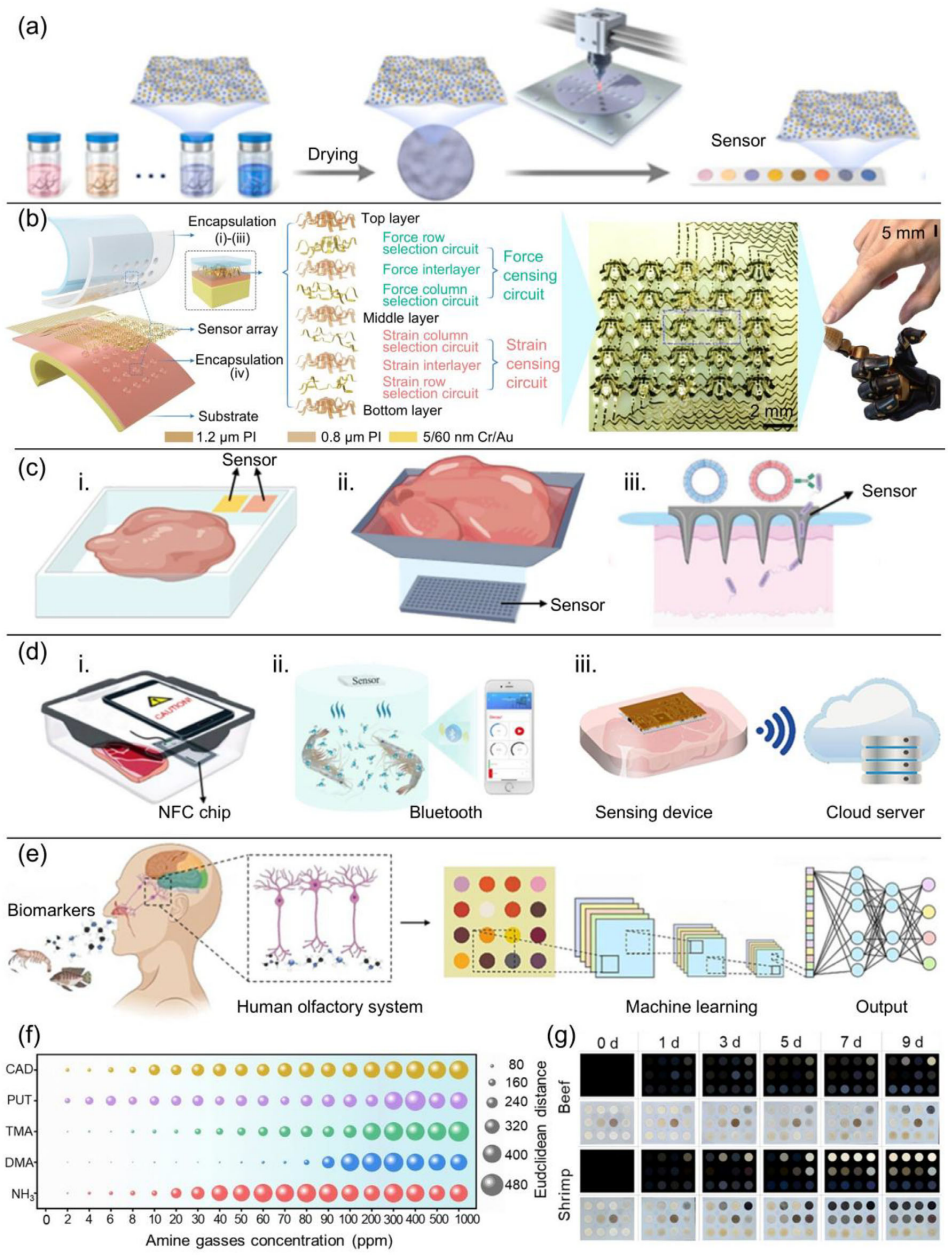
Device fabrication, sampling, and data analytics of intelligent packaging. (a) A schematic representation of the dye‐embedded array preparation procedure. Reproduced with permission [68]. Copyright 2024, American Chemical Society. (b) The schematic illustration of the multilayered construction of an 3DAE‐Skin device. Reproduced with permission [234]. Copyright 2024, The American Association for the Advancement of Science. (c) Sensor layouts and sampling modes: (i) on‐package (headspace diffusion), (ii) under‐tray (fluid localization), and (iii) microneedle backside (capillary wicking). Reproduced with permission [56]. Copyright 2023, American Chemical Society. Reproduced with permission [293]. Copyright 2024, Springer Nature. Reproduced with permission [53]. Copyright 2024, John Wiley and Sons. (d) Wireless data acquisition and connectivity: (i) NFC tagging, (ii) Bluetooth transmission, and (iii) cloud server. (Near‐field communication—NFC) Reproduced with permission [222]. Copyright 2018, American Chemical Society. Reproduced with permission [224]. Copyright 2024, Elsevier. Reproduced with permission [277]. Copyright 2024, Elsevier. (e) Human olfactory system‐inspired convolutional neural network for image‐feature extraction and learning. Reproduced with permission [282]. Copyright 2024, Elsevier. (f) Bubble chart of Euclidean distance for amine gases using a metal‐polyphenol network CSA. (g) Visual representation of meat freshness with the metal‐polyphenol network CSA. Reproduced with permission [65]. Copyright 2025, John Wiley and Sons.

Conventional firmness assessment is destructive. Inspired by human skin, a 3‐dimensionally architected electronic skin (3DAE‐Skin) that co‐integrates force and strain sensing enables rapid, non‐destructive modulus surrogates; upon contact, it analyzes spatial distribution of normal and shear forces (Figure 6b) and the resulting strain fields to discriminate ripe from unripe states in kiwi, peach, plum, and prune (Figure 5b) [234]. In a distinct, non‐destructive optical modality, a printable, passive metamaterial sticker with sub‐wavelength metallic meta‐atoms resonates at sub‐terahertz frequencies; when mounted on fruit (a multilayer reflector with exocarp and mesocarp), it excites a localized dipole plasmon confined to the exocarp and a lattice‐dependent propagating plasmon that penetrates the mesocarp. Frequency shifts of these modes report layer‐specific refractive indices—and thus ripeness—without damaging the product (Figure 5b) [235]. Beyond staging, distinguishing natural vs artificial ripening is also critical, as artificially ripened produce can pose health risks. One practical surrogate is total antioxidant content (TOC), typically lower in artificially ripened fruits and vegetables [236]. Leveraging this differential, a porous single‐atom iron nanozyme (psaFeN) with peroxidase‐like activity has been employed to establish a TOC‐responsive colorimetric assay: the nanozyme catalyzes the oxidation of 3,3′,5,5′‐tetramethylbenzidine (TMB) to its oxidized form (TMBox), yielding a measurable chromatic response, whereas endogenous antioxidants reduce TMBox back to TMB and thereby attenuate or reverse the signal. Accordingly, this assay enables non‐destructive discrimination between naturally and artificially ripened produce [237].

#### 2.4.3 Pathogen Surveillance: Biomarkers, Mechanisms, and Readouts

Pathogen detection is essential for averting foodborne outbreaks while minimizing waste and losses [7, 238]. Commercially deployed techniques are implementations of these core assay families, including phenotypic testing [239], immunodetection [240], genotypic analysis [24], metabolomics [241], and functional/activity assays [242]. For packaging‐enabled surveillance, the central goal is to translate established recognition/transduction concepts into fieldable, low‐cost formats that can operate in situ without destructive sampling or specialized infrastructure. Accordingly, pathogen sensing in packaging targets extracellular and intracellular biomarkers—including cell‐surface antigens [53, 243, 244], enzymes [54, 245], and pathogen‐emitted VOCs [23, 246, 247]—and implements them via simplified optical/electrical readouts suitable for real supply‐chain settings.

Antibody–antigen formats couple affinity recognition to colorimetric or fluorescent reporters (polydiacetylene, quantum dots, colloidal Au NPs); in a representative implementation, antibody‐labeled Au NPs agglomerate upon binding bacterial surface epitopes, shifting plasmon resonance and increasing light scattering to yield a visible red band correlating with bacterial load [53, 244, 249]. Nucleic‐acid receptors offer complementary specificity: aptamers enable constructs such as a split G‐rich DNAzyme pair (complementary to a *C. sakazakii* aptamer) that forms G‐quadruplex/hemin complexes catalyzing H_2_O_2_‐mediated ABTS oxidation to green radicals in the absence of the bacterium, whereas target binding suppresses G‐quadruplex formation and abolishes the signal [250, 251]. More generally, DNAzymes and RNAzymes furnish site‐specific cleavage of labeled probes for fluorescence recovery: a fluorophore/quencher flanking the scissile site remains quenched until target‐activated cleavage separates the pair and restores emission (Figure 5b) [245, 252, 253, 254].

Targeted, affinity‐based assays are highly sensitive but often cannot discriminate viable vs non‐viable organisms (risking false positives) and generally require a priori target selection, which constrains multiplexing given the diversity of foodborne pathogens (at least 31 known species). Routine testing is further hindered by the frequent lack of a priori knowledge of the microbial species or strains in a sample, increasing the risk of undetected contamination. To address these constraints, recent work leverages untargeted, cross‐reactive sensor arrays that read VOC metabolomes emitted by viable pathogens, providing non‐destructive, label‐free, and low‐cost detection [23, 246, 247]. For example, a paper‐based chromogenic array comprising 23 dyes/dye pairs enabled rapid, simultaneous identification and quantification of multiple viable pathogens (e.g., *E. coli* and *L. monocytogenes*) on fresh‐cut lettuce. Machine‐learning analysis further improved classification accuracy while mitigating produce‐derived VOC interference [23].

#### 2.4.4 Stability and Robustness

Colorimetric labels are attractive for in‐package monitoring because they are rapid, low‐cost, and easy to read (Figure 5c); however, deployment is often limited by stability, sensitivity, and accuracy. A major failure mode is indicator instability on paper‐like substrates: low mechanical strength and high hydrophilicity can promote structural degradation under stress or humidity cycling, leading to signal drift and dye leaching that may bias readouts and contaminate food [23, 255]. These issues motivate high‐performance bio‐based supports and protective architectures to improve mechanical integrity and moisture tolerance [256]. Multilayer laminates can reduce leaching by introducing physical barriers, but long‐term stability remains challenging [257]. Compared with physical embedding, chemical immobilization (covalent or ionic) more effectively suppresses indicator migration and improves durability under realistic conditions [213, 258, 259].

Natural pigments are appealing alternatives to synthetic dyes due to biocompatibility and end‐of‐life advantages, yet they are vulnerable to oxidative and structural degradation under light, heat, and pH excursions [201, 202, 260]. To extend functional lifetimes, encapsulation strategies—including multilayer [257] and particle‐based encapsulation (porous microspheres [261], nanoparticles [262], MOFs [218], microcapsules [263], and liposomes [264])—buffer pigments and moderate release/interaction kinetics. Beyond encapsulation, chemical and physical stabilization methods—molecular modification, co‐pigmentation, metal‐ion complexation, and pigment blending—have been explored (Figure 5f) [202]. Acylation is particularly effective: acylated anthocyanins exhibit superior color retention and enhanced thermal/pH stability, attributed to intramolecular hydrophobic interactions and *π*–*π* stacking that shield chromophores from nucleophilic attack and favor stable “sandwich‐like” conformations [265, 266, 267, 268]. Practical constraints remain, including toxic solvents, low degree of substitution, and potential hue shifts that may limit application latitude [269]. Co‐pigmentation forms noncovalent complexes with colorless co‐factors (flavonoids, organic acids, amino acids, polysaccharides, proteins), primarily driven by *π*–*π* stacking and hydrogen bonding, which stabilize the pigments and enhance color via hyperchromic effects [270]. Metal‐ion complexation (e.g., Fe^3+^/Fe^2+^/Al^3+^) with phenolic hydroxyls and related donor groups increases molecular rigidity and lowers the density of reactive sites [271]. The resulting coordination complexes organize via *π*–*π* stacking and other noncovalent interactions into supramolecular assemblies that sterically and electronically shield quinonoid chromophores from degradation [272]. Finally, pigment blending broadens chromatic response and enhances overall stability via synergistic hyperchromic effect and color modulation arising from intermolecular interactions between different pigment species [273].

Compared with colorimetry, fluorescent sensors offer higher sensitivity and better tolerance to variations in illumination, viewing angle, and background color (Figure 5d). Their solid‐state use is often limited by aggregation‐caused quenching (ACQ) of conventional luminophores [274]. Two solutions are prevalent. First, replace ACQ‐prone dyes with aggregation‐induced emission luminogens (AIEgens), which become more emissive upon aggregation and suit solid‐state platforms [216, 255, 274, 275]. For example, a pH‐responsive AIEgen, 4‐(dimethylamino)styryl)quinoxalin‐2(1H)‐one (ASQ), with a donor–acceptor architecture whose emission is modulated by protonation/deprotonation of the donor/acceptor moieties, enables ratiometric fluorescence readouts for biogenic amines and supports real‐time, non‐destructive meat/seafood monitoring when deposited on paper [274]. Second, covalent immobilization of ACQ dyes onto cellulose frameworks suppresses self‐quenching via anchoring/dilution and electrostatic repulsion, while improving processability into coatings, prints, films, and nanofibrous membranes with enhanced mechanical and environmental robustness (Figure 5g) [213, 214, 276].

For electrochemical sensors, stability is frequently constrained by power delivery. Wiring or frequent battery replacement during storage and transport undermines continuity and reliability. Integrating wireless power modules or adopting inductive sensing architectures without sustained energy input can provide steady, maintenance‐light operation across the cold chain, improving reliability, practicality, and scalability in real supply‐chain settings (Figure 5e) [63, 277].

#### 2.4.5 Rapid and Sensitive Detection

Ultrafast, high‐sensitivity responses are essential: only by detecting early biochemical changes rapidly and accurately can systems trigger timely warnings and prevent consumption of spoiled or unsafe products. Two complementary actions are required: (i) kinetics acceleration—shortening diffusion paths, expediting adsorption/desorption, and increasing interfacial reaction rates; (ii) signal amplification—increasing analyte uptake/affinity and/or transduction gain.

A broadly effective route is to introduce porosity into the sensing platform [62], which simultaneously (i) shortens mass‐transfer distances for volatile or dissolved biomarkers, (ii) increases accessible surface area and binding‐site density, and (iii) enables analyte enrichment in pores or adsorption sites—thereby accelerating kinetics and boosting signal. Consequently, filter paper and porous membranes are widely used as substrates for rapid optical labels [192, 209, 255, 257, 274]. Freeze‐drying and 3D printing can produce porous foams/films with controlled architectures for faster, more sensitive detection [194, 211, 278, 279]. In a bio‐based PVA/polyvinylpyrrolidone/microcrystalline cellulose (MCC) foam prepared by freeze‐drying and functionalized with anthocyanins, the incorporation of MCC increased porosity to ≈79.6% and reduced pore size, thereby accelerating response kinetics 6‐fold and yielding stable, visible color changes within 10 s under acidic or basic vapors [211]. As a complementary approach, loading dyes/fluorophores on porous carriers (porous microspheres [261] or MOFs [68, 219]) places indicators at open surfaces for immediate contact while pore networks promote rapid diffusion. Beyond high surface area and porosity, porous frameworks such as MOFs [64, 218], COFs [56], and HOFs [67] can be chemically programmed to present binding motifs/chemical environments that selectively recognize target biomarkers, further accelerating response and increasing sensitivity (Figure 5h). Illustratively, a Cu‐MOF enriched with unsaturated Cu sites was employed for NH_3_ coordination. Embedding this MOF in a starch/PVA matrix yielded composite films with high specific surface area (546.11 m^2^ g^−^^1^) and hierarchical porosity, enabling response times < 1 min and an LOD of 0.8 mM for NH_3_ [218]. Likewise, in situ growth of pH‐dependent imine COFs on silk fibroin films enabled visual detection of putrescine within 0.4 s, orders of magnitude faster than conventional polymer‐embedded colorants [56].

While dry‐state platforms are common, embedding indicators in hydrogel matrices provides complementary means to accelerate response and enhance sensitivity [55, 67, 215, 237, 248, 280, 281]. High water content and open network promote rapid diffusion/partitioning to active sites, stabilize dyes (reducing aggregation), and maintain optical contrast—shortening transport/reaction time constants and improving signal‐to‐noise. For instance, a bilayer hydrogel for H_2_S colorimetric detection combined a sensing layer (PVA/boric acid/Pb^2+^) with an adsorption/regeneration layer (polyacrylamide/sodium alginate) [55]. In the hydrated, porous sensing layer, H_2_S dissociates to S^2−^, diffuses to Pb^2+^, and forms PbS, yielding robust color change with broad range (0.2–100 ppm), 10 s response, and LOD = 0.026 ppm. The alginate‐containing layer (less porous, chelating‐site rich) competitively complexes Pb^2+^ (“egg‐box” coordination), enabling 32 s recovery and reusability—an inexpensive, environmentally friendly strategy for egg freshness monitoring [55]. Similarly, embedding dual‐emissive, ratiometric HOFs (Eu@HOF‐12) in agarose hydrogel enabled BA detection with < 6 s response and LODs of 1.8–5.3 µM across different BAs [67]. The soft, conformable nature of hydrogels also improves contact with irregular foods, thinning the stagnant boundary layer and improving analyte flux/capture efficiency—further underpinning faster kinetics and stronger signals in real packaging scenarios [281].

Apart from matrix engineering, tailoring the sensing center directly boosts speed and sensitivity. For natural pigments, metal‐ion complexation and rational pigment blending amplify intensity (hyperchromic/hypochromic effects) [202]. More fundamentally, decorating sensing centers with metal ions accelerates recognition by coordinating amines (e.g., NH_3_) to the metal center, improving both kinetics and sensitivity [65, 224]. In a metal–polyphenol competitive coordination platform (Fe^2+^‐TA), amines bind preferentially to the Fe center. Ultrafast excited‐state dynamics—relaxation of Fe^2+^‐TA‐NH_3_ at ∼10 ps compared with ∼60 ps for Fe^2+^‐TA‐NaOH—indicate that amine coordination triggers an earlier ligand‐to‐metal charge‐transfer event, yielding an LOD of ≈300 ppb and high amine sensitivity [65]. In conductive polymers, FeCl_3_‐doped poly(3,4‐ethylenedioxythiophene):poly(styrenesulfonate) (PEDOT:PSS) illustrates how intercalation/catalysis co‐amplify signals: Fe^3+^/H^+^ intercalation coordinates with PSS‐SO_3_^−^, expands PEDOT‐rich domain spacing, and adds ionic carriers/percolation pathways; upon NH_3_ exposure, FeCl_3_ catalysis/spillover enriches NH_3_ at the interface, depleting holes and raising resistance, while Lewis acid–base pairing (NH_3_ + H^+^ → NH_4_^+^) releases electrons that recombine with holes in the p‐type film—together producing ∼25% response at 25°C to 1 ppm NH_3_ with LOD = 0.23 ppm [224]. The same enrichment‐plus‐detection logic advances fluorescent probes: a functional ionic liquid, 7‐hydroxycoumarin quaternary phosphonium (7‐HDCP), uses a long‐chain phosphonium cation to solvate/preconcentrate NH_3_ via electrostatics and weak hydrogen bonding, delivering < 11 s response and LOD = 0.12 ppm [220]. Probes relying on labile noncovalent recognition (e.g., hydrogen bonding) offer ultrafast, reversible kinetics without bond scission/formation. Two diphenyl acridine‐based fluorophores form a hydrogen bond between the 10‐position H (H_α_) and BAs, reducing charge at H_α_ and increasing it on the acridine N, thereby enhancing ICT, red‐shifting emission, and lengthening lifetimes; the resulting ratiometric films show LOD = 1.3/2.6 ppm and 15/25 s response [206].

#### 2.4.6 Accuracy: Device Fabrication, Sampling, Readout, and Data Analytics

Accuracy is the gating criterion for commercial deployment: any misclassification can trigger premature waste or overlook genuine safety risks. Achieving high accuracy is challenging because uncertainty arises from sensor drift, environmental variability (illumination, temperature, humidity), sensor–reader coupling, and food matrix heterogeneity. Robust performance therefore requires co‐design of hardware, sampling protocols, and data analytics. Three elements are essential: (i) sensor platforms that selectively resolve multiple indicators with minimal interference; (ii) sampling architectures that acquire representative biomarkers reliably; and (iii) readout and analytical pipelines that process complex, multivariate signals with resilience to noise.

Physical dip‐/drop‐coating of dyes often yields spatially non‐uniform films, causing misreads and poor reproducibility [23, 209, 255, 274]. Embedding dyes in‐matrix during fabrication improves homogeneity, mitigates local aggregation, and stabilizes signal output (Figure 6a) [282]. Fluorescent routes decouple readouts from background color and ambient illumination, and enable more quantitative monitoring by combining intensity and wavelength channels [209, 220, 281]. Single‐channel on/off schemes, however, remain vulnerable to photobleaching, scattering, and excitation fluctuations [64, 213]. Ratiometric fluorescence addresses these issues by pairing an analyte‐responsive luminophore with an internal reference, enabling self‐calibration and stronger anti‐interference [206, 213, 214, 283]. For example, covalently immobilizing fluorescein isothiocyanate (FITC, responsive) and protoporphyrin IX (PpIX, reference) on CA produced a dual‐emission tag that shifted from red to orange/yellow/green with increasing ammonia. The ratiometric response exhibited linearity with respect to log_10_[NH_3_] from 5.0 ppm to 2.5 × 10^4^ ppm and supported quantitative shrimp/crab freshness assessment, consistent with TVB‐N and microbiology [213]. Nonetheless, UV‐excited systems are susceptible to intrinsic food autofluorescence and may induce photodamage [284, 285, 286]. Upconversion nanoparticles (UCNPs) excited in the near‐infrared (NIR) suppress autofluorescence and mitigate photodamage. As a representative implementation, an amino‐functionalized UCNPs/curcumin single‐particle FRET probe uses UCNPs as energy donors and curcumin as the acceptor for freshness monitoring. In the presence of BAs, the diketone groups of curcumin are converted to enolate ions, increasing spectral overlap and triggering FRET: non‐radiative absorption near 540 nm quenches the UCNP green emission and produces a visible green‐to‐red shift. The probe operates with near‐zero background, shows negligible interference from common amino acids, and detects BAs with an LOD of 2.73 µM [281].

Sampling strategy critically impacts accuracy, especially for pathogens, where both localization and accessibility govern detection accuracy. Freshness biomarkers typically partition into the headspace, so surface‐mounted labels suffice (Figure 6c). Pathogens are more elusive: contamination can localize on surfaces, within microcavities, or inside tissues, and exudates are spatially heterogeneous. To overcome undersampling, structure‐informed packaging integrates sampling, concentration, and sensing in closed systems. Inclined packaging trays (≈45°) with a buffer‐infused membrane localize fluids at a reagent (such as nucleic acid probe)‐immobilized, antifouling interface and enable in‐package, hands‐free detection of *Salmonella* down to 10^3^ CFU g^−1^ in whole chicken, even when contamination originates from gloves, knives, or countertops (Figure 6c) [54]. Beyond surface contamination, internal pathogens pose an equally critical threat—especially in meat, poultry, fish, and leafy greens—where microorganisms embedded within tissue resist conventional washing. Porous microneedle arrays have therefore been engineered to physically penetrate food tissues (and, when required, the packaging), wicking interior fluids toward the sensor via capillary action (Figure 6c) [53, 248]. Locating sensing elements on the backside of the microneedle patch enables detection without direct food contact or package opening, thereby preventing secondary contamination and facilitating integration into downstream supply chains, including retail and household settings [53]. These approaches shift from passive, surface‐only detection to active, geometry‐assisted sampling, improving capture efficiency and reliability in realistic supply chains.

For electrochemical labels, accuracy is often constrained by benchtop instrumentation, wired readouts, variable reader–sensor spacing, motion artifacts, and power management during storage and transport [63, 287]. Battery dependence can interrupt continuous monitoring, while cable‐based measurements introduce latency and potential data loss. Embedding low‐cost wireless circuits directly on the label improves robustness and portability. Near‐field communication (NFC) provides passive, battery‐free operation through inductive coupling with smartphones; the same field powers the sensor front end and enables bidirectional data exchange, allowing potentiometric/impedimetric freshness signals to be digitized on‐tag and read in real time via an app (Figure 6d) [63, 222]. Bluetooth modules support continuous streaming over intermediate distances with modest power budgets, enabling continuous transmission of electrochemical freshness signals to paired smart devices during storage and transport (Figure 6d) [224, 288]. Wi‐Fi extends range and throughput for high‐density deployments and direct cloud uplink, supporting fleet‐level dashboards, predictive analytics, and early‐warning alerts (Figure 6d) [224]. The convergence of wireless electrochemistry with Internet of Things (IoT) backends thus delivers end‐to‐end visibility across the cold chain by streaming real‐time freshness data among production, storage, transportation, and retail nodes. This IoT‐enabled monitoring moves intelligent packaging from laboratory prototypes toward scalable industrial deployment, improving consumer safety, reducing food waste, and strengthening supply‐chain resilience [277].

Optical readouts face accuracy limits when interpreted by eye: single‐hue changes are subjective, lighting‐dependent, and subtle at low analyte levels. Digitizing signals via cameras, colorimeters, or smartphones (RGB/Lab, ΔE) improves precision and usability but remains device‐dependent without spectral calibration, requiring white standards, controlled white balance, and multispectral illumination [206, 217, 289, 290, 291]. Single‐analyte “lock‐and‐key” labels also struggle to deconvolve mixtures of biomarkers that jointly define quality, showing limited diagnostic accuracy [66, 237]. CSAs address this by producing multidimensional, cross‐reactive patterns that mimic biological olfactory fingerprints and enable sensitive, multi‐analyte detection at trace levels [282, 292]. Yet the high dimensionality of CSA outputs precludes direct visual interpretation, necessitating computational pattern‐recognition methods for decoding [62]. Classical chemometrics, namely principal component analysis (PCA), linear discriminant analysis (LDA), hierarchical clustering analysis (HCA), and linear regression, can classify and quantify targets—for instance, a psaFeN three‐channel platform distinguished five antioxidants (LOD 310 nmol L^−1^) and separated artificially from naturally ripened fruits via LDA [237]. Yet feature‐engineering approaches hinge on manually specified correlations (e.g., mapping a particular dye response to microbial contamination or spoilage), which constrains their ability to capture the multifactorial, nonlinear signatures of food deterioration and safety risks [23]. Machine learning helps overcome this bottleneck by learning directly from raw, digitized CSA outputs without manual feature selection. For example, RGB data from a 23‐dye CSAs were used to train a multilayer neural network (NN), yielding 91%–95% accuracy for strain‐specific pathogen identification and quantification [23]. Nonetheless, conventional NNs can struggle with large‐scale, high‐dimensional, nonlinear datasets. In contrast, convolutional neural networks (CNNs) extract hierarchical spatial features from image‐like CSA data, improving recognition accuracy and robustness while reducing human bias in feature extraction (Figure 6e) [65, 66, 230, 282]. As a representative case, a CNN trained on ∼5000 samples from a metal–polyphenol network CSA achieved 99.83% accuracy for meat freshness classification (Figure 6f,g) [65]. Coupled with smartphone apps, Bluetooth connectivity, and cloud platforms, CNN‐enabled CSAs support real‐time, remote monitoring across the food supply chain—from processing to retail and consumer endpoints—advancing intelligent, scalable, and user‐accessible food‐quality assurance [55].

In summary, intelligent packaging safeguards food quality and safety by transducing biomarker dynamics into optical or electrochemical signals with sufficient stability, sensitivity, and accuracy for decision‐making. For perishables, indirect (pH‐coupled) and direct interactions with TVB‐N, biogenic amines, VOCs, and H_2_S underpin colorimetric, fluorescent, and electrochemical labels that enable non‐destructive, rapid, in situ monitoring. Performance has been advanced through matrix engineering (e.g., porous/hydrogel scaffolds for accelerated mass transfer), chromogenic/fluorogenic design (e.g., ratiometric luminophores, AIEgens, metal–polyphenol coordination), and device architectures (e.g., on‐tag immobilization, wireless NFC/Bluetooth readouts), collectively improving limit of detection, response time, drift resistance, and operational robustness. Integration with smartphones and machine‐learning analytics further boosts quantitative accuracy and mitigates operator subjectivity.

### 2.5 Superhydrophobic Packaging

Bio‐based packaging has advanced markedly; however, deployment for high‐moisture foods (e.g., meat, milk, honey) and in humid environments remains constrained by intrinsic hydrophilicity of biopolymers and their inadequate water resistance [60]. Excess water uptake leads to swelling, structural collapse, and even rupture, degrading mechanical integrity, barrier performance, and service life [296, 297, 298, 299]. Enhancing interfacial water repellency is therefore a first‐order design objective. Although fluorinated chemistries provide ultralow surface energy and durable repellency [300, 301], concerns over persistence, limited biodegradability, and potential toxicity motivate safer, sustainable alternatives [58, 302]. Incorporation of naturally derived lipids, oils, and waxes—via bulk blending or Pickering emulsions—can raise hydrophobicity and lower water vapor transmission [19, 303, 304], and applying wax‐rich outer layers onto hydrophilic matrices—by extrusion blowing, spray‐coating, or dip‐coating—further improves moisture resistance, expanding applicability in high‐humidity or wet‐contact scenarios [305, 306]. Nonetheless, these gains are usually modest: water contact angles commonly remain ≤ 110°, which is insufficient for stringent, real‐world packaging demands, especially during prolonged cold‐chain storage. The following sections therefore outline design principles and scalable fabrication routes for bio‐based superhydrophobic packaging that deliver substantially improved water resistance and durability (Table 5).

**TABLE 5:** Superhydrophobic packaging for preserving perishable foods.

Biomimetic template	Structure	Hydrophobic‐side fabrication	Packaging system	Food	Functions	θ (°)	Refs.
Lotus leaf	Janus	Biomimetic templating	SPS/Ag NPs/gelatin	Chicken; pork; grape	Superhydrophobicity	156.6	[311]
Lotus leaf	Bilayer	Biomimetic templating	Chitosan/PDMS/sodium alginate/zein NPs	Fresh‐cut apple; lotus root	Hydrophobicity	>130	[57]
Pomegranate pulp	**/**	Spin coating	Anthocyanin/starch NPs/stearic acid	Shrimp	Superhydrophobicity; self‐cleaning	>154	[315]
Lotus leaf	/	Solution immersion	Starch nanofibers/stearic acid	/	Hydrophobicity; self‐cleaning	134.7	[297]
Rose petal	**/**	Electrospinning; electrospraying	Starch nanofibers/acylated TA	Cherry tomato; cherry; blueberry	Hydrophobicity	134.1	[310]
Taro leaf	Three‐tier hierarchy	Heating–cooling recrystallization	CNF/alkyl ketene dimer/cellulose microparticles/TiO_2_ NPs	Tomato	Superhydrophobicity; self‐cleaning; mildew‐proofing; anti‐rotting	166.7	[61]
/	/	Spraying	Arnebia euchroma/beeswax‐SiO_2_/filter paper	Shrimp	Superhydrophobicity; icephobicity; anti‐counterfeiting	156.2	[192]
/	**/**	Spin/spray/dip coating	Phase‐transited lysozyme/carnauba wax	Milk; yogurt; honey; beverages	Superhydrophobicity; antifouling	>150	[309]
/	Janus	Electrospinning	PCL/chitosan/PEO/thymol	Pork	Directional liquid transport	130.8	[60]
Lotus leaf	Janus	Electrospinning	Stearic acid/anthocyanin/curcumin/TA NPs/konjac glucomannan/tea polyphenols	Pakchoi; fresh‐cut potato	Superhydrophobcity; antifouling	154.5	[317]
*Canna* leaf	Sandwich‐structured	Roll‐to‐roll imprinting and evaporation‐induced self‐assembly	Cellulose@ZnO/Arnebia euchroma/stearic acid	Shrimp; pork	Superhydrophobicity; icephobicity; self‐cleaning; antifouling	155.1	[296]
Lotus leaf; red blood cell; mussel‐adhesion proteins	Janus	Electrospinning	Modified CNCs/ethylcellulose/konjac glucomannan	Chicken; pork; grape	Superhydrophobicity; antifouling	150.5	[58]
Swan feather	/	Biomimetic templating	CMC/PVA/quercetin	Shrimp; pork	Hydrophobicity; anti‐counterfeiting	138	[51]
/	/	Extrusion blowing	Starch/gelatin/beeswax	Dumplings; milk powder; chili oil	Hydrophobicity; self‐cleaning	106	[305]
/	Janus	Chemical vapor deposition	Cellulose/curcumin/methyltrichlorosilane	Fish fillet	Hydrophobicity	110.5	[59]

#### 2.5.1 Nature‐Inspired Superhydrophobic Surfaces

**FIGURE 7:**
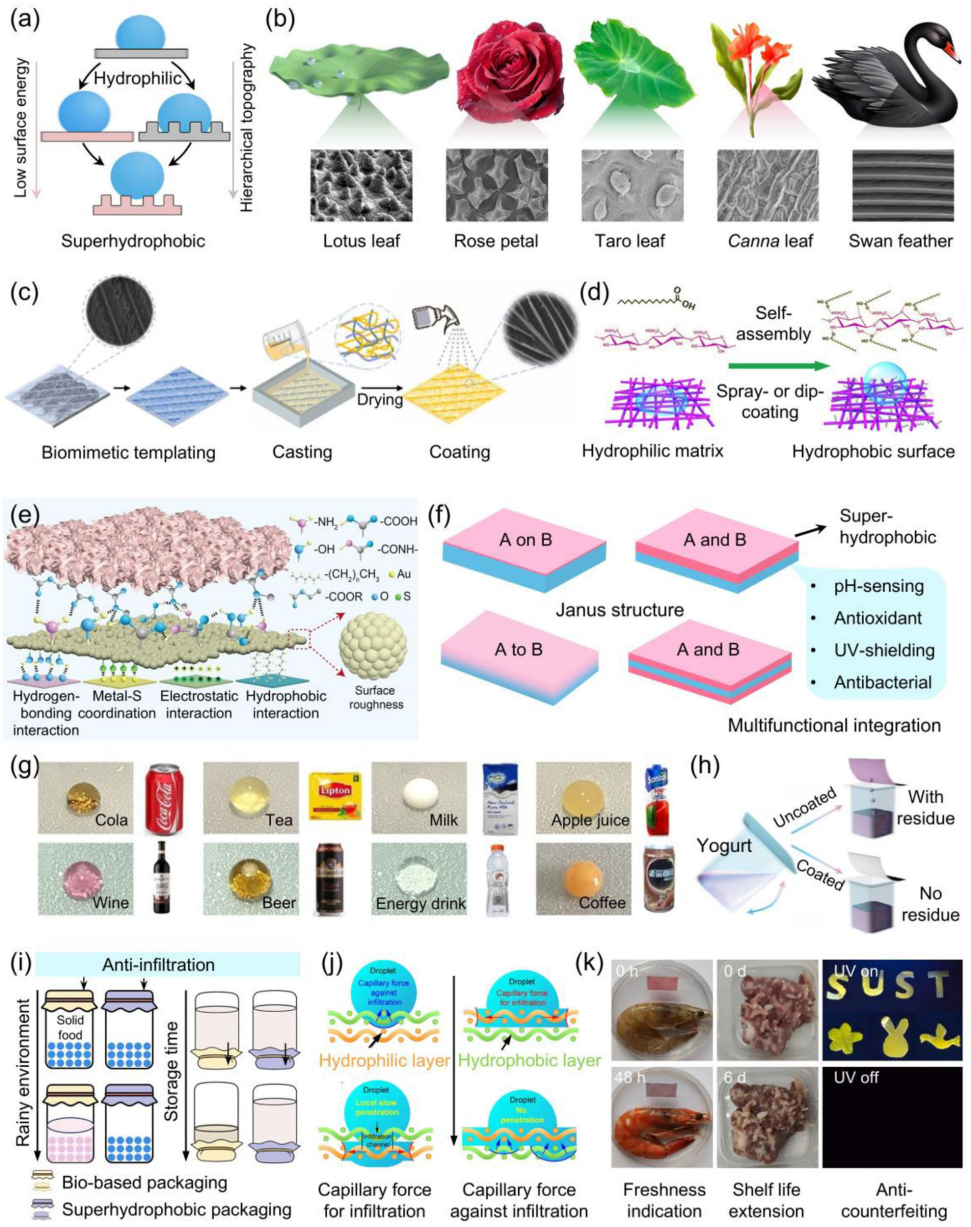
Design, fabrication, and applications of superhydrophobic packaging. (a) Design principles of superhydrophobic surfaces. (b) Various natural templates for superhydrophobic interfaces. Reproduced with permission [311]. Copyright 2023, Elsevier. Reproduced with permission [307]. Copyright 2015, Elsevier. Reproduced with permission [310]. Copyright 2024, Elsevier. Reproduced with permission [61]. Copyright 2025, Elsevier. Reproduced with permission [312]. Copyright 2024, Elsevier. (c) Biomimetic templating (swan feather) to fabricate superhydrophobic films. (d) Self‐assembly of hydrophobes on bio‐based matrices via spray‐ or dip‐coating to prepare superhydrophobic coatings. Reproduced with permission [297]. Copyright 2021, American Chemical Society. (e) Molecular scale adhesion strategies. (f) Common Janus configurations for multifunctional integration. (g) Water contact angles of an edible superhydrophobic interface with commercial liquid foods. Reproduced with permission [313]. Copyright 2018, American Chemical Society. (h) Yogurt residues on lids before/after superhydrophobic coating after shaking. Reproduced with permission [309]. Copyright 2021, Elsevier. (i) Anti‐infiltration concept for superhydrophobic packaging. (j) Mechanism of Janus electrospun fibers to prevent liquid penetration: droplets infiltrate the hydrophobic/hydrophilic (top/bottom) layers and cause penetration and hydrophilic/hydrophobic (top/bottom) layers prevent penetration. Reproduced with permission [58]. Copyright 2024, Elsevier. (k) Multifunctional superhydrophobic packaging for preservation and quality monitoring. Reproduced with permission [296]. Copyright 2023, Elsevier. Reproduced with permission [51]. Copyright 2024, Elsevier.

Superhydrophobicity is typically defined by an apparent water contact angle θ ≥ 150° and, in practical contexts, a low sliding angle [307]. As rationalized by the Wenzel and Cassie–Baxter models, such extreme water repellency arises from the synergy between low‐surface‐energy chemistry and well‐organized multiscale roughness (hierarchical micro/nanostructures) that stabilizes an air cushion at the solid–liquid interface (Figure 7a) [307, 308]. These principles are ubiquitous in nature and have inspired biomimetic packaging materials modeled after lotus leaves [309], rose petals [310], taro leaves [61], *canna* leaves [296], and swan feathers (Figure 7b) [51]. Among these archetypes, lotus leaves are most studied: micron‐scale papillae decorated by self‐assembled wax nanostructures jointly produce large θ and small sliding angles, enabling self‐cleaning.

Building on these biological blueprints, bio‐based superhydrophobic films/coatings have been fabricated using natural hydrophobic components and structure‐directing routes. One direct strategy is biomimetic templating of natural template‐like topographies (Figure 7c) [57, 311]. Typically, a PDMS negative is cast from a fresh lotus leaf/swan feather, then a natural‐polymer solution is molded against the PDMS to replicate the micro/nano relief. For example, soybean‐polysaccharide solutions cast on a lotus‐leaf PDMS template yielded microdot features (≈6.2–16.1 µm); subsequent spin/spray deposition of carnauba wax formed lamellar nanostructures, producing θ = 157.2° [311]. Beyond templating, lotus‐like roughness can also arise from the self‐assembly of hydrophobic components on bio‐based matrices via spray‐ or dip‐coating [296, 297, 313, 314, 315]. Wax emulsions (e.g., beeswax, candelilla, rice‐bran wax) sprayed onto diverse substrates create botryoidal microtextures with embedded nanofolds or hierarchical flower‐like hierarchies, imparting excellent water repellency without complex processing [313, 314]. As another illustration, immersing electrospun starch nanofiber mats in stearic‐acid/ethanol drives hydrogen bonding, hydrophobic association, and van der Waals‐mediated assembly of flower‐like micro/nanostructures (Figure 7d). Modulating stearic acid concentration tunes the hierarchical surface architecture and increases the water contact angle from approximately 0° to 134.7° [297].

Nature also provides high‐adhesion superhydrophobic states. Rose petals present conical micropapillae overlaid with cuticular nanofolds, which are larger than those on lotus leaves. Droplets penetrate between papillae but not into the nanofolds, yielding an intermediate wetting regime between Wenzel and Cassie–Baxter—combining high contact angle with strong pinning [307, 316]. A related biomimetic surface was obtained by electrospraying acylated TA onto electrospun starch nanofiber films. Deposited droplets self‐assembled into convex microstructures with irregular nanofolds resembling petal surfaces [310]. The acylated TA provided hydrophobicity with a contact angle of about 134.1° while retaining antioxidant and antibacterial functions. Acylation also strengthened bonding to the catechol‐rich starch matrix and improved coating stability. The films maintained local humidity, suppressed microbial growth, and extended cherry tomato shelf life to roughly 15 days. Taro leaves exhibit a more complex three‐tier hierarchy —primary micro‐bumps (10–20 µm), secondary epicuticular wax platelets (<1 µm), and tertiary wax NPs embedded within the platelets [61]. A Pickering emulsion strategy reproduced this architecture using cellulose microparticles to represent micro‐bumps, recrystallized alkyl ketene dimer to form platelets, and TiO_2_ NPs as embedded features. Controlled cooling generated multiscale roughness and achieved a water contact angle of approximately 167°, comparable to natural taro leaves. When used as packaging paper, the coating preserved tomato freshness for up to 14 days [61].

#### 2.5.2 Adhesion Engineering for Interfacial Durability

Durable superhydrophobic packaging relies on robust adhesion between the hydrophobic overlayer and its supporting substrate; interfacial failure rapidly degrades repellency and barrier performance. Three complementary routes are effective to enhance adhesion: (i) enlarging the real contact area, (ii) introducing specific interfacial chemistries (e.g., hydrogen bonding, covalent bonding, and coordination/electrostatic interactions) (Figure 7e), and (iii) inserting compliant or cross‐linking interlayers. Increasing contact area with a hot‐wax treatment promotes formation of a continuous interface and reinforces noncovalent adhesion (van der Waals forces) at the coating–substrate boundary [314]. Chemical coupling affords higher durability: grafting cinnamic and myristic acids onto CuO introduces carboxyl and hydroxyl groups that react at the interface to form covalent linkages, thereby improving stability under use conditions [302]. Cross‐linker‐mediated strategies further enhance anchorage. PDMS can tether beeswax–SiO_2_ composite particles to paper through hydrogen bonding, reducing peel‐off during handling, while an elastic arabic‐gum/gelatin interlayer serves as a tough adhesive matrix that bonds beeswax to the substrate via hydrogen bonding and van der Waals forces and accommodates mechanical deformation [192, 313]. Bio‐derived primers add additional versatility: cysteine‐triggered thiol‐disulfide exchange yields phase‐transitioned lysozyme nanofilms rich in –OH, –NH_2_, and –COOH groups that adhere strongly to metals, inorganics, and polymers through hydrogen bonding, metal–sulfur coordination, electrostatic attraction, and hydrophobic interactions (Figure 7e). Subsequent carnauba wax deposition yields water contact angles above 150°, maintaining repellency under abrasion, bending, and dynamic water‐jet exposure [309]. Collectively, these adhesion‐engineering approaches improve mechanical robustness, preserve Cassie–Baxter wetting, and reduce liquid‐food residue, addressing persistent limitations in real‐world packaging (Figure 7g–j).

#### 2.5.3 Structure‐Tailored Multifunctional Integration

Integrating sensing, antimicrobial, or UV‐blocking into superhydrophobic packaging remains challenging because the additives and surface chemistries that enable activity are often hydrophilic and therefore reduce water repellency [51, 296, 315, 317, 318], while highly water‐repellent surfaces can in turn hinder analyte transport and slow response [296]. A widely effective solution is to use Janus or multilayer architectures with asymmetric wettability, which spatially decouple these conflicting requirements—placing active, hydrophilic domains where mass transfer is needed and retaining an outer superhydrophobic barrier for moisture protection (Figure 7f) [319]. Such laminates are compatible with casting [57], coating [311], layer‐by‐layer assembly [58, 317], and spinning, and are often inspired by natural wetting hierarchies.

A representative Janus film combining soybean polysaccharide (SPS)‐stabilized silver nanoparticles within an SPS/gelatin/beeswax hydrophilic layer, overlaid with a carnauba‐wax superhydrophobic skin (θ = 156.6°) by spin/spray coating, achieves robust water repellency alongside UV‐blocking, antibacterial, and antioxidant functions [311]. This design reduces weight loss, limits browning, and suppresses microbial growth in perishable products. A feather‐inspired bilayer extends this concept by templating a quercetin‐loaded CMC/PVA matrix into barbed micro/nanostructures, followed by carnauba‐wax deposition to mimic the lipidized exterior of swan feathers. The resulting film combines pH sensing, fluorescence, antioxidant activity, and antibacterial protection in its inner layer with a superhydrophobic outer barrier, enabling real‐time freshness monitoring and extending pork shelf life by approximately three days (Figure 7k) [51]. Moving beyond bilayers, a *canna* leaf‐inspired sandwich architecture (upper epidermis, middle leaf pulp, and lower epidermis) integrates superhydrophobic, icephobic, and colorimetric functions via roll‐to‐roll imprinting and solvent‐evaporation‐induced self‐assembly [296]. Here, the top layer, comprising hemispherical microflowers (50–60 µm) with stearic acid/naphthoquinone nanoflakes (∼496 nm), imparts superhydrophobic behavior, ice resistance, and freshness‐indicating color change; a hydrophilic cellulose/ZnO middle layer readily imbibes moisture and accelerates TVB‐N uptake; and a stearic acid‐nanoflake bottom layer acts in concert with the top layer to suppress local over‐swelling of the middle layer. This laminate exhibits a water contact angle of 155.1°, delays ice formation from 45 to 265 s, and reduces ice adhesion from 599.72 to 45.53 kPa—values lower than those of typical de‐icing surfaces [320]—while enabling accurate, ice‐tolerant freshness monitoring of shrimp and pork.

Despite these advances, scaling Janus‑structured superhydrophobic packaging is often limited by the trade‐off between anisotropic precision and manufacturing scalability. Common fabrication methods include self‐assembly, phase separation, microfluidic synthesis, masking modification, Pickering‐emulsion–enabled approaches, spin/spray coating with lamination, electrospinning/electrospray, one‐step in situ routes, and surface‐selective chemical modification [57, 58, 317, 321, 322]. Self‐assembly and phase separation offer scalability but suffer from sensitivity to processing conditions, leading to variability in Janus contrast and domain size [309, 321]. Microfluidic synthesis provides exceptional morphological control but suffers from intrinsically low throughput [322]. Masking and Pickering‐emulsion strategies can define anisotropy more explicitly, yet they are frequently batch‐type and require tight control of templating/emulsion stability [321]. Spin/spray coating with lamination is highly compatible with existing converting lines but demands robust interfacial adhesion to avoid delamination under humidity cycling and handling. Electrospinning/electrospray enables porous Janus fibrous layers with fast transport, yet industrial adoption hinges on higher throughput, uniformity, and mechanical reinforcement while maintaining anisotropy after winding and sealing [58, 323]. One‐step in situ routes reduce assembly steps but can be formulation‐ and environment‐sensitive, complicating reproducibility, whereas surface‐selective chemical modification provides precise wettability tuning but often involves multi‐step chemistry, solvent use, and food‐contact compliance constraints [322]. Overall, industrial feasibility must be assessed method‐by‐method, prioritizing routes compatible with continuous converting and evaluating throughput‐relevant metrics such as processable window, handling robustness, and batch reproducibility, rather than laboratory‐level Janus precision alone.

The remarkable water repellency and functional robustness of superhydrophobic packaging arise from the coordinated action of surface chemistry and hierarchical topography. This coupling is especially critical for bio‐based matrices, which are inherently hydrophilic and mechanically less robust than many synthetic counterparts. Guided by structure–function principles, biomimetic multiscale designs combine microscale features (papillae, bumps, dots) that entrap air and reduce solid–liquid contact with nanoscale elements (nanoflakes, nanofolds, platelets) that raise capillary resistance and stabilize Cassie–Baxter wetting. In parallel, incorporation of low‐surface‐energy constituents—long‐chain fatty acids (e.g., stearic acid), plant waxes (e.g., carnauba, beeswax), and hydrophobes (e.g., alkyl ketene dimers)—further depresses surface free energy, enabling high contact angles with tunable droplet adhesion, as inspired by lotus leaves, rose petals, and taro leaves. Building on these wetting architectures, structure‐tailored integration—particularly Janus and multilayer constructs—co‐deploys sensing, antimicrobial, icephobic, and UV‐shielding functions while preserving outer‐surface repellency and controlling mass‐transfer. Equally important, durable interfacial adhesion—achieved by enlarging real contact, introducing specific interfacial chemistries, or inserting compliant/cross‐linking interlayers—maintains coating retention and superhydrophobic performance under bending, folding, abrasion, and repeated handling, and therefore is a co‐equal design priority. Looking ahead, successful translation will depend on scalable, environmentally benign, and cost‐efficient manufacturing routes that deliver these hierarchical chemistries and structures at industrial throughput while ensuring food‐contact safety and long‐term reliability.

### 2.6 Design Paradigms and Standardized Benchmarking

Multifunctional bio‐based packaging is increasingly designed through the superposition, coupling, and/or synergy of functions. Superposition denotes the stacking of largely independent modules, whereas coupling implies mechanistic linkage such that activating one function directly regulates another. Synergy represents a quantitative claim: the combined outcome exceeds that expected from individual contributions under an explicit additivity model, and therefore requires component‐resolved controls for validation. For example, a Fe‐MoO_x_ platform combined photothermal heating with thermally triggered curcumin release, and its synergistic antibacterial action was rigorously quantified using a combination‐index framework [155]. Similarly, ratio‐dependent interaction mapping in multi‐antioxidant mixtures revealed synergistic regimes that ultimately translated into ∼2–4‐fold shelf life extension in refrigerated meat systems [324]. In practice, however, “synergy” is often invoked based on qualitative performance gains without quantitative validation; the observed gains frequently reflect simple superposition or mechanistic coupling rather than model‐defined synergy. Accordingly, throughout this and the following sections, we adopt the umbrella term multifunctional integration to describe the co‐location and coordinated operation of multiple functionalities—whether superposed, coupled, or potentially synergistic—within a single packaging platform.

#### 2.6.1 Design Paradigms for Multifunctional Integration

The most commonly explored routes toward multifunctional integration in bio‐based packaging begin with “single‐function upgrades,” with antibacterial and antioxidant functions most frequently targeted. This prevalence stems from the intrinsic redox activity of many bio‐derived antimicrobials, which renders antioxidant activity a common co‐benefit. The most straightforward approach is compositional co‐integration of distinct active classes—plant‐derived compounds (e.g., chitosan, essential oils, polyphenols) combined with metals/metal oxides, carbon dots, or nanozymes—such that membrane disruption, metal ion stress, and ROS‐mediated pathways coexist within a single antibacterial (or antioxidant) objective [57, 325, 326]. Beyond composition, coupling chemical functions to structural design has proven powerful. For instance, TA/gallic acid coatings integrated with nanoimprinted nanopatterns on polylactic acid (PLA) enable mechanical interference and chemical interactions to jointly enhance disinfection beyond either strategy alone [327]. As discussed in Section 2.3.2, chemo–optical coupling can further intensify antimicrobial performance via heat/ROS‐enabled amplification and gated transport.

As multifunctional integration progresses beyond single‐function upgrading, a natural next step is the superposition of antibacterial and antioxidant functions. Because many actives inherently deliver both, or can be paired by simply combining complementary components, this dual‐functionality is readily achievable. A modest increase in complexity involves integrating this bioactive core with barrier regulation (and, in some cases, respiration suppression)—coupling active protection with passive control of moisture/oxygen transport and the downstream quality loss they accelerate [110]. Further complexity arises when active packaging is integrated with intelligent functions [56], where response sensitivity and reliability become tightly coupled to mass transport (diffusion, partitioning, and release). Such systems demand deliberate transport‐aware design rather than additive formulation. An even higher integration level is reached when active modules are combined with radiative cooling [88, 90, 92], modified atmosphere control [111, 112, 113, 114, 115], or superhydrophobicity [51, 310, 317], where multifunctional performance is governed by structural coupling—as highlighted in Section 2.5.3 on structure‐tailored multifunctional integration (e.g., Janus architectures). In such systems, the color/optical properties, wettability, migration, and release of active components can reshape hierarchical structures and, in turn, alter reflectance/emissivity, pore‐enabled gas transport, and surface hydration states. Consequently, the integration of cooling, atmosphere regulation, and superhydrophobicity typically demands much finer multiscale structural design and control, and remains in its early stages.

#### 2.6.2 Standardized Benchmarking for Multifunctional Packaging

To enable meaningful performance comparison across diverse systems and testing protocols, we adopt the shelf life multiplier as a primary benchmark [48], defined as:

$$\mathrm{shelf}\;\mathrm{life}\;\mathrm{multiplier}=S_{\mathrm{pack}}\;/\;S_{\mathrm{bare}}$$

where *S*_pack_ is the shelf life of the food under the tested packaging (or coating) condition and *S*_bare_ is the shelf life of the corresponding uncoated/unpackaged control, both determined based on the last day when the food remains unspoiled.

Table 6 compiles representative bio‐based systems and maps their integrated functions using a unified code: ① antioxidant, ② antibacterial, ③ radiative cooling, ④ atmosphere control, ⑤ superhydrophobicity, and ⑥ sensing, together with the reported shelf life extension expressed as the shelf life multiplier. Compared with packaging materials that provide barrier‐only protection (e.g., PE and CNFs), most multifunctional systems deliver a clear increase in shelf life extension, highlighting that oxygen/moisture barrier alone is rarely sufficient to counter multiple spoilage pathways. For active packaging (functions ①+②), the shelf life multiplier typically reaches a higher upper bound, and within a given food matrix it often increases—within a certain range—with improved antibacterial/antioxidant performance. Adding radiative cooling (③) or modified atmosphere regulation (④) on top of an active core can further boost preservation, consistent with the coupled suppression of microbial/oxidative deterioration and respiration‐driven quality loss. Notably, the same material system can yield markedly different multipliers across food matrices, underscoring the need to benchmark by food type. For widely studied fruits such as strawberry, barrier‐only designs typically yield modest gains (∼1.0–1.6‐fold), whereas incorporating antibacterial and antioxidant functions can expand the range to roughly ∼1–5‐fold. By comparison, modified atmosphere designs for strawberry often fall around ∼1.3–2.3‐fold, suggesting that for this high‐moisture, microbially sensitive matrix, bioactive protection is often more effective than atmosphere regulation. For mango and banana, barrier‐only systems are typically ∼1.6–1.7‐fold and radiative cooling designs cluster around ∼1.6–2‐fold, while antibacterial/antioxidant integration more frequently reaches ∼2.3–4‐fold. This suggests that under current passive cooling implementations—which often cannot maintain optimal storage temperatures—radiative cooling alone delivers limited gains relative to bioactive protection.

**TABLE 6:** Applications and benchmarking shelf life extension of various bio‐based packaging (① antioxidant, ② antibacterial, ③ radiative cooling, ④ atmosphere control, ⑤ superhydrophobicity, and ⑥ sensing).

Packaging system	Functions						The scope of application and extending the shelf life	Refs.
	①	②	③	④	⑤	⑥		
PE							Citrus/kumquat/tangerine (∼1.3‐fold); strawberry (∼1.0‐fold)	[147, 153, 155, 170]
CNFs							Banana (1.7‐fold)	[328]
Bee wax/CNFs/CNCs							Avocado (1.6‐fold); strawberry (1.6‐fold)	[45]
Corn starch/PVA							Mango (1.5‐fold)	[52]
Ferulic acid/zein/gelatin	■						Cherry (<2‐fold)	[329]
Lychee peel extract‐Al_2_O_3_ NPs/banana‐dervied bioplastics	■						Grape (1.8‐fold)	[330]
Chitosan		■					Citrus (1.2–1.5‐fold)	[147, 155, 170]
PVA/CS		■					Strawberry (1‐fold)	[325]
MOF‐545/PVA/CMC		■					Cherry tomato (1.5‐fold)	[131]
Theanine/malic acid carbon dots		■					Salmon (1.5‐fold)	[150]
BSA‐mediated crystalline Mn_3_O_4_ (nanozyme)/CNTs/Chitosan		■					Kumquat (2.3‐fold)	[147]
RC emitter/Al_2_O_3_			■				Banana (1.6‐fold); peach (2.5‐fold)	[96]
CA			■				Iced cream (2.1‐fold)	[21]
CA/DMF/TiO_2_@PT			■				Lemon slice (>2‐fold)	[89]
RC emitter/Al_2_O_3_			■				Banana (2‐fold); peach (2.5‐fold)	[96]
HNTs/PVA/AR			■				Orange (>4‐fold); tomato (>4‐fold); kiwi (>4‐fold)	[95]
P (LA‐NI)				■			Button mushroom (>4‐fold)	[120]
PL (D25/E75) LA				■			Chinese bayberry (3.5‐fold)	[121]
DE‐g‐PEI/CNF				■			Green plum (2‐fold); Litchi (2.5‐fold)	[103]
PLDC				■			Okra (>2.8‐fold)	[116]
PLGC				■			Okra (>2.8‐fold)	
MP fibrils/chitosan				■			Strawberry (1.3‐fold)	[50]
PLLA‐PCL‐PLLA				■			Strawberry (2.3‐fold)	[118]
PVA/CS/TA	■	■					Strawberry (1.7‐fold)	[325]
Thymol/γ‐CD MOFs/zein/pectin/sodium alginate	■	■					Agaricus bisporus (>2‐fold); strawberry (2‐fold)	[173]
Cinnamaldehyde/chitosan	■	■					Broccoli (4‐fold); strawberry (4‐fold)	[175]
Piceid/resveratrol	■	■					Cherry (2.3‐fold)	[176]
Curcumin/ZnO/hollow carbon nanocage/chitosan	■	■					Citrus (2.4‐fold)	[170]
Cu‐BSA nanozymes/chitosan NPs/carrageenan	■	■					Fig (3‐fold); Fresh‐cut apples (2‐fold)	[146]
Se NPs/starch/CMC	■	■					Litchi (5‐fold)	[149]
CS@citral/zein/polydopamine	■	■					Strawberry (4‐fold)	[156]
Lysozyme/sodium alginate/CNCs	■	■					Banana (4‐fold); cherry tomato (2.6‐fold); Ficus carica (3‐fold); fresh‐cut fruits (4‐fold); kiwi (4‐fold); kumquat (2‐fold); loquat (4‐fold); mango (4‐fold); nectarine (2‐fold); strawberry (5‐fold); winter jujube (1.75‐fold); wolfberry (5‐fold)	[48]
Curcumin/β‐cyclodextrin/CNFs/CNCs	■	■					Banana (3‐fold); mango (3‐fold); strawberry (3‐fold)	[45]
PVA/CS/TA@ZnO	■	■					Strawberry (2‐fold)	[325]
TA‐PS/CMC	■	■					Strawberry (2.7‐fold)	[153]
Curcumin/Fe‐MoO_x_/CS	■	■					Tangerine (2‐fold)	[155]
Bi_2_S_3_/BiOBr/BBA/gelatin/CMC	■	■					Pork (1.5‐fold)	[331]
Nitrogendoped CDs@PVA	■	■				■	Shrimp (1.7‐fold)	[332]
CA/ZnO	■	■					Strawberry (3‐fold)	[76]
CNF/TA/CA@e‐HNTs	■	■	■				Strawberry (3‐fold)	[88]
Chitosan/PVA/TiO_2_ NPs	■	■	■				Strawberry (>2.3‐fold)	[92]
TA‐CPM/shellac	■			■			Litchi (2‐fold)	[107]
Nano MOFs/CMC/Zein	■			■			Mango (2.3‐fold)	[115]
SPI nanofibers/chitosan	■	■		■			Cherry (2‐fold); strawberry (2.5‐fold)	[114]
LT‐HCOPs/PAN	■	■		■			Strawberry (3.3‐fold)	[112]
Chitosan /DCNC/CDs	■	■		■			Winter jujube (5‐fold)	[113]
Cur‐PS/chitosan	■	■		■			Cherry (>2.5‐fold); fresh‐cut apple slice (>2‐fold)	[111]
PCL/chitosan/PEO/thymol	■				■		Pork (1.3‐fold)	[60]
Chitosan/PDMS/sodium alginate/zein NPs	■				■		Fresh‐cut apple (1.5‐fold); lotus root (2‐fold)	[57]
Polydopaminehybridized@ZIF‐8/collagen/cellulose paper/PDMS (natural light)	■				■		Blueberry (3‐fold)	[160]
Polydopaminehybridized@ZIF‐8/collagen/cellulose paper/PDMS (NIR light)	■				■		Blueberry (4‐fold)	[160]
Starch nanofibers/acylated TA	■	■			■		Cherry (2‐fold); cherry tomato (1.9‐fold)	[310]
Stearic acid/anthocyanin/curcumin/TA NPs/konjac glucomannan/tea polyphenols	■	■			■	■	Fresh‐cut potato (2‐fold); pakchoi (2.5‐fold)	[317]
CMC/PVA/quercetin	■	■			■	■	Pork (2.5‐fold)	[51]

## 3 Multidimensional Evaluations for Translation and Scale‐Up

**FIGURE 8:**
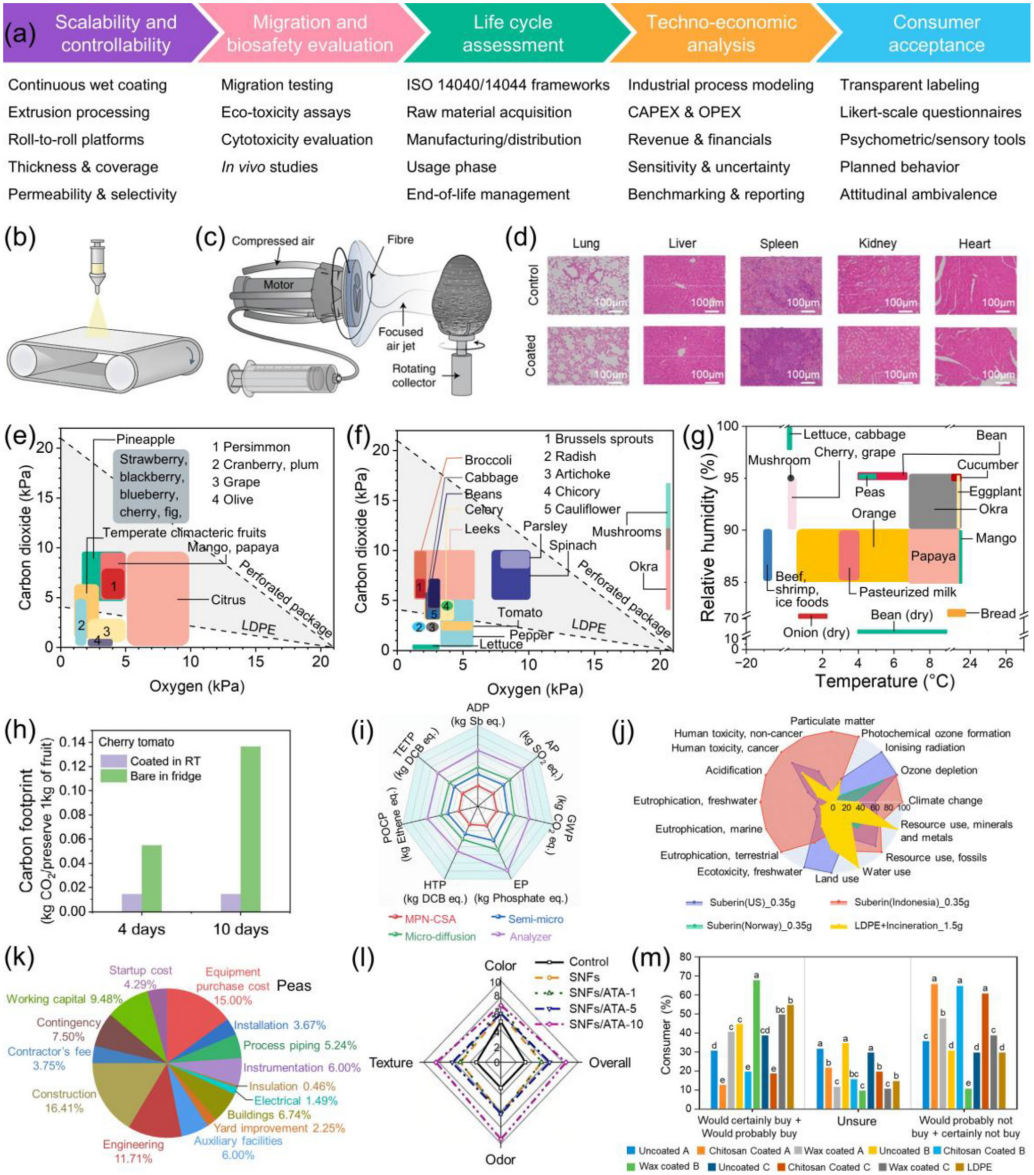
Multidimensional evaluations of multifunctional bio‐based packaging. (a) Methodological frameworks for multidimensional evaluations: scalability, biosafety, sustainability, cost‐effectiveness, consumer acceptance. (b) Roll‐to‐roll processing platforms. Reproduced with permission [379]. Copyright 2021, Springer Nature. (c) Schematic of the FRJS fiber spinning system. Reproduced with permission [139]. Copyright 2022, Springer Nature. (d) Biosafety assessment of amyloid‐like protein coatings: pathological examination results of heart, liver, spleen, lung, kidney, and intestine tissues. Reproduced with permission [149]. Copyright 2024, Elsevier. (e‐f) Recommended O_2_–CO_2_ ranges for the storage of (e) fruit and (f) vegetables (low‐density polyethylene—LDPE). Reproduced with permission [345]. Copyright 2016, AgEcon search. (g) Recommended temperature–humidity ranges for the storage of perishable foods [346, 380, 381]. (h) LCA of amyloid‐like protein coatings for preserving cherry tomatoes at room temperature (23°C and 50% humidity) compared to refrigeration at 4°C. Reproduced with permission [48]. Copyright 2025, Springer Nature. (i) Radar chart of LCA results (abiotic depletion—ADP, acidification potential—AP, global warming potential—GWP, eutrophication potential—EP, human toxicity potential—HTP, photochemical ozone creation potential—POCP, and terrestrial ecotoxicity potential—TETP) for a metal–polyphenol network CSA vs three determination processes for TVB‐N outlined in GB5009.228‐2016. Reproduced with permission [65]. Copyright 2025, John Wiley and Sons. (j) Sensitivity analysis of the LCA of suberin‐based edible coatings to electricity‐mix changes. Reproduced with permission [364]. Copyright 2023, Royal Society of Chemistry. (k) Capital investment for an industrial‐scale SPI–CNC fruit coating solution process. Reproduced with permission [71]. Copyright 2024, Elsevier. (l) Sensory evaluation of cherry tomatoes preserved using various bio‐based packaging: starch nanofibrous films (SNFs) and SNFs modified with different acylated tannic acid (ATA) loadings. Reproduced with permission [310]. Copyright 2024, Elsevier. (m) Consumer purchase intention for bread packed in various materials (different letters indicate significant differences). Reproduced with permission [74]. Copyright 2022, Elsevier.

Recent work on bio‐based packaging has moved beyond proof‐of‐concept toward multidimensional, translation‐oriented evaluation that speaks directly to deployment at scale [293]. In addition to technical feasibility, current assessments consider manufacturing scalability and controllability, migration and biosafety, life‐cycle impacts, techno‐economics, and consumer acceptance—dimensions that together determine commercial viability across food supply chains (Figure 8a). Because these dimensions are coupled by trade‐offs—particularly the complexity–cost burden of multifunctional integration—we emphasize constraint‐aware design and validation under realistic supply‐chain conditions.

### 3.1 Scalability and Controllability

Translating multifunctional bio‐based packaging from benchtop prototypes to market‐ready products requires fabrication routes that are controllable, continuous, and compatible with existing converting lines. Beyond demonstrating functions, processes must deliver predictable microstructure and food‐contact compliance at industrial throughputs, with credible energy and cost profiles. This subsection consolidates scalable unit operations, levers for structure–property control, and practically oriented guidance on in‐line quality assurance and critical scale‐up bottlenecks/risks—setting the stage for migration/biosafety (Section 3.2), LCA (Section 3.3), TEA (Section 3.4), and consumer acceptance (Section 3.5). Crucially, scale‐up is treated here as a trade‐off problem: multifunctionality should be translated into as few unit operations as practicable and as wide a controllable process window as feasible, while meeting food‐matrix‐specific performance targets and realistic constraints on throughput, yield, safety, and cost. In practice, lab‐validated performance can still fail during large‐scale manufacturing and package fabrication if materials cannot tolerate processing and handling stresses, underscoring the need to co‐design machinability and robustness alongside function [333].

#### 3.1.1 Unit Operations and Process Platforms

Solution and melt processes remain the workhorses for films, coatings, and porous membranes. For continuous wet coating, slot‐die [334, 335], gravure [336], and comma‐bar coaters [337] provide precise wet‐thickness control across meter‐scale widths and integrate readily with roll‐to‐roll (R2R) drying/curing (Figure 8b) [335]. Screen printing [338, 339] and flexography [340] support patterned deposition for localized sensing elements or Janus architectures. Spray and dip coating offer broad material latitude (solutions, colloids, emulsions, and dispersions) and suit large 3D objects (trays, lids) [45, 341], whereas doctor‐blade and curtain coating provide high‐uniformity coverage on flexible substrates (PET, PBAT, or paper) [95, 342]. On the melt side, extrusion blowing and extrusion casting convert biopolymer blends/compounds into robust films with tunable thickness and barrier performance, whereas co‐extrusion enables multilayer architectures and asymmetric wettability for active films or superhydrophobic skins [305, 343]. For fibrous layers, focused rotary jet spinning (FRJS) offers solvent‐lean, high‐throughput micro/nanofibers (≥0.2 g min^−1^ per nozzle; ≈20‐fold higher than electrospinning) for antimicrobial overcoats (Figure 8c) [139], whereas R2R electrospinning delivers sub‐micron fibers at line speeds increasingly compatible with converting lines [21]. Other scalable fiber‐manufacturing routes based on melt spinning/extrusion spinning can further support high‐area fibrous layers when materials and fiber‐size requirements permit [344].

Where radiative cooling or superhydrophobic performance depends on hierarchical roughness, scalable texturing routes—such as embossing/imprinting (using rigid or elastomeric masters) or evaporation‐induced self‐assembly—can be used to generate micro/nanotextures over large areas [57, 311, 313, 314]; for commodity trays and boxes, sprayable PRC coatings on PET or metal sheets integrate into die‐cut and folding steps without altering downstream logistics [95, 96]. A key scalability lesson is that “continuous” is defined by the slowest step along the line. For example, in R2R manufacturing routes that integrate melt processing, surface/texturing steps (e.g., nanoimprinting), and functional coatings, line speed is ultimately constrained by the bottleneck operation, so downstream coating/curing must match the throughput of upstream texturing [327]. In such cases, scale‐up is often limited by mold durability, replication fidelity, and interfacial/coating compatibility rather than proof‐of‐concept functionality.

#### 3.1.2 Structure–Function Control and Operating Windows

Reproducible function at scale hinges on linking process variables–structure–properties into actionable operating windows. Guided by the food‐matrix roadmap (Figure 1b), these operating windows should be defined against commodity‐specific targets—for example, gas‐selective transport for high‐respiration produce, moisture management for bakery goods, oxidation resistance for lipid‐rich meats/seafood, and durable water‐repellency/anti‐adhesion or anti‐icing performance for cold‐chain and frozen applications. In practice, this translates into controlling a small set of manufacturing‐critical parameters (e.g., coating weight/thickness, pore‐size distribution/tortuosity, filler fraction/dispersion, and interlayer adhesion), supported by in‐line inspection to stabilize batch‐to‐batch variability at industrial throughput.

Commodity‐specific targets can be translated into quantitative design windows (e.g., acceptable headspace O_2_–CO_2_ and temperature–humidity ranges) that reflect food physiology and use scenarios (Figure 8e–g). These design windows then define the performance specifications that must be realized through manufacturing operating windows, such as coating/film thickness, tortuosity and pore geometry, multilayer partitioning, and sealing integrity. For atmosphere control, acceptable O_2_–CO_2_ windows are strongly commodity‐dependent rather than one‐size‐fits‐all: berries (e.g., strawberry/blueberry/raspberry/blackberry) cluster in more CO_2_‐rich regimes, whereas many other fruits occupy lower‐CO_2_, low‐to‐moderate O_2_ windows (Figure 8e). Vegetables show similarly distinct groupings, with leafy and brassica‐type products (e.g., lettuce/cabbage/broccoli/cauliflower) concentrated in low‐O_2_, low‐to‐moderate CO_2_ ranges, while mushrooms (and asparagus) extend toward higher‐CO_2_ regimes (Figure 8f) [345]. Complementary to gas composition, commodities also exhibit distinct temperature–humidity design windows (Figure 8g) that govern respiration kinetics, moisture loss, decay risk, and chilling injury. The compiled recommendations cluster into a few practical regimes: many temperate fruits and leafy/brassica vegetables are best maintained near 0–2°C under high RH (∼90%–100%) to suppress metabolism while minimizing dehydration, whereas chilling‐sensitive tropical/subtropical produce shifts to higher temperatures (∼10–15°C) with moderate‐to‐high RH (∼85%–95%) to avoid low‐temperature injury. Warm‐season vegetables often favor intermediate temperatures (∼7–13°C) at high RH, while bulb/dry‐storable crops tolerate lower RH (∼65%–70%) to limit sprouting and microbial growth (Figure 8g) [346].

Translating these design windows into manufacturable operating windows places stringent demands on process control. Thickness control and drying time are repeatedly identified as scale‐up bottlenecks for film and coating technologies: inability to make large‐sized films, long drying times, and inaccurate thickness control render many lab‐scale methods unsuitable for industrial production, motivating continuous routes with shorter production time and higher production rates. Moreover, scale‐up is often constrained by integration into existing converting lines and quality‐control workflows, favoring routes that achieve target properties with minimal additional steps or equipment changes [347].

For MAP films and edible coatings, systematic control of coating parameters—most notably weight and thickness—is critical for matching commodity‐specific respiration rates and thereby maintaining freshness. Physiology‐driven thickness targets and oxygen‐barrier bands (mapping optimal thickness to respiration intensity) can be implemented through scalable coating processes (e.g., dip/spray/slot‐die coating) to match commodity‐specific respiration demands, thereby translating gas‐control requirements into actionable manufacturing operating windows. In practice, these operating windows are dominated by several manufacturing‐critical variables, most notably formulation rheology (solids/viscosity), coating or withdrawal speed (wet thickness), and drainage/drying conditions (Figure 4e,f) [45]. Radiative cooling skins exemplify how operating windows should be defined under realistic logistics rather than idealized exposure. Under such constraints, a coupled window in pore‐size distribution and high‐index filler fraction is needed to balance solar reflectivity and mechanical integrity, typically accessed via solvent‐induced phase separation, porogen leaching, and post‐aeration [21, 76, 89, 92]. Meanwhile, moisture uptake and fouling can erode reflectance, motivating protective topcoats and robust adhesion for reproducible cooling at scale. Superhydrophobic and water‐repellent layers require dual‐scale roughness and low surface energy; processing windows in curing/cooling must stabilize microtextures so repellency survives winding, sealing, and humidity cycling. For Janus designs, scale‐up is limited by the anisotropy–manufacturability trade‐off (Section 2.5), favoring converting‐compatible routes and throughput‐relevant metrics. In active systems, dose‐by‐design relies on operating windows in film/coating weight (or thickness) (g m^−2^), carrier morphology (porosity, tortuosity), and gate/cross‐link chemistry, enabling controlled loading/retention and release without compromising sealability. Intelligent labels benefit from porous or hydrogel matrices that accelerate mass transfer; windows in gelation kinetics and formulation (solids content and cross‐linker stoichiometry) tune water activity and indicator dispersion, reducing quenching and baseline drift that can drive detection errors.

#### 3.1.3 Complexity–Cost Trade‐Offs and Integration Strategies

Building on the commodity‐targeted operating windows discussed in Section 3.1.2, multifunctional integration should be evaluated as a system‐level trade‐off between performance gains and added manufacturing complexity, quality‐control burden, and unit cost. In practice, adding functions often introduces extra layers and interfaces, narrower tolerances, longer curing/conditioning steps, and stricter defect‐control requirements—effects that can amplify yield losses and increase variability even when individual functions perform well at laboratory scale. High structural precision (e.g., strong anisotropy or sharply graded architectures) often comes at the expense of throughput and yield, whereas industry favors process‐robust, converting‐compatible solutions.

To mitigate these penalties, integration can follow a “minimum‐step, maximum‐compatibility” strategy: (i) integrate multiple functions within single material building blocks (e.g., porous/hybrid fillers that both tune transport and anchor actives/indicators); (ii) adopt modular multilayers that confine costly or tightly regulated components to thin functional skins while relying on commercial base layers for mechanical strength and sealability, thereby limiting material cost and simplifying regulatory qualification; (iii) prioritize converting‐compatible, in‐line architectures over batch assembly, matching functionality to commodity‐specific targets rather than generic “highest‐performance” designs.

Importantly, tighter specifications and multilayer precision can demand stricter online monitoring and may still exceed incumbent‐plastic costs in some scenarios, motivating process simplification and explicit cost–benefit assessment alongside LCA/TEA [348]. End‐of‐life compatibility is an additional constraint, as multilayer complexity can hinder sorting and recycling and create economic challenges for recovery systems [349]. These trade‐offs also connect to well‐documented scale‐up barriers for film technologies, where time‐ and energy‐intensive processing steps can dominate feasibility and should be minimized wherever possible [347].

### 3.2 Migration and Biosafety Evaluation

For bio‐based packaging to progress from laboratory prototypes to commercial products, safety substantiation must advance in lockstep with performance validation. A key point is that “bio‐based” does not automatically mean “bio‐safe.” Many systems incorporate non‐bio‐derived additives, cross‐linkers, solvents, photoinitiators, or other functional components introduced for performance, processability, or device integration, and these constituents (or their degradation products) may migrate into food. Moreover, even “natural” components such as plant essential oils can increase consumer exposure when intentionally released from packaging and may remain toxic toward non‐target organisms [178], underscoring the need to evaluate both human health and broader ecological impacts.

#### 3.2.1 Migration Testing

Migration testing is the primary gateway requirement because it links material design to realistic exposure, demonstrating that functional constituents (e.g., EOs, nanofillers, chromogenic/fluorogenic indicators, hydrophobic components) remain sufficiently immobilized under intended use such that migration neither compromises food quality nor exceeds regulatory limits [350, 351]. This is typically implemented via standardized protocols that apply representative food simulants (e.g., aqueous ethanol, acetic acid, and vegetable oil) selected to emulate major food classes (aqueous, acidic, alcoholic, and fatty foods) under use‐relevant time–temperature profiles, including worst‐case conditions such as high‐temperature processing, long‐term storage, accelerated aging, and repeated‐use cycles [351, 352, 353, 354]. Migration results are benchmarked against regulatory thresholds, including overall migration limits (e.g., 10 mg/dm^2^ or 60 mg/kg in EU Regulation No. 10/2011) and substance‐specific migration limits for regulated species (e.g., Cu: 5 mg/kg; Zn: 5 mg/kg; Fe: 48 mg/kg) [354, 355].

Material characteristics fundamentally govern migration pathways and rates. For nanoparticles, smaller sizes generally migrate more readily—for example, 4 nm AgNPs showed 52% migration into 3% acetic acid compared with 0.17% for 41 nm particles [356]. Hydrophobic components such as waxes and fatty acids preferentially partition into fatty simulants, with migration influenced by chain length, degree of unsaturation, and crystallinity [357]. The polymer matrix also plays a decisive role: hydrophilic, low‐crystallinity, or porous matrices can promote higher mobility of small molecules and ions through water uptake and plasticization, whereas non‐polar, highly crystalline polymers typically retard transport [351].

Critically, migration is a dynamic process strongly modulated by external conditions—temperature, humidity, pH, and light exposure—which can accelerate release and, in some cases, change chemical forms and toxicological relevance [179, 182, 351]. Temperature is often the most direct accelerator: for volatile actives such as essential oils, elevated temperature increases volatility and diffusion/partitioning [178]; for metal nanoparticles, higher temperature enhances polymer‐chain mobility and diffusion and can also promote dissolution processes, jointly increasing migration [179]. Humidity is particularly important for moisture‐sensitive systems: for MOFs, water ingress can promote hydrolysis of coordination bonds and ligand displacement, and more broadly water activity can affect nanoparticle stability and migration in packaging environments [182]. pH can be decisive by altering stability and speciation: acidic conditions enhance dissolution of many metal‐based nanoparticles into ions (with substantially higher silver release reported in acidic simulants such as 3% acetic acid) [358], while for MOFs protons can protonate labile linkers (e.g., imidazolate linkers in ZIF‐8), triggering rapid framework collapse and extensive metal‐ion release—making acidic simulants among the most stringent conditions for MOF migration assessment [182]. Acidic environments can likewise promote dissolution of metal‐based nanozymes, increasing the pool of migratable ionic species [183]. For carbon dots, pH shifts can change surface state/charge and thereby influence dispersion, partitioning, and release during contact [180]. Finally, light exposure (including UV) can introduce chemistry‐coupled pathways: irradiation may accelerate polymer aging and, for photoactive oxides, affect surface redox chemistry and ion release; for carbon dots, photodegradation can generate toxic byproducts, implying that identical migrated doses may differ in hazard depending on illumination history [180].

#### 3.2.2 Biosafety Evaluation

Because chemical migration alone does not capture the full biosafety envelope, a tiered toxicological framework is increasingly adopted, spanning environmental, cellular, and systemic levels. Conceptually, migration quantifies exposure potential, whereas biosafety evaluation determines whether such exposure is acceptable under realistic use. For multifunctional packaging, the relevant hazard space must be defined by the specific functionalities and exposure scenarios involved. Common toxicological endpoints include oxidative stress, inflammatory responses, membrane and mitochondrial injury, and genotoxicity. Additional considerations arise depending on the material design: nano‑enabled systems raise concerns about cellular uptake, translocation across biological barriers, organ distribution, and the potential for chronic low‑dose oral exposure [358]; photoactivated systems may generate reactive oxygen species and transformation products under illumination—endpoints not captured by conventional dark‐condition assays and therefore requiring dedicated phototoxicity evaluation [181, 358]. These considerations motivate a tiered testing strategy that balances throughput, mechanistic relevance, and regulatory confidence.

In practice, the required endpoints and test depth are strongly material‐dependent. Metal‐based nanoparticles can induce oxidative stress and inflammatory signaling in a size‐, coating‐, and shape‐dependent manner; therefore, biosafety assessment should not treat “Ag” or “ZnO” as single entities but should report physicochemical descriptors and, where possible, relate hazards to the migrating form (ion vs particle) [179]. Nanozymes require dual assessment of material exposure and catalytic reactivity, because leachates may retain enzyme‐like activity under simulated gastrointestinal conditions. MOF‐enabled systems require explicit attention to degradation products (metal nodes/linker fragments) and residual synthesis solvents rather than assuming intact frameworks are the only relevant species. Carbon dots warrant scrutiny of precursor residues, surface charge, and light stability, since photodegradation products may introduce risks not present in dark storage [180]. Even biopolymers generally regarded as biocompatible (cellulose, chitosan, proteins) can release cross‐linkers, plasticizers, oxidation products, or non‐intentionally added substances (NIAS), making extractables/leachables profiling essential. Hydrophobic wax/fatty‐acid layers are typically of lower toxicological concern, yet they can shift effective dose by partitioning into fatty foods and by modulating the release of other actives [179, 359]; accordingly, biosafety data should be interpreted together with migration kinetics and, where relevant, sensory thresholds.

To translate the above material‐dependent endpoint selection into actionable evidence, biosafety evaluation is commonly implemented as a tiered framework that couples exposure‐informed extraction. At the environmental level, assays using model organisms probe ecotoxicity of packaging extracts or wash‐off materials. Seed‐germination tests, for instance, assess ecotoxicity through normal bean‐sprout growth and morphology in film extracts; such results support environmentally sound disposal or reuse pathways [51, 70, 149, 310]. This tier is particularly relevant for biodegradable and coated materials, where end‐of‐life leakage and surface wash‐off may represent realistic exposure routes, and where “natural” actives can still exert non‐target toxicity.

At the cellular tier, extract‐based assays (e.g., MTT, CCK‐8) quantify mammalian cell viability after exposure to packaging extracts and provide early screening for potential human‐health hazards [19, 70]. These are often complemented by mechanistic readouts aligned with dominant nanotoxicity pathways (e.g., ROS generation, pro‐inflammatory signaling, mitochondrial dysfunction, and genotoxicity markers), especially for metal‐based nanoparticles and catalytic actives [179, 358]. For photoactivated systems, illumination‐dependent testing is advisable because UV/light can amplify ROS‐driven damage for certain metal oxides and can induce photodegradation of carbon dots, generating toxic byproducts not observed under dark conditions [180, 360, 361].

At the systemic tier, in vivo studies provide organism‐level evidence and align safety evaluations with regulatory expectations. Rodent oral‐gavage studies are most common; for example, oral administration of packaging‐film extracts to mice for 14 days produced no changes in body weight, behavior, or organ histology, indicating systemic tolerability (Figure 8d) [48, 149, 176]. Complementary sensory assessments (off‐odor/off‐taste) and analytical profiling (targeted and untargeted GC/LC‐MS for extractables and NIAS) further de‐risk consumer acceptance and compliance. Importantly, the key evidence gap for packaging—particularly for nano‐enabled and catalytic actives—is chronic, low‐dose, repeated oral exposure, which is more representative of real consumption patterns than acute high‐dose testing yet remains underreported; filling this gap is essential for regulatory readiness and public trust.

Collectively, migration testing together with ecotoxicity, cytotoxicity, and in vivo evaluations constitutes a regulatory‐ready safety pipeline. Notably, studies from 2020–2025 reflect a shift from evaluating packaging solely on freshness preservation toward holistic compatibility with ecological systems, human health, and regulatory frameworks—the combination that ultimately determines scalability and adoption in real‐world supply chains.

### 3.3 Life Cycle Assessment

Bio‐based packaging is often biodegradable and may offer end‐of‐life advantages, but sustainability cannot be inferred from biodegradability alone [45, 49, 50, 111, 115, 362]. Upstream unit operations—feedstock cultivation or extraction, monomer and additive production, solvent use, thermal processing, and distribution—can introduce substantial energy demand and emissions that offset end‐of‐life or use‐phase gains. A packaging–food system perspective is essential because packaging exists to protect food, and reducing impacts at one life‐cycle stage can shift burdens elsewhere (e.g., by compromising preservation performance and increasing food waste). Moreover, decisions made during the design phase can determine a large share of life‐cycle impacts, motivating early integration of environmental evaluation rather than post hoc claims [363].

#### 3.3.1 LCA Scoping and Framework

Rigorous LCA prior to scale‐up is required to verify net environmental gains under realistic supply‐chain contexts, using ISO 14040/14044–aligned cradle‐to‐grave frameworks that quantify impacts across raw‐material acquisition, manufacturing and distribution, use phase, and end‐of‐life management [48, 65, 71, 364, 365]. However, outcomes are highly sensitive to system boundaries, functional units, allocation rules, energy sources, and end‐of‐life modeling. Therefore, credible sustainability claims require context‐specific, category‐resolved LCA supported by sensitivity/uncertainty analysis (e.g., land‐use change, yields, collection rates, and recycling/composting scenarios) [366]. A representative case illustrates this sensitivity: a cradle‐to‐grave LCA of bio‐based PET bottles reports that baseline bio‐based routes can perform worse than petrochemical PET in most impact categories, approaching parity only under favorable feedstock and process scenarios (e.g., straw‐based pathways) [367]. This underscores the need to transparently report scenario assumptions (and to conduct sensitivity/uncertainty checks where feasible) when making sustainability claims.

#### 3.3.2 LCA Findings and Implications

Building on the above scoping principles, recent LCAs reveal both opportunity and variability in bio‐based multifunctional packaging, and help identify key hotspots and actionable implications for translation. On the opportunity side, amyloid‐like protein coatings applied at room temperature reduced CO_2_ emissions by approximately 90% relative to 4°C refrigeration, while extending cherry tomato shelf life by 2.5‐fold—underscoring concurrent performance and sustainability benefits (Figure 8h) [48]. By contrast, multiple comparisons caution against assuming inherent environmental superiority: ChNF/carboxymethylcellulose films exhibited slightly higher impacts than PET [368], edible films often showed higher burdens than fossil plastics across multiple categories [365], bark‐ or peel‐derived coatings exceeded the impacts of low‐density polyethylene (LDPE) in some scenarios [364], and CNC/SPI coatings presented a mixed profile (higher global warming potential but lower cumulative energy demand than ethanol‐based paraffin wax coatings) [71]. A recurring hotspot across these studies is manufacturing: energy‐intensive thermal steps associated with film/coating formation can dominate cradle‐to‐gate burdens, making process choices—reducing water/solvent load, adopting energy‐efficient continuous routes, and decarbonizing heat/electricity—primary levers for improvement and directly linking LCA to the controllability/scale‐up discussion in Section 3.1 [369, 370].

Intelligent packaging adds further complexity because sensing/electronic modules alter inventories and end‐of‐life profiles. Assessments of electrical sensor platforms have begun to quantify fabrication footprints and to benchmark against conventional, non‐sensing formats [371, 372]. A broader comparison by Cheng et al. showed that a metal–polyphenol network CSA for TVB detection yielded lower impacts than three standard analytical methods across abiotic depletion, acidification, global warming, eutrophication, human‐health damage, photochemical ozone creation, and terrestrial ecotoxicity, highlighting the potential for low‐burden, bio‐based diagnostics within packaging (Figure 8i) [65]. At the system level, smart packaging sustainability is often packaging–food system dependent, because preservation and monitoring can translate into avoided food waste and thus offset added material burdens. Scenario‐based assessments illustrate this coupling: incorporating sensing/active components can increase the production footprint (e.g., higher impacts at the production stage), yet when use and end‐of‐life stages—and the contained food—are included, smart systems can become environmentally preferable in multiple impact categories under realistic food‐waste scenarios, while some categories may remain worse due to sensor‐related burdens [371, 373].

Collectively, these findings show that sustainability outcomes are strongly scenario‐ and process‐dependent, and often require evaluation at the packaging–food system level, motivating category‐resolved LCA with clearly stated functional units, boundaries, allocation choices, and scenario assumptions (Section 3.3.1). Accordingly, bio‐based packaging is increasingly judged by quantified environmental performance rather than a “green promise,” positioning robust LCA as a practical tool not only for regulatory dossiers but also for credible Environmental, Social, and Governance (ESG) reporting, supply‐chain decision‐making, and consumer trust (Figure 8j).

### 3.4 Techno‐Economic Analysis

While environmental performance is essential, commercial adoption also depends on cost‐effectiveness under realistic manufacturing and supply‐chain constraints. TEA complements LCA by quantifying capital and operating expenditures (CAPEX/OPEX), throughput and yield, and minimum selling price (MSP), thereby benchmarking market competitiveness for multifunctional bio‐based packaging.

#### 3.4.1 TEA Workflow and Reporting Essentials

Unlike ISO‐standardized LCA, TEA is not governed by a single universally accepted standard; however, recent practice guidance converges on a structured workflow that improves transparency and comparability: (i) scenario and market definition, (ii) process modeling with mass/energy balances for bottom‐up cost estimation, (iii) cost and profitability metrics (e.g., MSP, payback), and (iv) sensitivity/uncertainty and risk analysis to test robustness under scale‐up assumptions (e.g., n^th^ plant) and indirect‐cost treatments [374, 375]. For packaging‐relevant TEA, reporting should be normalized to practical units (e.g., US$ kg^−1^ and/or € m^−2^), accompanied by throughput/line‐speed limits, yield/reject rates, and cost decomposition that identifies dominant contributors (raw materials, utilities—especially drying/curing energy—labor, quality‐control/validation, compliance, and logistics). Such decomposition turns “economic feasibility” into actionable guidance for process simplification and scale‐up planning, and aligns with the complexity–cost trade‐offs summarized in Section 3.1.3. Using consistent inventories and scenarios when TEA is interpreted alongside LCA further reduces burden shifting and supports decision‐making across competing objectives.

#### 3.4.2 Case Studies and Gaps for Translation

Recent case studies demonstrate how TEA can inform translation and identify dominant cost drivers. For example, a TEA of a 25 Mt day^−1^ SPI/CNC fruit coating plant estimated a capital investment of US$3.68 million and annual operating costs of US$4.70 million, yielding an MSP of US$0.59 kg^−1^—competitive with wax coatings and supportive of commercial rollout [71]. Cost decomposition indicated that fixed equipment and infrastructure dominated expenditures (86.23%), with working capital and start‐up validation comprising smaller but essential shares to ensure operational continuity and regulatory compliance (Figure 8k). In a separate study, electrospun vanillin@zein antimicrobial membranes for active packaging were projected at ∼550 m^2^ day^−1^ throughput with a selling price of 9–13€ m^−2^—lower than waxed paper discs for meat products—under a business‐to‐business model, indicating potential market viability [72]. Beyond packaging‐specific examples, integrated TEA–LCA studies further illustrate how full evaluation can be strengthened by explicit sensitivity/uncertainty treatment to test robustness against key process and cost assumptions [369].

These detailed TEAs not only benchmark competitiveness but also highlight cost drivers that guide process optimization, resource allocation, and investment strategy. However, full TEA is data intensive—requiring integrated inputs on raw‐material costs, capital depreciation, labor, utilities, logistics, and market volatility—so comprehensive TEA studies remain scarce across many multifunctional bio‐based packaging concepts, despite the central role of economic feasibility in adoption. In the absence of full TEA, preliminary economic assessments can still provide decision‐relevant reference points for prioritizing designs and de‐risking pilot trials. For instance, the MPN‐CSA platform reported a laboratory‐scale cost of US$0.0348 per test, and amyloid‐like protein coatings were estimated at ∼US$0.09 per kilogram of fruit, offering early evidence of potential cost‐effectiveness and scalability [48, 65]. Although less rigorous than full TEAs, these streamlined cost screens remain informative when assumptions are stated transparently. Notably, even such preliminary economic studies are still lacking for several emerging functions, most importantly radiative cooling packaging and bio‐based MAP films/coatings. This gap motivates dedicated TEA—following the workflow outlined above—to validate translation potential and identify dominant cost levers for scale‐up. Where data are still limited, a structured screening‐level economic assessment can serve as a first pass within the same workflow—reporting rapid baselines (e.g., MSP)—and then be progressively upgraded to full TEA as process and scale‐up data mature.

Overall, economic evaluation should be treated as a core design constraint alongside LCA, shaping material selection, process design, and manufacturing strategy so that bio‐based packaging innovations are not only environmentally and technically sound but also financially viable at scale.

### 3.5 Consumer Acceptance

#### 3.5.1 Importance and Determinants

Consumer acceptance is a decisive determinant of market adoption for bio‐based packaging, complementing technical performance, environmental credentials, and cost. Perceptions of naturalness, safety, and sensory impact shape retailer uptake, brand trust, and willingness to purchase; skepticism or limited awareness can stall diffusion even when technical efficacy is established [73, 376, 377]. A salient example is the discontinuation of wax‐coated apples by major Australian retailers despite clear spoilage‐reduction benefits, underscoring that technological feasibility alone is insufficient without deliberate attention to user perception and communication [377].

Experimental evidence indicates that acceptance is malleable and can be improved through targeted message framing and ingredient selection. Informing consumers about functional benefits—waste reduction, safety, and sustainability—significantly increases acceptance of coated apples, with sustainability‐oriented messages proving especially persuasive. Ingredient transparency also matters: coatings formulated from “digestible and natural” inputs (e.g., rice starch) are preferred over insect‐derived shellac, reflecting sensitivity to perceived origin and edibility [377]. In bread applications, wax–chitosan coated paper both preserved freshness and elicited higher purchase intention (68%) than conventional LDPE plastic (55%), indicating that perceived environmental and quality benefits can outweigh reliance on familiar petrochemical packaging (Figure 8m) [74]. Collectively, these findings suggest broad application potential across categories when benefits are communicated clearly and materials align with consumer expectations of naturalness and safety.

#### 3.5.2 Assessment Methods and Intervention Strategies

Methodologically, recent studies employ structured, theory‐informed approaches to quantify and interpret acceptance. Common tools include Likert‐scale questionnaires assessing purchase intention, attitudes, and overall acceptance [73, 377]; controlled variation of information framing (functionality, sustainability, naturalness) [376, 377, 378]; psychometric measures such as the Food Technology Neophobia (FTN) scale to capture resistance to novel food technologies [377]; and sensory techniques (e.g., check‐all‐that‐apply, CATA) to profile texture, taste, and appearance perceptions (Figure 8l) [74, 376, 378]. Analyses are frequently grounded in the theory of planned behavior and diffusion of innovation, with attitudinal ambivalence used to explain mixed evaluations when perceived benefits and concerns co‐exist [73]. Demographic covariates (age, gender, education) and simulated purchasing scenarios further enhance external validity and reveal heterogeneity in responses [73, 74].

Intervention strategies suggested by this evidence include transparent labeling and clear communication of environmental and safety benefits, which reliably improve acceptance, including among consumers with higher neophobia. Preference tends to favor plant‐based, readily understandable ingredients, reinforcing the value of material choices that align with lay notions of “natural.” Third‐party certification or public‐sector endorsement can augment credibility. Critically, integrating consumer feedback early—during material selection, sensory tuning, and message design—reduces downstream adoption barriers and aligns product attributes with market expectations. In sum, strategically cultivating consumer acceptance transforms bio‐based packaging from a laboratory innovation into a socially validated solution, enabling its quality‐ and sustainability‐related benefits to be realized at scale.

## 4 Summary and Perspective

Multifunctional bio‐based packaging has advanced from additive, single‐purpose barriers to structure‐informed platforms that couple preservation with real‐time quality monitoring. This maturation shifts the emphasis from lab feasibility to supply‐chain readiness: film‐ and device‐ready architectures—integrating material chemistry, hierarchical structure, and, where appropriate, electronics and AI analytics—must be evaluated together with scale‐up, safety, sustainability, cost, and user acceptance (Figure 1). Across radiative cooling, modified atmosphere control, active systems, intelligent indicators, and superhydrophobic interfaces, recent studies report credible gains in shelf life, quality retention, and diagnostic fidelity while increasingly aligning materials choices with circularity via renewable polymers and upcycled fillers.

A unifying food‐matrix‐guided principle emerges (Figure 1b,c): preservation and sensing are most effective when co‐engineered through explicit structure–transport–function relationships. Priorities are food‐matrix dependent: high‐respiration produce is primarily governed by atmosphere–temperature windows (favoring MAP/permeability tuning, often complemented by bioactive protection), whereas lipid‐rich meats/seafood are dominated by coupled microbial and oxidative spoilage (favoring antioxidant/antimicrobial cores plus reliable freshness sensing). Moisture‐sensitive bakery goods require moisture management and anti‐adhesion, while cold‐chain/frozen products benefit disproportionately from robust water‐repellency/anti‐icing and logistics‐tolerant thermal buffering. Against this backdrop, recent advances can be distilled into the following design principles and structure‐enabled mechanisms across functional categories. In radiative cooling, matrix vibrational modes (e.g., cellulose acetate, chitosan) combined with multiscale porosity and high‐index fillers (TiO_2_, Al_2_O_3_, ZnO) maximize mid‐infrared emissivity and solar reflectance, while radiative–evaporative hybrids can raise the cooling ceiling under favorable conditions. In MAP, CO_2_/O_2_ selectivity is governed by the solubility–diffusivity landscape and pore geometry; three complementary levers—biomimetic pores (porous microspheres, MOFs, diatomite), nanoparticle/protein‐fibril hybrids that raise tortuosity, and polymer‐architecture engineering without fillers—enable commodity‐specific gas windows under variable loads and climates. Active packaging is dictated by the chemistry/stability of actives and the trigger–release architecture: robust actives (EOs, nanozymes, metal nanoparticles, carbon dots) and porous carriers (e.g., MOFs, COFs) support high loading and spatiotemporally controlled dosing, provided that phase morphology preserves mechanical integrity and sealability. Intelligent packaging transduces biomarker dynamics (TVB‐N, BAs, H_2_S, VOCs) into optical/electrical readouts using leach‐resistant immobilization, ratiometric luminophores, and array chemistries; accuracy is strengthened by engineered sampling (inclined collectors, microneedles), digitized readouts, and machine‐learning analytics for calibration, drift correction, and decoding. Superhydrophobic systems show that durable repellency is obtained only when low‐surface‐energy chemistry is coupled to hierarchical topography and interfacial adhesion is engineered; Janus/multilayer constructs further decouple conflicting requirements to co‐deploy repellency with sensing, antimicrobial, icephobic, and UV‐shielding functions.

Despite rapid progress, translation is now gated less by the availability of individual functions than by a set of cross‐cutting bottlenecks that define priorities for future work:

- Standardized benchmarking and quantitative validation of “synergy”: Performance remains difficult to compare across systems because test conditions, spoilage criteria, package geometry, and reporting practices differ. Moving forward, multifunctionality should be evaluated with harmonized benchmarks—such as the shelf life multiplier (Section 2.6.2 and Table 6)—stratified by food matrix and accompanied by a minimum reporting set (temperature–humidity history, package area‐to‐volume ratio, stacking/airflow conditions, and pre‐defined spoilage endpoints). In parallel, claims of synergy should be reserved for quantitatively validated cases, requiring explicit additivity baselines and component‐resolved controls (Section 2.6).
- Deployment scenarios and integration into existing converting lines: A persistent translational gap is that many multifunctional concepts still remain as bespoke laboratory prototypes, with limited articulation of deployment pathways aligned with mainstream packaging formats and industrial converting workflows. Practical implementation can be rationalized into three scalable archetypes: (a) functional skins/coatings applied onto commodity base films or paperboard to impart radiative cooling, water repellency/anti‐adhesion, or antimicrobial/antioxidant protection; (b) engineered barrier/MAP films that deliver commodity‐specific CO_2_/O_2_ exchange within standard pouches, clamshells, and flow‐wrap; and (c) localized add‐on modules (labels, pads, tray inserts) that enable active dosing and sensing without re‐engineering the primary package. Across these archetypes, translation should prioritize converting compatibility and package integrity (sealability, interlayer adhesion, and registration), while process windows, in‐line quality assurance, and cost/throughput constraints are addressed explicitly in (iii), (iv), and (vi).
- Operating‐window design that links commodity physiology to manufacturable process windows: A recurring failure mode is that designs optimized under a single laboratory condition do not generalize across foods or logistics scenarios. Priority should therefore shift to predictive operating windows connecting commodity physiology (respiration, chilling sensitivity, moisture loss, oxidation susceptibility) to packaging specifications (O_2_/CO_2_ permeability, selectivity, thickness/coat weight, pore geometry/tortuosity, sealing integrity) and to manufacturable process variables (rheology, coating speed, drying/curing). For MAP, this means translating commodity O_2_–CO_2_ design windows into stable thickness/transport specifications under temperature excursions; for PRC, windows must be defined under realistic exposure (stacking, enclosure, airflow) rather than ideal sky‐view conditions; and for superhydrophobic/Janus systems, windows must ensure repellency survives winding, sealing, abrasion, and humidity cycling.
- Real‐logistics robustness under stacking, fouling, abrasion, and humidity cycling: Field performance is often limited by degradation pathways absent from short laboratory tests: dust/contamination/biofilm fouling that erodes PRC reflectance, moisture uptake that plasticizes biopolymer matrices, and interfacial delamination that collapses Cassie wetting. Breakthroughs require durability‐by‐design: protective topcoats and adhesion engineering, self‐cleaning/anti‐fouling skins coupled to PRC and sensing layers, and standardized in situ validation with temperature/humidity logging under realistic stacking and airflow. Reporting retention metrics (e.g., reflectance/emissivity retention; contact‐angle/sliding‐angle stability; sealability after cycling) would make robustness comparable across studies.
- Safety‐by‐design: speciation‐aware migration, NIAS, and chronic low‐dose exposure: For multifunctional systems, safety cannot be inferred from “bio‐based” labels, and risk depends on dose, release kinetics, food matrix, and use conditions. A priority gap is quantifying migration in chemically and physically relevant forms (molecular, ionic, particulate) under worst‐case time–temperature–simulant conditions, coupled with extractables/leachables profiling (including NIAS). For nano‐enabled, catalytic, and photoactivated systems, endpoint selection must reflect function (e.g., phototoxicity under illumination; catalytic activity of leachates; MOF degradation products). The key evidence gap for regulatory readiness remains chronic, low‐dose, repeated oral exposure, which must be addressed with harmonized protocols (Section 3.2).
- Manufacturing simplification and cost competitiveness under converting constraints: Scale‐up is ultimately constrained by the slowest step (often drying/curing or precision texturing), and multifunctionality can amplify yield losses by adding layers, interfaces, and tighter tolerances. Priority should be given to converting‐compatible routes (R2R coating/printing, extrusion/co‐extrusion, scalable fiber/texturing processes) and to integration strategies that minimize unit operations (Section 3.1.3): multifunctional building blocks, thin functional skins on commercial base films, and modular multilayers that confine costly or tightly regulated components. Throughput‐relevant metrics—line speed, reject rate, batch reproducibility, and energy demand—should be reported alongside preservation and sensing performance.
- Scenario‐based LCA/TEA and end‐of‐life compatibility for multilayer and smart formats: Sustainability cannot be assumed from biodegradability; manufacturing energy and end‐of‐life scenarios often dominate outcomes (Section 3.3). Smart modules alter inventories and end‐of‐life profiles, making packaging–food system evaluation essential. Future work should prioritize aligned, scenario‐based LCA and TEA (energy mix, collection rates, composting/recycling scenarios, and food‐waste avoidance benefits) to identify break‐even conditions for adopting sensing or PRC modules. For multilayer and sensor‐integrated packaging, end‐of‐life‐by‐design (compatibility with sorting/recycling or controlled disassembly) should be treated as a hard constraint.
- AI‐enabled packaging: sensing and design acceleration, with governance‐by‐design: AI can improve calibration, drift correction, and multi‐analyte decoding for intelligent packaging, provided that deployment is supported by (a) representative, standardized ground‐truth datasets (microbiology/TVB‐N/sensory endpoints); (b) domain‐shift/drift robustness across devices, lighting, and backgrounds; and (c) auditable, explainable decision pipelines for regulatory and operational trust. Beyond readout, AI can also accelerate preservation‐function design (e.g., PRC and MAP) by learning composition–structure–property mappings from curated physicochemical datasets to enable filler selection, formulation optimization, and performance prediction (optics/durability for PRC; O_2_/CO_2_ transport/selectivity under temperature–humidity excursions for MAP) with uncertainty‐aware, multi‐objective down‐selection. Finally, system viability must be demonstrated through privacy/security‐by‐design and scenario‐based LCA/TEA that accounts for added cost, energy footprint, and e‐waste of sensor/RFID modules against realistic food‐waste‐avoidance benefits.

Collectively, these priorities indicate that progress will come from systems integration and validation, not isolated functionality. Accordingly, we advocate a framework (Figure 1c), guided by the food‐matrix‐informed roadmap (Figure 1b), that: (i) starts from commodity‐specific targets to define operating windows; (ii) programs functions via tunable pores, chemistries, and graded architectures; (iii) stabilizes performance through leach‐resistant immobilization, transport‐aware release, ratiometric/array sensing, and engineered sampling; (iv) fortifies interfaces and surfaces so performance survives real logistics; and (v) standardizes translation through harmonized benchmarks (Section 2.6), regulatory‐ready migration/biosafety pipelines (Section 3.2), and coupled scenario‐based LCA/TEA (Sections 3.3–3.4). Coupling this technical stack with trust‐by‐design (ingredient transparency, credible labeling, third‐party endorsement) and cross‐sector collaboration (pre‐competitive testbeds, interoperable data standards, pilot‐scale demonstrations) can de‐risk scale‐up and enable next‐generation bio‐based packaging to deliver verifiable reductions in waste and foodborne risk alongside measurable sustainability gains.

## Conflicts of Interest

The authors declare no conflict of interest.
